# The velvet protein Vel1 controls initial plant root colonization and conidia formation for xylem distribution in Verticillium wilt

**DOI:** 10.1371/journal.pgen.1009434

**Published:** 2021-03-15

**Authors:** Annalena M. Höfer, Rebekka Harting, Nils F. Aßmann, Jennifer Gerke, Kerstin Schmitt, Jessica Starke, Özgür Bayram, Van-Tuan Tran, Oliver Valerius, Susanna A. Braus-Stromeyer, Gerhard H. Braus

**Affiliations:** Department of Molecular Microbiology and Genetics, Institute of Microbiology and Genetics, University of Göttingen and Göttingen Center for Molecular Biosciences (GZMB), Göttingen, Germany; Oregon State University, UNITED STATES

## Abstract

The conserved fungal velvet family regulatory proteins link development and secondary metabolite production. The velvet domain for DNA binding and dimerization is similar to the structure of the Rel homology domain of the mammalian NF-κB transcription factor. A comprehensive study addressed the functions of all four homologs of velvet domain encoding genes in the fungal life cycle of the soil-borne plant pathogenic fungus *Verticillium dahliae*. Genetic, cell biological, proteomic and metabolomic analyses of Vel1, Vel2, Vel3 and Vos1 were combined with plant pathogenicity experiments. Different phases of fungal growth, development and pathogenicity require *V*. *dahliae* velvet proteins, including Vel1-Vel2, Vel2-Vos1 and Vel3-Vos1 heterodimers, which are already present during vegetative hyphal growth. The major novel finding of this study is that Vel1 is necessary for initial plant root colonization and together with Vel3 for propagation *in planta* by conidiation. Vel1 is needed for disease symptom induction in tomato. Vel1, Vel2, and Vel3 control the formation of microsclerotia in senescent plants. Vel1 is the most important among all four *V*. *dahliae* velvet proteins with a wide variety of functions during all phases of the fungal life cycle *in* as well as *ex planta*.

## Author summary

Verticillium wilt is a destructive disease of many economic important crop plants induced by fungal plant pathogenic *Verticillium spp*. such as *V*. *dahliae*. Developmental processes and the production of secondary metabolites are coordinated by fungal velvet domain proteins. The four *V*. *dahliae* velvet domain proteins form the heterodimers Vel1-Vel2, Vel2-Vos1 and Vel3-Vos1 during vegetative growth. Vel1, Vel2 and Vel3 coordinate the formation of the microsclerotia resting structures and the production of the metabolite melanin, which is deposited in microsclerotia. The survival of these resting structures in the soil is essential for *Verticillium spp*. during the monocyclic disease. Microsclerotia germinate in the presence of a suitable host, the resulting hyphae grow to and colonize the root. We discovered that this important process of initial colonization requires the presence of Vel1. Once inside the plant, the fungus distributes in the vascular system by conidia formation, which depends on Vel1 and Vel3. Vel1 is a promising target to control the fungal disease, because it is required for the induction of disease symptoms.

## Introduction

Many plants are destroyed by wilt causing plant pathogenic fungi such as the ascomycete *Verticillium dahliae*, which infects over 200 hosts in temperate and subtropical regions and becomes a world-wide threat [1–3]. *V*. *dahliae* remains in the soil for years without a suitable host as melanized resting structures, called microsclerotia [4]. Microsclerotia germinate after perception of root exudates and hyphae grow into the plants direction [1,5]. *V*. *dahliae* uses regions where the endodermis has not been fully developed, for instance the tip or sites of lateral root formation, for penetration when reaching the root [1,2]. The fungus can invade the plant by hyphopodia, which are the swollen tips of hyphae [6]. *V*. *dahliae* starts conidiation inside the vascular tissue and uses the sap stream to distribute within the plant [2,7]. Germinating conidia in trapping sites may plug the transpiration stream and the plant initiates a defense reaction, which the fungus tries to escape by progressive colonization of neighboring cells. Nutrients and water transport to the shoots can be disturbed [1,2,8,9], resulting in disease symptoms like chlorosis, necrosis, stunted growth and wilting. *V*. *dahliae* forms microsclerotia in senescent plants, that reach the soil with the fading plant and remain there until a new host is present [4,7,9].

Fungal development, resting structure formation and secondary metabolite production are connected to the velvet family of regulatory proteins [10]. The first velvet family member, velvet A (VeA), was described in the filamentous ascomycete *Aspergillus nidulans*. A strain with a point mutation in the *veA* gene resulted in a truncated VeA protein and produced more conidia but fewer fruiting bodies [11]. VeA coordinates development with secondary metabolism [12–15]. The *A*. *nidulans* velvet family includes four members: VeA, velvet like B (VelB), velvet like C (VelC) and viability of spores A (VosA) [11,16–18]. VeA is a light-dependent activator of sexual development and secondary metabolite production [12,19,20]. In contrast, *Neurospora crassa* VE-1 functions light-independently. VE-1 promotes hyphal growth, protoperithecia formation and secondary metabolism, but reduces asexual conidiation [21,22]. Light-dependent processes such as macroconidia and sclerotia formation as well as melanin biosynthesis are regulated by VEL1 of the plant pathogen *Botrytis cinerea*. The corresponding *vel1* gene is required for virulence, but not for the production of virulence-related secondary metabolites as botcinic acid and botrydial [23]. Similar functions as for VeA were assigned to VelB in *A*. *nidulans* or other ascomycetes [17,24]. *A*. *nidulans* VelC functions as positive regulator of sexual development and controls genes involved in asexual development. In several fungi *velC* deletion leads to increased conidia formation, its role in asexual development might therefore be conserved [25]. *A*. *nidulans* VosA promotes spore viability, trehalose biosynthesis and control of secondary metabolite genes [16,26].

Velvet proteins comprise a velvet domain with proline residues in the middle of the motif but without sequence similarity to other known domains [10]. The first identified velvet complex was trimeric and consisted of *A*. *nidulans* VelB, VeA and the secondary metabolite methyltransferase LaeA [17]. Folding of the fungal velvet domain is similar to the structure of the mammalian REL homology domain in the transcription factor NF-κB including conserved amino acids for DNA contact [27]. Velvet proteins form different heterodimers and interact with various methyltransferases as part of the fungal transcriptional and epigenetic control [10,18,27,28]. The VeA-VelB heterodimer activates sexual development and positively regulates secondary metabolite production. Spore viability and trehalose biosynthesis are controlled by the VelB-VosA heterodimer. Other interactions, for instance between VelC and VosA, are known, but the function remains ambiguous [28].

*A*. *nidulans* VosA has a transcription activation domain [16] and binds over 1500 promoter sequences including promoters of regulatory genes such as the zinc cluster transcription factor SclB, which controls its own regulatory subnetwork [27]. SclB has repressing functions for developmental processes, stress response and secondary metabolism [29]. Velvet proteins therefore represent a class of fungal transcription factors with impact on different biological processes. Tight control mechanisms and extensive links to signaling pathways are required because velvet proteins have important functions in cellular development. VeA protein stability is controlled by the deubiquitinase UspA [30]. Moreover VeA interacts with light receptors such as FphA and is connected to the mitogen activated protein kinase MpkB [28].

Velvet proteins, which are conserved in ascomycetes and basidiomycetes [16], have to be adapted to the life cycles of highly different fungi and are potentially interesting targets for the development of new antifungals. Growth of vascular plant pathogens as members of the genus Verticillium is especially difficult to control. They can be hardly attacked in the plants xylem system where they propagate as asexual spores. In the soil, their microsclerotia are resilient long-term survival structures, which are formed in senescent plants at the end of the growing season.

This study aimed to reveal the yet elusive functions of the velvet proteins in the life cycle of *V*. *dahliae* plant vascular pathogens. The goal was to address whether any of the velvet protein encoding genes is a potential target to combat the fungus outside or inside the plant. We found a prominent role in virulence for the Vel1 encoding gene, which exhibits a novel virulence-related function already at the early phase of plant root colonization. *V*. *dahliae* velvet proteins are important for microsclerotia formation and secondary metabolite production, as for instance the melanin precursor scytalone. They are also needed for fungal distribution by conidia *in planta*. Vel1 is exposed as central factor for all steps of *V*. *dahliae* development and disease induction with a novel role in root colonization.

## Results

### *V*. *dahliae* carries a similar set of four velvet domain protein encoding genes as A. nidulans

**Fig 1:**
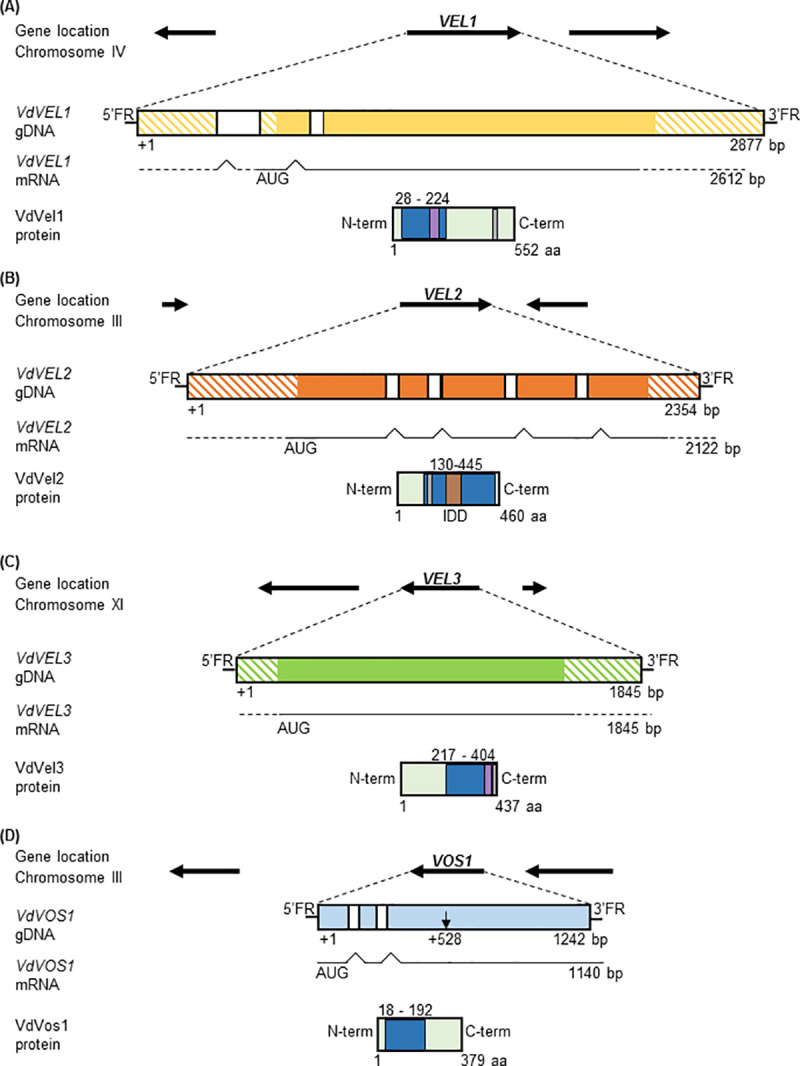
*V*. *dahliae* genes encoding velvet domain proteins. Genomic loci (gDNA) including transcription directions of neighboring genes and assignments to fungal chromosomes are indicated. Introns are depicted in white, exons are depicted in color; color hatched areas show the predicted untranslated region (UTR). Velvet domains (dark blue) were predicted by InterProScan according to entry IPR037525. Nuclear localization sequences (NLS, in grey) were predicted by using cNLS mapper. Potential PEST motifs (purple) were identified using epestfind. Details are given in S1–S4 Figs. **(A)** *VEL1* (VDAG_JR2_Chr7g04890a) consists of three exons and two introns. The deduced Vel1 protein holds a velvet domain and a PEST motif at the N-terminus and an NLS at the C-terminus. **(B)** *VEL2* (VDAG_JR2_Chr3g06150a) consists of five exons and four introns. The Vel2 velvet domain includes an NLS and is interrupted by an intrinsically disordered domain (IDD). **(C)** *VEL3* (VDAG_JR2_Chr6g00630a) consists of a single exon. The Vel3 protein has one velvet domain, a PEST motif and an NLS at the C-terminus. **(D)** *VOS1* (VDAG_JR2_Chr3g12090a) original annotation (starting point indicated by black arrow) was corrected by sequencing and verified by mass spectrometry. The corrected *VOS1* annotation results in three exons and two introns and a Vos1 protein with N-terminal velvet domain.

The four *V*. *dahliae* JR2 homolog encoding proteins with the velvet DNA binding and dimerization domain were identified by BLAST search and compared to corresponding genes from other ascomycetes (Figs 1 and S1–S4). *V*. *dahliae* velvet domains vary in length between 174 to 315 amino acids (aa). The architecture of the velvet proteins is largely conserved between *V*. *dahliae* and *A*. *nidulans*. Alignment of *V*. *dahliae* Vel2 with the *A*. *nidulans* VelB velvet domain [27] revealed that both are interrupted by an intrinsically disordered domain (S2 Fig). The genus Verticillium belongs to the Sordariomycetes, the second-largest class of Ascomycota. Aspergillus is assigned to the Eurotiomycetes [31,32]. The genera Verticillium and Colletotrichum are within their class in the same order of the Glomerellales [32]. Consistently, the highest overall amino acid sequence similarities for three deduced velvet proteins were observed between the corresponding *V*. *dahliae* and *Colletotrichum graminicola* counterparts. Only the primary amino acid sequence of *V*. *dahliae* Vos1 is more like the corresponding protein of the phytopathogenic *Magnaporthe oryzae* as another representative of the Sordariomycetes (S5A–S5D Fig).

Two proteins, Vel1 and Vos1, comprise an N-terminal velvet domain. *V*. *dahliae VEL1* (VDAG_JR2_Chr7g04890a) corresponds to the *A*. *nidulans* gene encoding the VeA regulator. *VdVEL1* includes three exons and two introns and results in a 2612 bp transcript with a deduced protein of 552 aa, which is similar in size to *An*VeA (573 aa) [10]. Besides the N-terminal velvet domain, a predicted nuclear localization signal (NLS) is located in *Vd*Vel1 as in *An*VeA at the C-terminus. Vel1 carries a PEST domain between amino acids 162–183 (Fig 1A), suggesting a specific and presumably conserved control mechanism of protein stability as described for *An*VeA [30]. The other N-terminal velvet domain is located in *A*. *nidulans* VosA corresponding to *V*. *dahliae* Vos1. The annotation of the originally identified *V*. *dahliae VOS1* gene (VDAG_JR2_Chr3g12090a) resulted initially only in a deduced rather small 237 aa protein. Sequencing of the fungal cDNA revised the annotation with a changed start codon and an additional intron. The protein sequence of this improved annotation was further verified by protein pull-downs followed by mass spectrometry (S5E Fig). The corrected *VOS1* sequence includes three exons and two introns for a 1140 bp transcript with a deduced protein of 379 aa compared to 430 aa for *An*VosA (Fig 1D).

The velvet domains of the remaining proteins, Vel2 and Vel3, are located in the C-terminal part. The *V*. *dahliae VEL2* (VDAG_JR2_Chr3g06150a) and *VEL3* (VDAG_JR2_Chr6g00630a) genes are homologs to *A*. *nidulans* genes encoding VelB and VelC, respectively (Figs 1B, 1C, S2 and S3). *VEL2* consists of five exons and four introns and a resulting mRNA of 2122 bp. *VEL3* contains only one exon with an 1845 bp transcript. Translation results in a larger *Vd*Vel2 protein (460 aa) in comparison to *An*VelB (369 aa), but *vice versa*, a smaller 437 aa *Vd*Vel3 protein compared to *An*VelC (524aa). Vel2/VelB carry a similar C-terminal velvet domain, which is interrupted by an intrinsically disordered domain (IDD) in both proteins not interfering with the folding of the velvet domain in the X-ray structure of VelB [27]. In contrast to VelB, Vel2 includes an NLS. Vel3 and VelC proteins harbor a presumed destabilizing PEST domain next to the velvet domain between amino acids 415–430 and carry the canonical NLS at the C-terminus, similar to Vel1/VeA (Fig 1C).

### Microsclerotia formation depends on Vel1, but requires Vel2 as additional positive and Vel3 as negative factors in light

**Fig 2:**
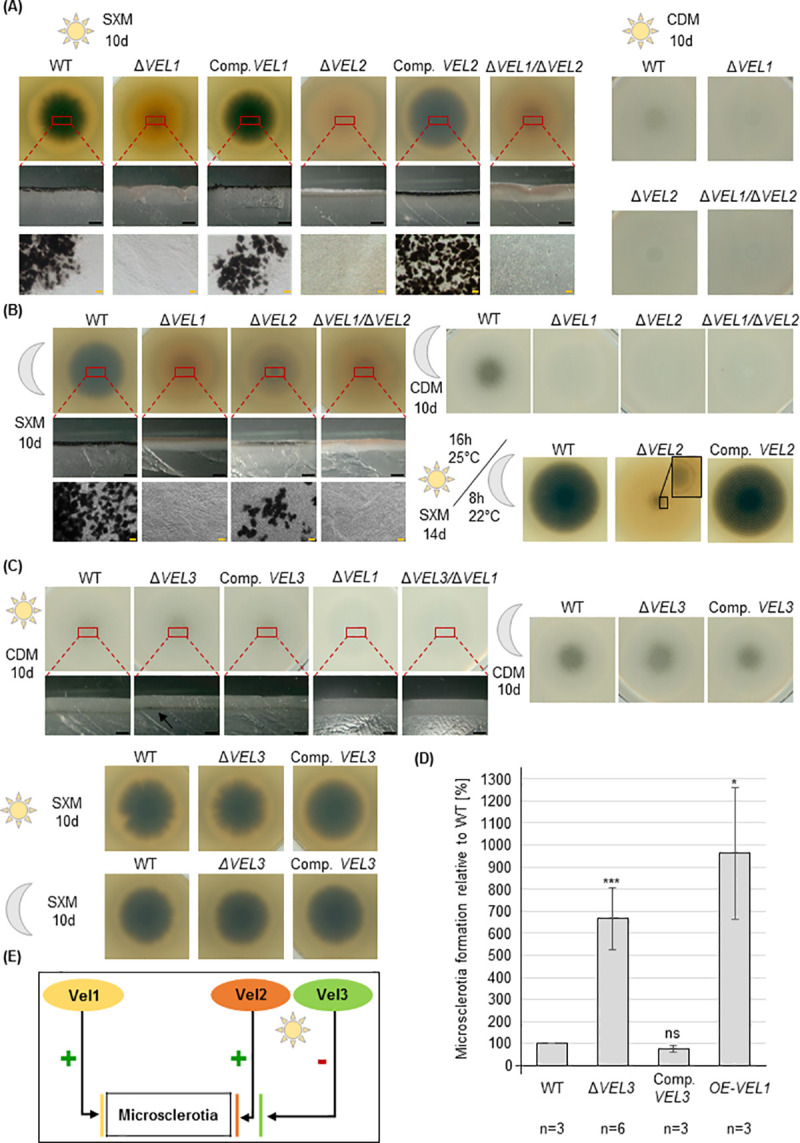
*V*. *dahliae* microsclerotia formation depends on Vel1 with Vel2 as additional positive and Vel3 as negative control regulator primarily in light. 5x10^4^ spores of the indicated strains were spotted on indicated plates (SXM: simulated xylem medium; CDM: minimal Czapek-Dox-Medium) and incubated for 10 days at 25°C. Single colonies on SXM are shown from the back and colonies on CDM are shown from the top of the plate. Cross sections were made through the middle of the colony. Microscopy images show colony material scraped from the surface. **(A)** Vel1 and Vel2 are required for microsclerotia development in the presence of light. Microsclerotia formation of the Δ*VEL1* or Δ*VEL2* strains was compared to wild type (WT) and respective complementation strains (Comp.) on single colony pictures (first row), cross sections (second row) and in micrographs (third row). **(B)** Vel1 is primarily required for microsclerotia formation in darkness with a smaller contribution of Vel2. Incubation as in (A) but in darkness instead of light. Wild type as well as the *VEL2* deletion strain produce microsclerotia in darkness, but absence of *VEL2* leads to reduced amounts of microsclerotia. Lower right part: Vel2 function is independent of the fungal light/dark control. Phenotypical analysis during long day/night cycling conditions (16 h, 25°C: 8 h, 22°C, light: dark). Wild type displays a ring-like structure around the inoculation point with melanized microsclerotia and reduced color of the microsclerotia towards the margins of the colony. The *VEL2* deletion strain is also capable to form the ring-like structure but with hardly any melanization (black box). **(C)** Vel3 inhibits microsclerotia formation in light without strong impact in dark on minimal medium. Colony pictures (first row) and cross sections (second row) are shown. Black scale bar = 1 mm, yellow scale bar = 20 μm. **(D)** Microsclerotia quantification of the wild type, the *VEL3* deletion and complementation strain as well as the *VEL1* overexpression strain. The *VEL3* deletion and the *VEL1* overexpression strains produce significantly more microsclerotia than the wild type and complementation strain. The experiment was repeated three times with three technical replicates each. Significant differences to wild type were calculated by t-test and indicate*:p<0.05; ***:p<0.001; ns: not significant. **(E)** Interplay between the velvet domain proteins Vel1, Vel2 and Vel3 in *V*. *dahliae* microsclerotia formation in the presence or absence of illumination.

Velvet proteins form homo- as well as heterodimers, which control fungal development and coordinate it with the formation of an appropriate development-specific secondary metabolism [10,17]. During senescence of the host, *V*. *dahliae* forms melanized microsclerotia for long-term survival in the soil, which can germinate even after decades in the presence of a host [4,5]. These resting structures are potential antifungal targets for crop protection against *V*. *dahliae*. Deletion strains of the four *V*. *dahliae* velvet genes as well as selected double deletion strains were constructed by replacing their open reading frames with a resistance cassette using homologous recombination (S6 and S7 Figs). All deletion strains could grow on different media with similar growth rates as wild type (Fig 2). The Δ*VEL1-3* strains affected microsclerotia formation (Fig 2), whereas Δ*VOS1* was the only exception with a wild type-like phenotype (S8 Fig).

The wild type produces microsclerotia on pectin-rich simulated xylem medium (SXM) and on minimal medium (Czapek-Dox-Medium, CDM). Microsclerotia formation of *V*. *dahliae* in light is reduced in comparison to cultivation in darkness, especially on minimal medium. Absence of *VEL1* resulted in a strain impaired in microsclerotia formation in the presence or absence of light. The resting structures are neither visible in the colony overview nor in cross sections or microscopic views. Microsclerotia production can be restored by introducing the wild type gene into the deletion strain (Fig 2A).

The phenotype of a Δ*VEL2* strain after illumination resembled the Δ*VEL1* or the Δ*VEL1*/Δ*VEL2* strain with impaired microsclerotia formation (Fig 2A and 2B). However, the Δ*VEL2* strain could produce reduced numbers of microsclerotia compared to wild type when incubated in darkness on simulated xylem medium (SXM), but not on minimal medium (CDM) (Fig 2B).

Light response can be interconnected with temperature and circadian control in fungi [33,34]. Wild type *V*. *dahliae* forms microsclerotia and aerial hyphae in response to light and dark cycles on SXM, resulting in a characteristic ring-like structure. The ring-like structure was visible in the Δ*VEL2* strain but in combination with only an initial melanization in the center and the first rings (Fig 2B). This suggests that the light-dependent Vel2 function in microsclerotia formation is independent of fungal light/dark cycle control.

The Δ*VEL3* strain produced more microsclerotia on minimal medium (CDM) than wild type in light (Fig 2C). This effect was quantified by evaluation of the brightness factor of respective colonies. We found that the relative amount of microsclerotia in the *VEL3* deletion strain is more than six times higher compared to wild type (Fig 2D). Reintroduction of the gene complemented this effect with wild type levels of microsclerotia. In darkness, microsclerotia production was not increased (Fig 2C). This light effect was not observed for SXM. Hardly any differences were visible in darkness between wild type and Δ*VEL3* strain. The *VEL1*/*VEL3* double deletion strain resembled the *VEL1* single deletion phenotype suggesting that Vel3 might require the presence of Vel1 for its light-dependent inhibitory function (Fig 2C). In contrast, a *VOS1* double deletion with *VEL3* showed a Δ*VEL3* single deletion-like phenotype (S8 Fig). In summary, Vel1 acts as a general positive regulator of *V*. *dahliae* microsclerotia production, whereas Vel2 is a light-dependent positive regulator and Vel3 has opposite functions during formation of these resting structures (Fig 2E).

### Formation of specific *V*. *dahliae* secondary metabolites is regulated by Vel1, Vel2 and Vel3

Velvet domain protein complexes coordinate fungal resting structure and secondary metabolite formation, including toxic compounds for pathogenic interactions or signaling molecules for communication with other microorganisms [10,35–37]. Secondary metabolite formation of the *V*. *dahliae* velvet deletion strains were compared to wild type and the corresponding complementation strains. All strains were grown on standard minimal medium with Glucose (CDM+Glucose), which allows conidiation as well as resting structure formation. Extracts were applied to liquid chromatography/mass spectrometry (LC/MS) combined with photodiode array detection analysis.

**Fig 3:**
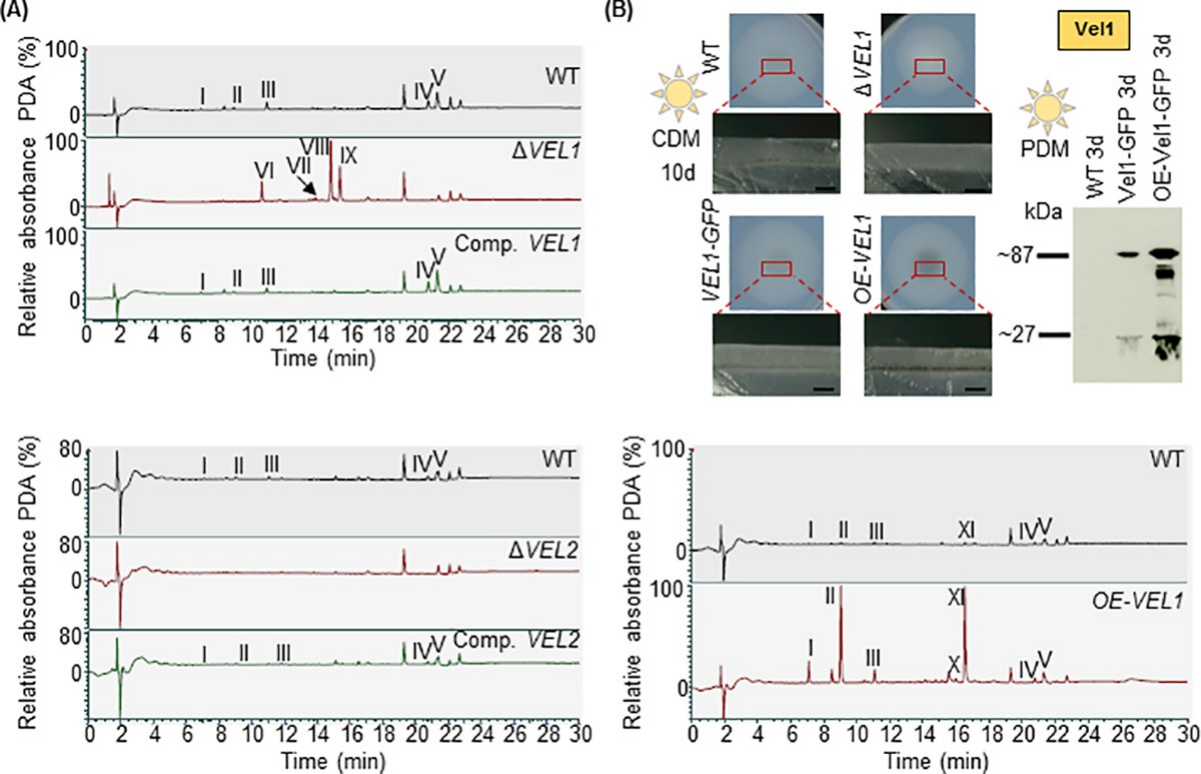
*V*. *dahliae* Vel1 and Vel2 control functions in secondary metabolite production. Displayed are chromatograms of metabolites extracted from Δ*VEL1* and Δ*VEL2* (A) and from the *VEL1* overexpression strain (*OE-VEL1*) (B). LC/MS combined with photodiode array detection (PDA) analysis is shown. Secondary metabolites were extracted from two-week-old fungal mycelium grown on Czapek-Dox-Medium (CDM) supplemented with glucose in constant light. **(A)** Upper part: Deletion of *VEL1* (Δ*VEL1*) leads to reduced formation of five substances (I-V) and production of four substances (VI-IX) which were not abundant in metabolite extracts of wild type (WT) or complementation (Comp. *VEL1*) control strains. Lower part: The same five substances (I-V), which were not detected in the metabolite extracts of the *VEL1* deletion strain, are also absent in the *VEL2* deletion strain extracts. **(B)** *VEL1* overexpression increases melanization and alters the expression of secondary metabolites. Upper part, left: Phenotypical analysis of wild type, *VEL1* deletion strain, Vel1 tagged with GFP and overexpression of Vel1 tagged with GFP. 5x10^4^ spores of the indicated strains were spotted on Czapek-Dox-Medium (CDM) and incubated for 10 days at 25°C in light. An overview of a single colony and a cross section are displayed. The *VEL1* deletion strain (Δ*VEL1*) is hindered in formation of melanized microsclerotia; in contrast the overexpression of *VEL1* tagged with *GFP* (*OE-VEL1*) melanizes more than wild type (WT) or a strain with endogenously integrated Vel1-GFP as controls. Black scale bar = 1 mm. Upper part, right: Western hybridization of wild type, Vel1 tagged with GFP and overexpression of Vel1 fused to GFP. Liquid potato dextrose medium (PDM) cultures inoculated with 1x10^6^ freshly harvested spores were grown for three days at 25°C in light. Free GFP (27 kDa) or increased Vel1-GFP (87 kDa) in the overexpression strain were detected by Western hybridization with a GFP antibody in crude extracts. Lower part: Chromatogram of secondary metabolites of wild type and *VEL1* overexpression strain extracts from two-week-old fungal mycelium grown on Czapek-Dox-Medium (CDM) supplemented with glucose. Depicted is LC/MS analysis with photodiode array detection (PDA) analysis. Seven substances (I-V, X and XI) are more abundant in *OE-VEL1* than in wild type.

Chromatograms of extracts of dark or light grown cultures revealed at least five substances (indicated as I-V), which were reduced, and at least four (VI-IX), which were increased in the Δ*VEL1* strain compared to wild type (Figs 3A and S9A). There is presumably a partial overlap in Vel1 and Vel2 functions because compounds I-V were also reduced in the Δ*VEL2* strain, whereas the other compounds were not significantly affected compared to wild type (Figs 3A and S10–S14 and Table 1). Overexpression of *VEL1* resulted in increased formation of compounds I-V and compounds X-XI (Figs 3B and S15). The enhanced formation of the protein was confirmed by western experiments (Fig 3B). A similar but less pronounced effect as seen for the overexpression of *VEL1*, was found for the Δ*VEL3* strain (S16A and S16B Fig). The *VEL1* overexpression and the Δ*VEL3* strain do not only share similarities in their metabolite profiles but also in colony color, because both show an increased melanization phenotype (Figs 2C and 3D). Determination of the colony brightness factor relative to the wild type suggested a more than nine times increased production of melanized microsclerotia (Fig 2D).

**Table 1:** Differentially expressed metabolites of *V*. *dahliae* detected by LC/MS.

ID	Metabolite marker	Retention time (min)	Detected as	Predicted sum formula	Exact mass measured	Exact mass calculated [M]	Confirmed by	Reference
I	Scytalone	7.00	[M-H]^-^	C_10_H_10_O_4_	193.0493	194.0579	MS2 spec-trum	[43,44]
II	UN	8.98	[M-H]^-^	C_12_H_12_O_5_	235.0603	236.0685		-
III	6,8-Dihydroxy-3-methyl-1H-2-benzopyran-1-one (Saccharonol A or 3,4-Dehydro-6-hydroxy-mellein)	10.98	[M+H]^+^	C_10_H_8_O_4_	193.0499	192.0423	MS2 spec-trum	[45]
IV	UN	20.76	[M+H]^+^	C_29_H_48_N_6_O_6_	577.3732	576.3635		-
V	UN	21.30	[M+H]^+^	C_30_H_50_N_6_O_6_	591.3887	590.3792		-
VI	UN	10.68	[M-H]^-^	C_15_H_20_O_4_	263.1284	264.1362		-
VII	UN	13.92	[M-H]^-^	C_18_H_24_O_5_S	351.1264	352.1344		-
VIII	UN	14.85	[M+H]^+^	C_15_H_20_O_2_	233.1544	232.1463		-
IX	UN	15.41	[M+H]^+^	C_15_H_20_O_3_	249.1483	248.1412		-
X	UN	15.99	[M-H]^-^	C_20_H_12_O_5_	331.0606	332.0685		-
XI	UN	16.52	[M-H]^-^	C_20_H_14_O_4_	317.0811	318.0892		-

UN = unknown

Deletions of *VOS1* or *LAE1*, which represents a major epigenetic secondary metabolite regulator interacting with *A*. *nidulans* velvet proteins [17], had no major impact on *V*. *dahliae* microsclerotia formation or secondary metabolism during the analyzed conditions (S8, S17A and S18 Figs).

The substances I-XI could be assigned to a mass but only two compounds resembled deposited substances in databases (Tables 1 and 2, and S10–S15, S19 and S20 Figs). Substance I was likely identified as the DHN melanin precursor scytalone [38], which is in line with the increased melanization in *VEL1* overexpression and Δ*VEL3* strains. The area of the scytalone peak in wild type, Δ*VEL3* and *VEL1* overexpression strains was measured for a relative quantification of the substance. Scytalone production was significantly increased for both mutant strains compared to wild type (S16C Fig). Melanin production in *V*. *dahliae* is amongst others regulated by the transcription factor Cmr1, which controls the expression of several genes involved in pigment biosynthesis [38]. We analyzed the expression of *CMR1* in the wild type and the *VEL1* deletion strain, which were grown for three days in a liquid potato dextrose medium (PDM) culture, a condition in which the Vel1 protein is present in the cell. The relative expression of *CMR1* is decreased to less than 20% of the wild type level when *VEL1* is deleted (S9B Fig). This suggests that the absence of microsclerotia production and scytalone synthesis in the *VEL1* deletion strain might be correlated with a decreased expression of the pigment biosynthesis transcription factor Cmr1.

**Table 2:** Metabolites detected by LC/MS in different *V*. *dahliae* strains.

ID	Retention time (min)	Exact mass measured	Predicted sum formula	Found in									
				WT	Δ*VEL1*	comp. *VEL1*	OE- *VEL1*	Δ*VEL2*	comp. *VEL2*	Δ*VEL3*	comp. *VEL3*	Δ*VOS1*	Δ*LAE1*
I	7.00	193.0493	C_10_H_10_O_4_	x		x	x		x	x	x	x	x
II	8.98	235.0603	C_12_H_12_O_5_	x		x	x		x	x	x	x	x
III	10.98	193.0499	C_10_H_8_O_4_	x		x	x		x	x	x	x	x
IV	20.76	577.3732	C_29_H_48_N_6_O_6_	x		x	x		x	x	x	x	x
V	21.30	591.3887	C_30_H_50_N_6_O_6_	x		x	x		x	x	x	x	x
VI	10.68	263.1284	C_15_H_20_O_4_		x								
VII	13.92	351.1264	C_18_H_24_O_5_S		x								
VIII	14.85	233.1544	C_15_H_20_O_2_		x								
IX	15.41	249.1483	C_15_H_20_O_3_		x								
X	15.99	331.0606	C_20_H_12_O_5_				x			(x)			
XI	16.52	317.0811	C_20_H_14_O_4_	(x)	(x)	(x)	x	(x)	(x)	x	(x)	(x)	(x)

x indicates presence of the metabolite. (x) indicates that the exact mass of the substance is detectable, but no clear peak in the chromatograms, or a small peak in some replicates is present.

Substance III is similar to saccharonol A and might be a precursor of 6-methoxymellein, which was found in endophytic and plant pathogenic species as well as fungi-infected carrot roots [39,40]. 6-methoxymellein is a precursor of terrein, which is produced by *A*. *terreus* and harms the surface of fruits [41,42]. These data support that primarily Vel1 but also Vel2 proteins specifically control secondary metabolite formation and are counteracted by Vel3. These secondary metabolites include possible functions in fungal protection by pigmentation and interactions with plants.

### Vel1 and Vel3 are both required for efficient *V*. *dahliae* conidiospore formation

**Fig 4:**
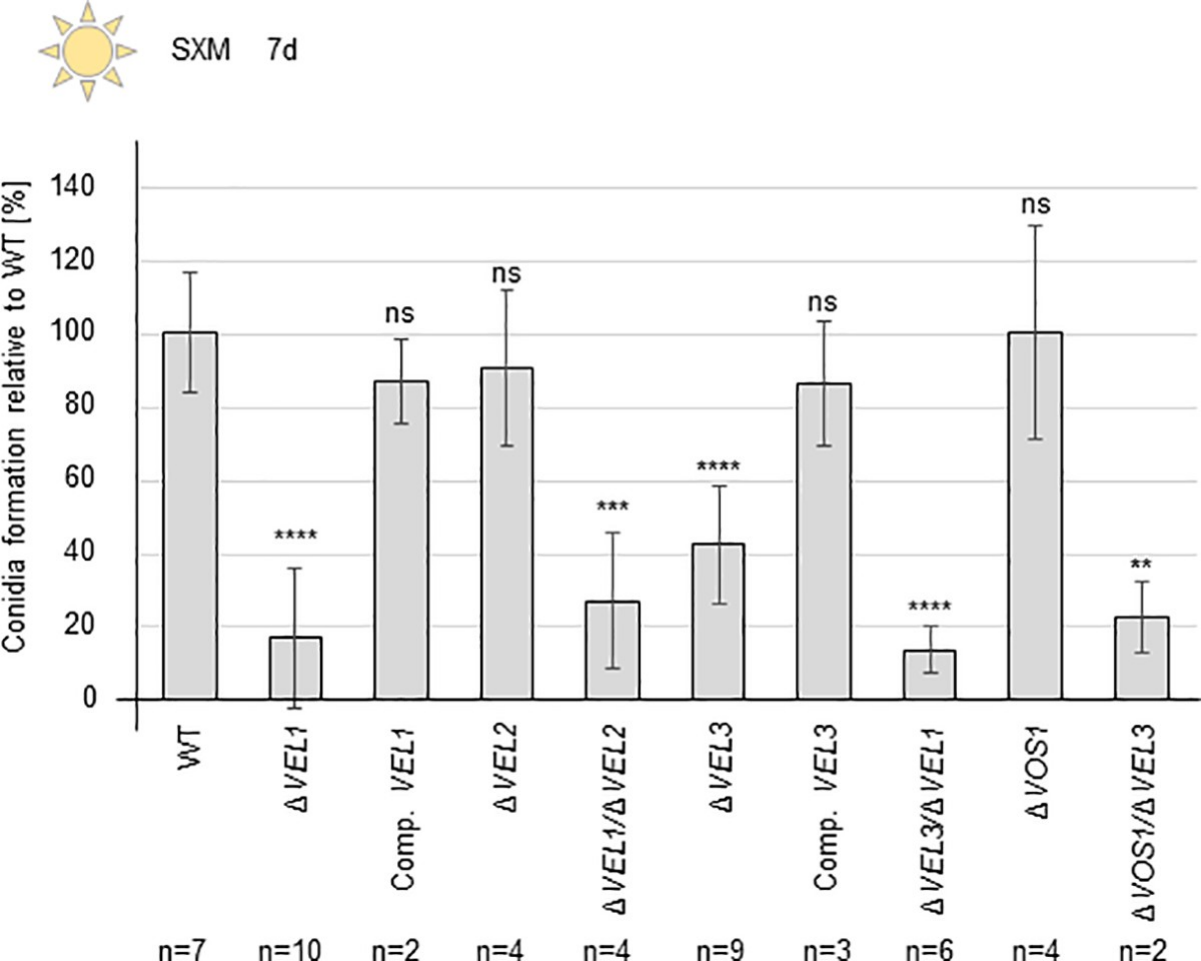
*V*. *dahliae* conidiospore formation requires the *VEL1* and *VEL3* genes. 2x10^5^ freshly harvested spores were inoculated in liquid simulated xylem medium (SXM) and incubated at 25°C for seven days under constant agitation and light. The conidia formation was quantified relative to the wild type (WT). Each experiment was performed with three technical replicates (n = 1). Two individual transformants were used for the following strains: Δ*VEL1*, Δ*VEL2*, Δ*VEL1*/Δ*VEL2*, Δ*VEL3*, Δ*VEL3*/Δ*VEL1* and Δ*VOS1*. Bars represent the mean values of all experiments and error bars correspond to standard deviations. *VEL1* and *VEL3* deletion and double deletion strains are impaired in conidia formation. *VEL2* and *VOS1* are dispensable for conidiation. Observed defects were restored in the corresponding complementation strains. Significant differences were calculated by t-test and indicate: **:p<0.01; ***:p<0.001; ****:p = 0.0001; ns: not significant.

*V*. *dahliae* asexual sporulation represents the second major developmental program, besides microsclerotia formation as resting structures. In contrast to *A*. *nidulans* and other fungi, the resulting *V*. *dahliae* conidia are not produced for dispersal through the air, but inside the plant. The fungus uses the xylem sap stream for distribution of conidia to the stem and leaves. Conidia formation is therefore an important step during host invasion [5]. The abilities of the Δ*VEL1-3* and Δ*VOS1* strains to produce spores in liquid simulated xylem medium were compared to wild type (Fig 4). Quantification revealed similar amounts of spores for *V*. *dahliae* Δ*VEL2* and Δ*VOS1* strains compared to wild type, suggesting that these velvet proteins are dispensable for this process.

In contrast, the spore numbers produced by the Δ*VEL1* strain declined by more than 80% compared to wild type and could be restored by reintroduction of the *VEL1* gene. The *VEL3* gene also contributes substantially to conidia formation with an approximately 60% reduction in numbers upon deletion of *VEL3*. Consistently, analysis of different *V*. *dahliae* double deletion strains revealed that conidia formation is also reduced in the Δ*VEL1*/Δ*VEL3* double mutant strain (Fig 4). These data suggest that *V*. *dahliae* Vel1 and Vel3 together support asexual conidiation. In contrast, Vel1 is required for the formation of microsclerotia and certain secondary metabolite production, whereas Vel3 acts negatively on these processes. It is yet elusive, whether Vel3 as homodimer or within a heterodimer has different regulatory functions during development.

### All *V*. *dahliae* velvet domain proteins are present during vegetative filamentous growth and can form three heterodimers

**Fig 5:**
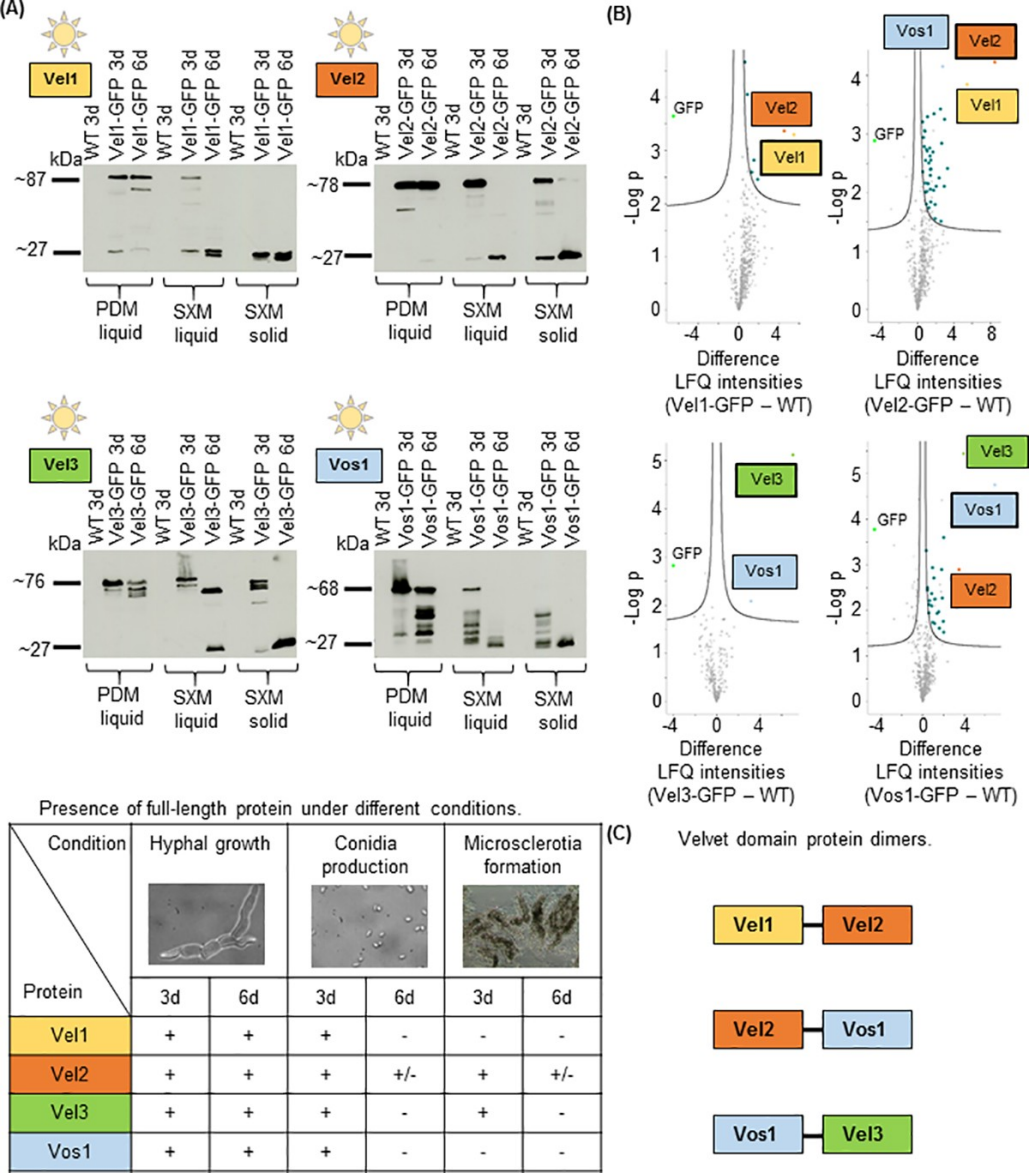
Velvet domain proteins during vegetative growth and development of *V*. *dahliae*. **(A)** Western hybridization with a GFP antibody for velvet domain proteins fused to GFP in extracts from *V*. *dahliae* strains of *VEL1*-*GFP*, *VEL2*-*GFP*, *VEL3*-*GFP* and *VOS1*-*GFP* grown during conditions stimulating filamentous growth (PDM: liquid potato dextrose medium), conidia (SXM: pectin-rich simulated xylem medium) or microsclerotia (SXM plates covered by a nylon membrane) formation. 1x10^6^ freshly harvested spores were inoculated and extracts were prepared after growth for three and six days at 25°C in light (free GFP: 27 kDa; Vel1-GFP: 87 kDa; Vel2-GFP: 78 kDa; Vel3-GFP 76 kDa; Vos1-GFP: 68 kDa). Presence (+) or absence (-) of full-length proteins during different conditions is summarized in the table below. **(B)** Vel1-3-GFP and Vos1-GFP interacting proteins during filamentous vegetative *V*. *dahliae* growth. Spores from the strains mentioned in (A) were grown in liquid PDM during constant light at 25°C for 72 h, subjected to GFP-trap-pull-down, trypsin digested and resulting peptides were analyzed by LC/MS. Volcano plots show differences of examined velvet-GFP fusion proteins in comparison to wild type (WT) on x-axis and -Log p values on y-axis. Displayed is the mean of three independent experiments. Significant hits of the pull-down are visible in the upper right part. Missing values were replaced four times with imputed values to obtain reliable interaction candidates. Proteins that were significant in all four repetitions are colored (Vel1: yellow, Vel2: orange, Vel3: green, Vos1: light blue, other proteins: dark blue). GFP is indicated in lime and the bait is framed by a black line. Vel1 interacts with six other proteins including Vel2; Vel2 interacts with Vel1 and Vos1 and 40 other proteins; Vel3 interacts with Vos1; Vos1 interacts with Vel2 and Vel3 as well as 21 other proteins. **(C)** Summary of the velvet domain dimer complexes identified during *V*. *dahliae* vegetative growth.

The presence of velvet proteins was analyzed during conditions, favoring hyphal growth (liquid glucose-rich PDM). Strains with functional *GFP* gene fusions integrated at the endogenous *VEL1-3* and *VOS1* loci driven by their native promoters were analyzed (S17B Fig). All four velvet proteins were present after three and six days of growth in PDM during illumination (Fig 5A).

Subcellular localization of the GFP-Velvet protein fusions, which were ectopically overexpressed, in a *V*. *dahliae* strain with histone H2B-RFP labeled nuclei revealed a predominantly nuclear localization of Vel1, Vel3 and Vos1. The majority of Vel2 localized to the nucleus, but smaller subpopulations were found in the cytoplasm (S21 Fig). The localization pattern did not change during incubation in darkness (S21A Fig). Overexpression of the *GFP-VELVET* fusions did not alter the colony phenotype of respective strains (S17C Fig). *V*. *dahliae* Vel1 and Vel2 can enter the nucleus independently from each other (S21B Fig), which is different to *A*. *nidulans* where VeA supports VelB nuclear entry [17]. Neither overexpression of *VEL2* in the Δ*VEL1* strain nor overexpression of *VEL1* in the Δ*VEL2* strain changed the phenotype of the respective deletion strain (S17D Fig).

Velvet proteins form various homo- and heterodimers in *A*. *nidulans* [28]. Protein pull downs were conducted to examine interaction partners of *V*. *dahliae* velvet proteins by mass spectrometry. Velvet proteins were endogenously fused to GFP and enriched from cell lysates through affinity purification. Protein samples were digested, peptides were analyzed with LC/MS for protein identification and label-free relative quantification. Several significant interaction partners were identified for Vel1 (six proteins), Vel2 (42 proteins) and Vos1 (23 proteins), whereas Vos1 was the only significant interactor found for Vel3. Interactions for Vel1-Vel2 and Vel2-Vos1 heterodimers were observed (Fig 5B and 5C, and S1–S8Tables). Vel1 interaction partners included proteins involved in redox and protein metabolism. Many Vel2 interaction partners are connected to different metabolic processes. Furthermore, Vos1 interacting proteins are often involved in energy metabolism and transport. Under the tested conditions, no significant interaction could be identified between the velvet proteins with any epigenetic methyltransferase as Lae1, which is described as VelA-VelB interacting protein in other fungi [17]. Vos1, which is required for viability of spores in *A*. *nidulans* [16], is part of two *V*. *dahliae* heterodimers. Their function in the plant pathogen is yet elusive, because the Δ*VOS1* strain did not reveal any obvious differences compared to wild type under the tested conditions (S8 Fig).

### All *V*. *dahliae* velvet domain proteins are present during early conidia production, whereas only Vel2 and Vel3 are present during early microsclerotia formation in light

Germination of fungal spores on appropriate substrates results in vegetative hyphae, which are in several filamentous ascomycetes initially incompetent to react to environmental triggers [46,47]. Only when developmental competence is established, the fungus can receive and respond to environmental signals as light with substantial shifts in differential gene expression [48]. *V*. *dahliae* strains with functional *VEL1-3/VOS1-GFP* gene fusions were monitored during conditions favoring conidiation (liquid pectin-rich simulated xylem medium, SXM) and microsclerotia formation (solid SXM medium) after three and six days of growth during illumination (Fig 5A). When conidia formation is favored, GFP-fusions of all velvet proteins were present after three days in the early stage but were destabilized after six days. During degradation of the fusion proteins, signals migrating at lower molecular weight were observed as well as an increase in signals at around 27 kDa, which presumably derive from free GFP.

Microsclerotia formation in light is promoted by Vel2 and inhibited by Vel3 (Fig 2B and 2C). On solid medium favoring microsclerotia development Vel2-GFP and Vel3-GFP are the only velvet proteins, which were identified after three days, but are destabilized after six days of cultivation (Fig 5A). Vel2 increases microsclerotia production specifically in light. Consistently, Vel2-GFP is more stable in light than in darkness (SXM solid, S22 Fig). Asexual sporulation happened independently of Vel2 and revealed a different stability pattern. During conidiation, Vel2-GFP is destabilized in light, but more stable in darkness (SXM liquid, S22 Fig). During filamentous growth Vel2-GFP is stable in light as well as in darkness (liquid PDM, S22 Fig). Again, signals of potential degradation products as well as of free GFP were observed.

This suggests that *V*. *dahliae* possesses a control system, which increases cellular Vel2 steady state levels during microsclerotia formation in light but decreases them in darkness. Such a control system could be due to differential gene expression or protein turnover control for the light-dependent developmental regulator of microsclerotia formation.

### The Vel1 protein induces disease symptoms in *V*. *dahliae* infected tomato plants

The requirement of Vel1 and Vel3 for efficient conidiation suggests a potential impact of velvet proteins on *V*. *dahliae* caused disease of a host. Both proteins are present during hyphal growth and early conidia formation, which is required for fungal transport within the plant vascular system. Vel1 proteins of a limited number of other fungal plant pathogens have already been connected to virulence, but the exact molecular mechanisms, which cause disease are yet elusive [24,49,50]. Plant symptoms caused by wild type and corresponding mutant strains were compared. Roots of tomato seedlings were wounded and inoculated with fungal spores by root dipping. Water treated plants served as control and resulted in a majority of plants categorized as healthy (Mock). Disease symptoms of all fungal strains were assessed and calculated into a disease score. Within this score, the plants were classified as healthy or with only weak symptoms corresponding to the control, or with strong and even heavy disease symptoms.

**Fig 6:**
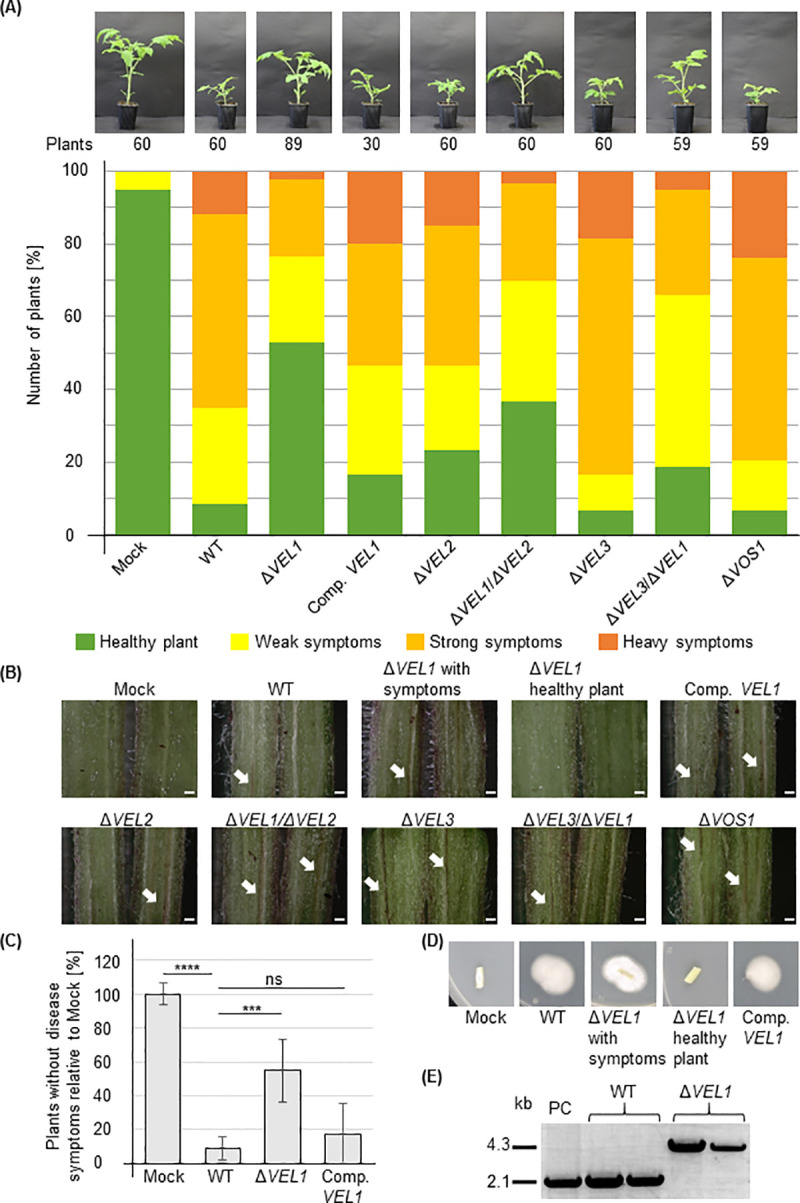
*V*. *dahliae* requires Vel1 to induce disease symptoms in tomato plants. Tomato plants were grown for 10 days and inoculated by root dipping with the same amount of fungal spores of *V*. *dahliae* wild type and indicated mutant strains defective in genes for velvet domain proteins or a *VEL1* complementation strain. The mock control was treated with demineralized water. Single experiments included at least 14 plants and two different transformants for each deletion and double deletion strain were used. Each strain was analyzed in at least two independent experiments. **(A)** Disease symptoms were scored after 21 days (16 h, 25°C: 8 h, 22°C, light: dark). Measurements included heights of plants until the vegetation point, length of the biggest leaf and fresh weight of aerial plant parts. Parameters were calculated into a disease score leading to the categories healthy plant, weak symptoms, strong symptoms and heavy symptoms. The diagram shows the relative number of plants of each category and a representative plant for each strain. Mock treated plants were mostly healthy with some plants with weak symptoms. More than 60% of plants inoculated with wild type developed heavy or strong disease symptoms. **(B)** Images of hypocotyl cross sections with arrows indicating discolorations, which are not found in uninfected plants and in approximately 40% of plants treated with the Δ*VEL1* strain. Scale bar = 500 μm. **(C)** Quantification of plants without disease symptoms. Means and error bars represent the standard deviation and mean of two independent experiments including at least 14 plants and at least two deletion strains. Significant differences calculated by t-test are indicated: ***:p<0.001; ****:p = 0.0001; ns: not significant. **(D)** Fungal growth from stem sections of tomato plants. Stems of tomato plants treated with indicated *V*. *dahliae* strains were surface sterilized and incubated for seven days at 25°C on PDM plates supplemented with chloramphenicol (34 μg/ml). Wild type as well as *VEL1* complementation strain could be re-isolated from the stem. The *VEL1* deletion strain could only be re-isolated from plants displaying disease symptoms. **(E)** Verification of isolated fungal strains by PCR. Fungal material isolated from plate was inoculated in PDM for mycelia production. Spores of wild type were inoculated in PDM as positive control (PC). Genomic DNA was extracted and PCR was conducted to verify the wild type (WT: 2130 bp) or *VEL1* deletion (Δ*VEL1*: 4342 bp) genotype.

When treated with wild type, less than 10% of the plants were classified as healthy and additional approximately 25% showed only weak symptoms. In contrast, most plants (approximately 65%) suffered from strong or heavy symptoms induced by the fungal pathogen (Fig 6A). Δ*VOS1* and Δ*VEL3* strains resulted in even more than 65% plants with strong or heavy symptoms. Vos1 and Vel3 are therefore not required for disease induction under the tested conditions, although Vel3 contributes to conidiation as important transport vehicle of the fungus within the xylem of the host. Infection with the Δ*VEL2* strain also exhibited more than 50% plants with strong or heavy symptoms, which does not support an essential function of Vel2 for fungal virulence either.

In contrast, infection of tomato plants with the Δ*VEL1* strain resulted in almost 80% plants, which are either healthy (more than 50%) or show only weak symptoms and hardly any plants with heavy symptoms. This effect could be restored in a strain where the *VEL1* gene was reintroduced. Plant treatment with double deletion strains as Δ*VEL1*/Δ*VEL2* or Δ*VEL1*/Δ*VEL3* resulted in reduced disease severity compared to wild type infected plants with approximately 30% of plants displaying strong or heavy symptoms. This suggests that the *VEL1* gene plays a significant role in the *V*. *dahliae* caused disease in tomato.

As additional indicator of disease or plant immune response, hypocotyl cross sections of the plants were investigated for discoloration caused by fungal infection (Fig 6B). In the hypocotyl from healthy plants no discoloration was observed. Plants with symptoms infected by the wild type or complementation strains exhibited brownish discoloration. The same was found following Δ*VEL2*, Δ*VEL3* or Δ*VOS1* strain infections. Whereas 97% of wild type treated plants showed strong discolorations of the hypocotyl only approximately 60% of plants inoculated with *VEL1* deletion strains had changes in hypocotyl color, which mostly were less severe.

Vel1 is required for efficient *V*. *dahliae* spore formation. The infection rate between wild type and the Δ*VEL1* strain was compared to evaluate whether there are already differences in the potential to infect plants after inoculation with the same number of conidia. The amount of plants without disease symptoms relative to wild type was determined (Fig 6C). Whereas the wild type fungus infected significantly more than 80% of all plants, only about half of the plants could be infected by the Δ*VEL1* strain (Fig 6C). This suggests that the *VEL1* deletion strain does not only produce less conidia than wild type but also spores with a lower potential to infect a host.

The fungal presence in the plant was analyzed by incubation of tomato stem intersections on plates for re-isolation of fungal material. No fungus grew from symptomless plants treated with the Δ*VEL1* strain. However, wild type and the Δ*VEL1* strain could be re-isolated from plants showing disease symptoms (Fig 6D). This was verified by PCR using DNA from the isolated fungus as template (Fig 6E). These data suggest that the *V*. *dahliae VEL1* regulatory gene, which is required for microsclerotia and conidia formation and the control of secondary metabolism, represents a velvet protein with a substantial role in fungal virulence in a plant host.

### Vel1 is required for initial steps in plant root colonization by *V*. *dahliae*

Initial steps of *V*. *dahliae* plant infections include colonization of the host root and subsequent penetration at suitable sites with the help of hyphopodia as swollen hyphal tips. The Vel1 protein is present during hyphal growth of *V*. *dahliae* (Fig 5A) and is the only velvet domain protein required for disease symptom induction in tomato plants (Fig 6). Therefore, we focused on the role of Vel1 in the infection process and elucidated its role in root colonization of the vascular plant pathogen. *A*. *thaliana* roots were examined for initial fungal colonization and entry. Roots were transferred into a spore solution of wild type or the Δ*VEL1* strain, which both ectopically overexpressed *GFP*. Expression of *GFP* did not change the phenotype of the respective strains (S17E Fig).

**Fig 7:**
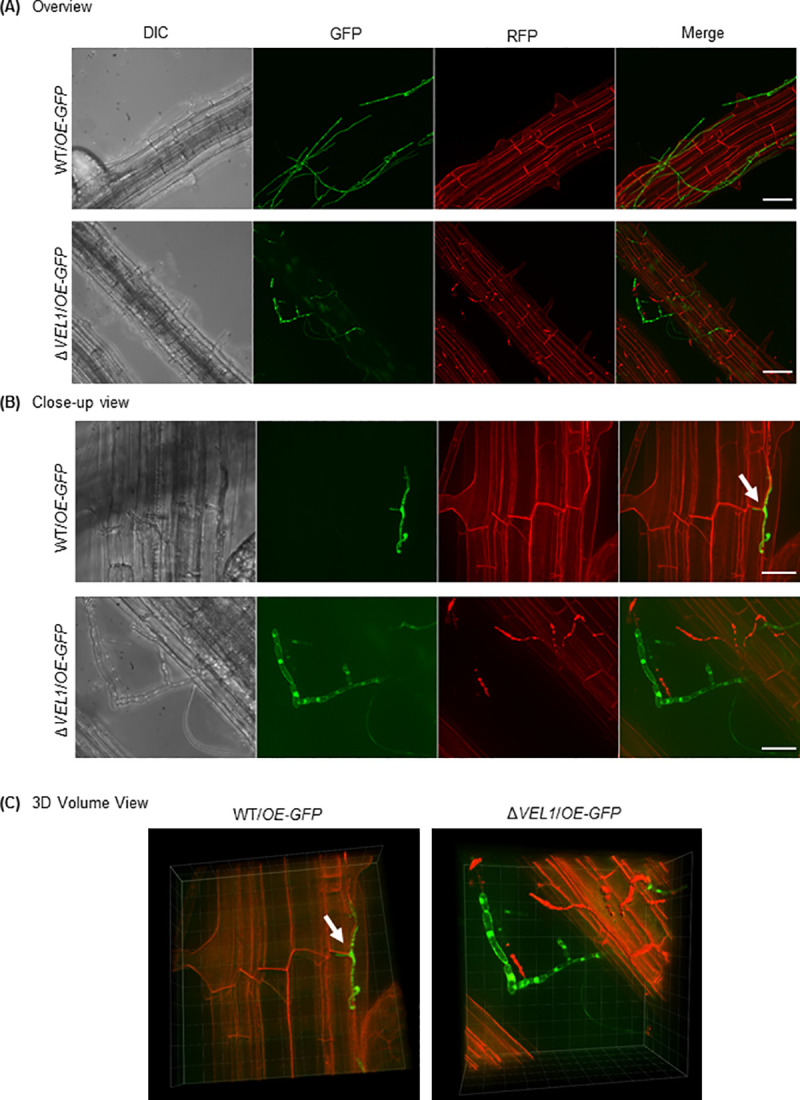
Vel1 is required for colonization and penetration of *Arabidopsis thaliana* roots. Fluorescence microscopy images of wild type ectopically overexpressing green fluorescent protein (GFP, WT/*OE-GFP*) and the Δ*VEL1* strain ectopically overexpressing GFP (Δ*VEL1*/*OE-GFP*). Surface sterilized *A*. *thaliana* seeds were grown for three weeks during long-day conditions (16 h, 25°C: 8 h, 22°C, light: dark) on Murashige and Skoog Medium. After inoculation with fungal spores by root dipping the plants were further incubated for five days on 1% agarose. *A*. *thaliana* roots were stained with propidium iodide solution for microscopy (0.0025% propidium iodide, 0.005% silwet). Differential interference contrast (DIC), green fluorescent filter view (GFP), red fluorescent filter view (RFP) and a merge of GFP and RFP channels are presented. White arrows indicate potential fungal penetration points on the root surface, which are absent in the Δ*VEL1* strain. **(A)** Overview projections of stacks of single images of wild type and Δ*VEL1* strains, both overexpressing *GFP*; scale bar = 50 μm. **(B)** Close-up view of the same strains as in (A); scale bar = 20 μm. **(C)** 3D volume view of single picture stacks displaying the same position as in (B).

Roots were examined after five days by fluorescence microscopy. The wild type was able to colonize the root surface (Fig 7A). A more detailed view exhibited penetration points with swollen hyphae indicating hyphopodia formation (Fig 7B, white arrow). Generation of 3D volume views from 2D pictures showed the entry into the root for the wild type (Fig 7C, white arrow). Roots infected with the Δ*VEL1* strain showed less colonization than wild type (Fig 7A). In addition, propidium iodide staining indicated substantial amounts of dead hyphae, which are not present in wild type treated plants (Fig 7B). Wild type-like penetration points were not visible. In the 3D view, hyphae were detected, which are vacuolized and swollen suggesting fitness problems of the fungus.

This study provides new insights into the first contact of the fungus with the plant root. *V*. *dahliae* Vel1 is not only required for spore-dependent transport of the fungus in the plant xylem system, but necessary for the first phases of plant colonization including the potential to form hyphae, which are viable during fungal-root interaction. Vel1 functions include the fungal potential to penetrate plant barriers with the help of hyphopodia as prerequisite for successful entry into the vascular xylem system and virulence.

## Discussion

**Fig 8:**
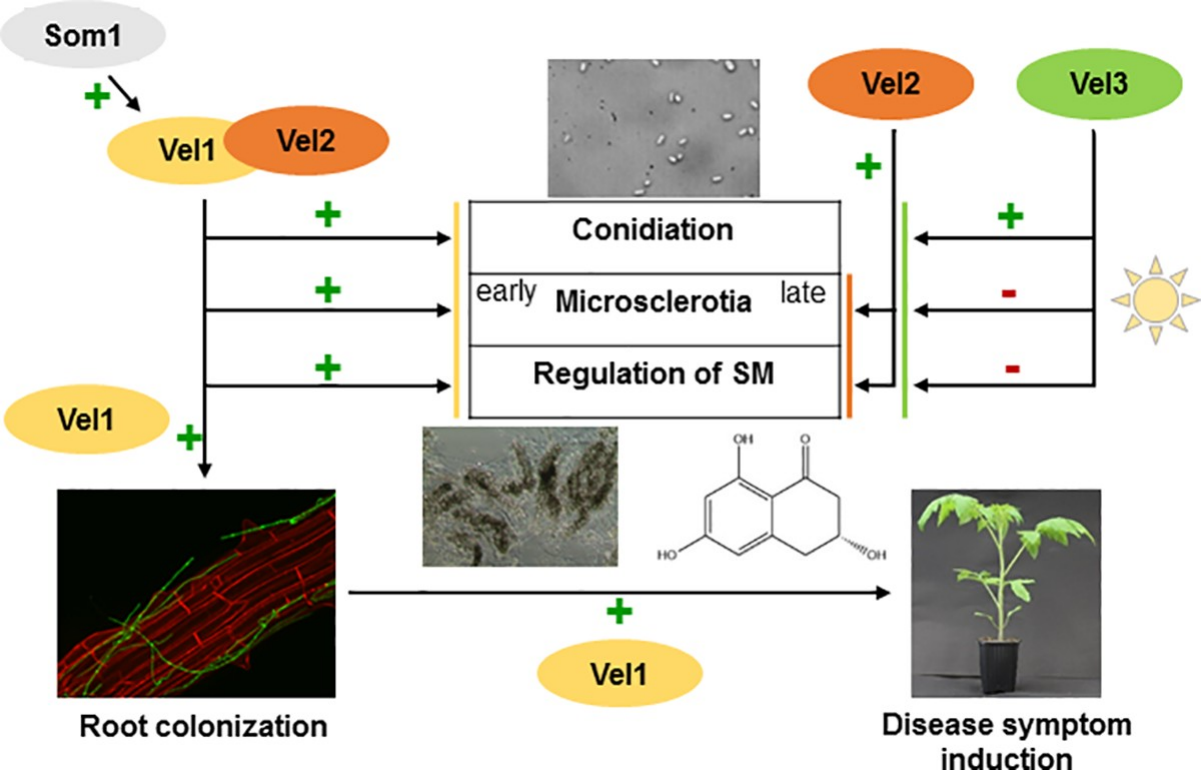
*V*. *dahliae* velvet protein functions in development, plant interaction and disease. Vel1 (yellow) forms a heterodimer with Vel2 (orange) during vegetative growth and activates *V*. *dahliae* conidiation, microsclerotia formation and a specific secondary metabolism (SM). Vel1 is required for root colonization, penetration and propagation within the host, which requires conidia formation. Som1 (grey) might activate Vel1. Vel2 contributes to a specific SM and is also present during microsclerotia formation when Vel1 is already absent and supports resting structure formation in light. Vel3 (green) reduces Vel1 activated SM as well as microsclerotia formation in light, but activates conidiation (+ indicates supportive functions,—reducing functions).

The major finding of this comprehensive study on the velvet domain proteins and their respective proteins is the identification of the regulatory protein Vel1 as new promising target to control the vascular plant pathogenic *V*. *dahliae* life cycle, the interaction with host plants and induction of disease. Vel1 has a remarkable novel impact on initial plant root colonization and entry and causes disease symptom induction *in planta*. Conidia formation for colonization of the xylem requires the presence of Vel1 and Vel3. Synthesis of melanin precursors and other secondary metabolites is promoted by Vel1 and Vel2 but hindered by Vel3. Formation of melanized resting structures requires the presence of Vel1 and light dependent Vel2 as positive regulators and Vel3 as negative modulator. The changes in scytalone production might also be directly associated with the phenotypical impairments of the respective strains in resting structure production. Accordingly, most developmental processes of this fungus require the interplay of several velvet proteins (Fig 8). Velvet domain proteins as Vel2 and Vel1 can form different homo- and heterodimers [10]. Protein stability control seems to be an important regulatory feature for controlling dimer formation, which has not yet been studied for most velvet domain proteins, but includes for the Vel1 *A*. *nidulans* counterpart a complex interplay between ubiquitination and deubiquitination [30]. Presumably, rather Vel1-Vel1 than Vel1-Vel2 is primarily required to mediate Vel1- dependent *V*. *dahliae* pathogenicity on tomato. Stability control of velvet domain proteins might be an important additional regulatory level, which can change the ratio between different velvet heterodimers considerably. A single deletion strain might therefore simultaneously affect several of the velvet domain homo- or heterodimer transcription factors, because absence of one interaction partner might lead to an imbalance of these complexes. This might explain the observed complex deletion phenotypes of single velvet protein encoding genes in different fungi, which are relevant for development, secondary metabolism or virulence.

Velvet proteins can link fungal development to plant pathogenicity. In the apple pathogen *Valsa mali*, VeA and VelB negatively regulate conidiation. VeA and to a smaller extent also VelB reduce virulence [50]. In *M*. *oryzae VEA*, *VELB* and *VELC* positively contribute to developmental processes as conidiation [51]. Only *VEA* and *VELC* deletion strains reduced disease development. *VEA* deletion strains displayed defects in appressorium formation, whereas *VELC* deletion strains developed normal appressoria but failed to penetrate plant cells [51]. *VOSA* seems to be dispensable for *M*. *oryzae* development and pathogenicity [51]. *V*. *dahliae* Vel1 and Vos1 have similar roles as the *M*. *oryzae* proteins. *B*. *cinerea* VEL1 positively regulates expression of secondary metabolite-related genes as *pks12* for melanogenesis [49,52]. The *M*. *oryzae* melanin pigment mutants *AP1* and *MET13* are less virulent [53,54], whereas melanin is dispensable for *B*. *cinerea* virulence [55]. The *V*. *dahliae* Δ*VEL2* strain is hindered in microsclerotia formation but induces similar disease symptoms as wild type, suggesting that melanized microsclerotia production and virulence are independent. Consistently, *V*. *dahliae PKS1*, *CMR1* or *VTA1* deletion strains produce colorless microsclerotia and induce wild type-like disease symptoms [38,56]. The transcription factor Cmr1 is directly involved in the regulation of melanin biosynthesis genes [38] and we could show that the expression of the *CMR1* gene is reduced in a *VEL1* deletion strain. This is in accordance with the absence of microsclerotia production in the Δ*VEL1* strain.

*V*. *dahliae* enters its host through the root surfaces and later colonizes the whole plant [1,2]. Prerequisites are host sensing and microsclerotia germination in the soil followed by directed fungal hyphal growth to reach the rhizosphere of a susceptible plant root. The fungus senses the plant environment and adapts its secretion response [57]. All velvet proteins are detectable as different dimers in the cell during vegetative growth. Fungal root colonization is the first step for successful infection. Vel1 promotes the formation of hyphopodia for entering and reaching the vascular tissue. Root colonization requires fungal adhesion, which is controlled by different Verticillium transcription activators of adhesion (Vta) originally identified in a genetic screen with non-adhesive yeast strains [58,59]. Root infection studies revealed that *V*. *dahliae VTA2* and *VTA3* are important for successful colonization [58,59]. Vta1 is required to produce melanized microsclerotia, but dispensable for plant infection and root colonization [56]. *V*. *dahliae SOM1* and its counterpart of the human opportunistic pathogen *Aspergillus fumigatus* are *VTA*-like genes, required for fungal adherence and virulence of a plant and a human pathogen, respectively [58,60]. *V*. *dahliae* Som1 is required for plant root penetration and proliferation and controls the transcription of the *VEL1* gene at the molecular level. This supports a temporal connection between the Som1 and the Vel1 controlled transcriptional networks to proceed from adhesion to plant penetration and colonization. Velvet mutant strains of *B*. *cinerea* were impaired in the acidification of host tissue and showed alterations in gene expression. These defects in acidification were connected to a reduction in lesion formation on the host [61]. The root colonizing fungus *Fusarium oxysporum* induces root alkalization to infect tomato plants [62]. The role of pH alterations in *V*. *dahliae* root infection or following symptom induction and a regulatory involvement of velvet proteins remains elusive.

Light regulates developmental processes, metabolic pathways and location of proteins in the fungal cell [10,63,64]. Velvet proteins control the life cycle of the saprophytic soil fungus *A*. *nidulans* in a light-dependent manner. Light promotes asexual spore formation, whereas sexual development with the production of cleistothecia and the appropriate protective secondary metabolism is promoted in darkness by VeA and VelB as light-dependent factors [10,17,48]. These closed fruiting bodies are overwintering structures similar to microsclerotia, except that their formation by *V*. *dahliae* is not linked to meiosis and a sexual program [65]. VelC supports sexual development, but also controls genes for asexual development [25]. VosA regulates spore viability, trehalose biosynthesis and controls genes for secondary metabolism [16,26].

Light perception of *Verticillium spp*. is largely unknown but includes genes for a circadian system [66]. Discriminating between light and dark might be a way to ascertain the fungal position in soil, to start colonization at roots or to grow *in planta* into the xylem and upper parts of the host. Vel1, which carries a PEST sequence and is less stable than Vel2, controls earlier developmental processes. PEST motifs are present in many short-lived proteins as e.g. in the Hac1 transcription factor of the unfolded protein response of *S*. *cerevisiae* [67]. Hac1 is degraded by the 26S proteasome pathway and the protein can be stabilized upon deletion of the PEST coding sequence of the corresponding yeast gene [68]. Studies with the yeast uracil permease Fur4 suggest a link of stability to phosphorylation within the PEST/PEST-like motifs [68]. In *A*. *nidulans* ubiquitin-dependent VeA degradation occurs also during early development and is regulated by the UspA deubiquitinase enzyme [30]. Vel1 might destabilize Vel2 in a heterodimer as a mechanism, which enables the fungus to adapt to different light conditions. *V*. *dahliae* Vel2 function is presumably light-dependent because incubation of the Δ*VEL2* strain in changing light conditions results in differences in microsclerotia production. The Vel2 protein is more stable in light than in darkness during microsclerotia formation. Similar to Vel1, *V*. *dahliae* Vel3 presumably functions in early developmental steps. Vel3 also carries a PEST instability region and is destabilized during further development. The Vel3 function in microsclerotia formation is reminiscent to the *A*. *nidulans* counterpart VelC, which is required during the early phase of fruiting body formation [25].

The velvet domain enables homo- and heterodimer formation for controlling developmental processes and secondary metabolite production [10,28]. *A*. *nidulans* VosA binds to many promoter sequences and is required for spore viability, survival and correct secondary metabolism of ascospores [16,26,27]. Protein interaction studies revealed a connection of *V*. *dahliae* Vos1 to 23 proteins, including Vel2 and Vel3, but cellular functions of Vos1 remain yet elusive. In addition to the VeA-VelB, VelB-VosA and VelC-VosA DNA binding heterodimers, *A*. *nidulans* velvet proteins also interact with epigenetic methytransferases including LaeA or VapB-VipC [17,28,69]. *V*. *dahliae* Vel1-Vel2, Vel2-Vos1 and Vel3-Vos1 heterodimers could be verified, but interactions to putative methyltransferases are yet missing and it is unclear whether they exist during specific growth conditions. Especially, the formation of the two heterodimers Vel2-Vel1 and Vel2-Vos1 is a common feature, which is conserved between *A*. *nidulans*, *N*. *crassa* and *V*. *dahliae* [17,22,28], whereas other interactions might vary between different fungi or different cultivation conditions. Vel2 is part of two heterodimers, which suggests that the control of the ratio between the distribution of Vel2 to either Vel2-Vel1 or Vel2-Vos1 might be an important conserved fungal control mechanism for development, pathogenesis and secondary metabolite formation.

*VEL1* and *VEL2* deletion strains exhibit the same phenotype regarding microsclerotia formation and show similarities in secondary metabolism, which further supports a common function of a Vel1-Vel2 heterodimer. Other *V*. *dahliae* Vel1 and Vel2 interaction partners include HAM13 (VDAG_JR2_Chr5g09190a), which counterpart is phosphorylated in the *N*. *crassa* MAP kinase signaling pathway and important for fungal communication [70]. It remains to be shown, whether a corresponding complex consisting of this protein together with Vel1 and Vel2 coordinates developmental processes, interaction to host cells and signaling in *V*. *dahliae*.

Among the Aspergilli, *A*. *niger* VeA is involved in spore production and dispersion [71]. In addition to their function in microsclerotia formation, *V*. *dahliae* Vel1 together with Vel3 promote fungal conidiation, which facilitates colonization of the upper parts of host plants. A *V*. *dahliae* Δ*VEL1* strain is impaired in colonization and entry into host roots. The application of wounded tomato plants allowed to circumvent this initial infection step, because a Δ*VEL1* strain could enter and grow within the host. Symptom induction in plants treated with this strain was decreased in comparison to wild type, presumably due to the reduced potential of conidia production and propagation within the plant vascular system. Lack of Vel3 reduces conidiation less than the lack of Vel1. Spore numbers produced by the Δ*VEL3* strain seem to be sufficient to effectively colonize plants, because symptom induction in tomatoes was comparable to wild type when this strain was used for inoculation.

The fungus remains in the plant after successful infection until the host dies and forms microsclerotia as resting structures. Formation of microsclerotia means a readjustment of developmental programs. Vel1 and Vel2 are positive regulators of microsclerotia formation, whereas Vel3 is a negative regulator. Except of its promoting role in conidiation, Vel3 acts antagonistically towards Vel1 and Vel2. Velvet genes regulate secondary metabolite expression in different fungi [13–15,17,24,49,64]. Microsclerotia contain the pigment melanin, a secondary metabolite, which enables the fungus to overcome difficult environmental conditions [38,72,73]. Secondary metabolite analyses of velvet deletion strains indicate that Vel1, Vel2 and Vel3 regulate the formation of the melanin precursor scytalone. This result corroborates the phenotype on plate as *VEL1* and *VEL2* deletion strains are hindered in microsclerotia production and the Δ*VEL3* strain increases melanization compared to wild type. Another metabolite controlled by Vel1 and Vel2 is an intermediate in 6-methoxymellein production, which might harm plant cells similar to terrein of *A*. *terrreus* [41]. The exact function of these fungal metabolites in the interaction with plants or other organisms remains to be explored.

In summary, we showed that Vel1 of a vascular plant pathogen is a key factor for early plant colonization. Vel1 controls fungal development including asexual spore and microsclerotia formation linked to an appropriate secondary metabolism and symptom induction and disease. Other *V*. *dahliae* velvet proteins, except Vos1, which exhibits yet unknown cellular functions, contribute to these developmental processes and the linked secondary metabolite production, but are not as omnipresent as Vel1. The broad involvement of velvet domain proteins in the fungal life cycle leads to pleiotropic effects. This complex integration of velvet proteins makes especially Vel1 an interesting target to combat the growing threat of Verticillium wilt in our crops.

## Materials and methods

*V*. *dahliae*, *Agrobacterium tumefaciens* and *Escherichia coli* strains used are listed in S9 Table. Primers are listed in S10 Table, plasmids in S11 Table. Additional data can be found in the S1 Data file.

### Bioinformatic methods

Annotations were used according to the Ensembl Fungi database [74]. Sequence analysis of proteins was carried out by using the InterPro website [75,76]. Nuclear localization signals (NLS) were predicted by using cNLSmapper [77] with default settings. PEST sequence prediction was conducted using epestfind (http://emboss.bioinformatics.nl/cgi-bin/emboss/epestfind) with default settings. The sequences of other fungi mentioned in this study were obtained from the Ensembl Fungi database [74]. Multiple sequence alignments of different fungi were performed by MegAlignPro (DNASTAR, version 12.1.0) using ClustalW sequence alignment. Structural formulas were created using ChemDraw Professional 16.0 (PerkinElmer).

### Cultivation conditions and media

*E*. *coli* and *A*. *tumefaciens* cells were cultivated in lysogeny broth medium [78] supplemented with 100 μg/ml kanamycin (AppliChem) or 100 μg/ml ampicillin (Carl Roth) if needed. Fungal strains were incubated as described previously [56]. Media were supplemented with 72 μg/ml nourseothricin (Werner BioAgents), 50 μg/ml hygromycin B (Invivogen), 50 μg/ml geneticin G418 (Sigma-Aldrich) and 300 μg/ml cefotaxime (Fujifilm Wako chemicals) if required.

### Plasmid and strain constructions

For construction of deletion strains, gene open reading frames were replaced by homologous recombination with a resistance cassette. For complementations, the genes were reintroduced into the deletion strains at the genomic locus. All genes were fused to *GFP* at the 3’ end and integrated endogenously. In addition, the *GFP*-fused genes were expressed ectopically under the strong *A*. *nidulans gpdA* promoter for localization studies. Further details can be found in the supporting information (S1 Text).

### Transformation techniques

Chemically competent *E*. *coli* cells were transformed by the heat shock method as described before [79,80]. Competent *A*. *tumefaciens* cells were transformed by the freeze-thaw method as described by Jyothishwaran *et al*. [81]. *V*. *dahliae* was genetically manipulated as described by Bui *et al*. [58] using *Agrobacterium tumefaciens*-mediated transformation.

### Southern hybridization

For mycelium production strains were grown in PDM at 25°C. Mycelia were harvested using Miracloth filters (Calbiochem Merck) and ground in liquid nitrogen. Genomic DNA was extracted using phenol. Southern hybridization was carried out using the Amersham AlkPhos Direct Labelling and CDP-Star Detection reagents (GE Healthcare). Further details can be found in previous publications [56,58,59].

### RNA extraction and cDNA synthesis

RNA was extracted using the following protocol. Fungal strains were grown in triplicates in PDM for extraction of RNA. Approximately 0.5 ml ground mycelium was mixed with 1.4 ml TRIzol (Invitrogen) for 5 min. After adding 250 μl chloroform the solution was mixed again for 10 min. Centrifugation for 30 min at 4°C led to the formation of two phases. The upper phase containing the RNA was transferred into a fresh reaction tube and mixed with 400 μl isopropanol and 400 μl high-salt buffer (1.2 M NaCl, 0.8 M Trinatriumcitrate). After inverting the reaction tube several times and centrifugation for 15 min at 4°C, the supernatant was discarded. The RNA was washed with 250 μl 70% ethanol, centrifuged again for 10 min at 4°C and the supernatant was removed. Afterwards the RNA was dried at RT and dissolved in 100 μl RNase-free water at 65°C. Alternatively, RNA was extracted from fungal mycelium using the Direct-zol RNA MiniPrep Kit (Zymo Research). cDNA synthesis was carried out with the QuantiTect Reverse Transcription Kit (Qiagen) using 0.8 μg of RNA. The absence of genomic DNA in the cDNA was tested with the primers SZ19 and SZ20, binding in the *H2A* gene.

### Verification of gene annotation

Annotations from the Ensembl Fungi database [74] were verified by cDNA amplification from wild type JR2 [82] and subsequent sequencing. To verify the annotation of *VEL1* the primers AO11 and AO12 were used. The annotation of *VEL2* was confirmed by VelB-F1 and VelB-R1. *VEL3* annotation was confirmed by using the primers AO3 and AO13. The *VOS1* annotation was verified by using Vos1orf-E1 and Vos1orf-E2.

Ensembl Fungi annotations for *VEL1*, *VEL2* and *VEL3* could be confirmed. The correct annotation of *VOS1* starts already 528 base pairs upstream from the start codon predicted by Ensembl Fungi as confirmed by amplification and sequencing of cDNA as well as analysis of the peptide sequence obtained by LC/MS analysis (S4 and S5E Figs).

### Quantification of gene expression

The expression levels of the transcription factor Cmr1 encoding gene were quantified relative to the reference genes histone *H2A* (primers SZ9 and SZ10) and *EIF2B* (primers SZ11 and SZ12) by qRT PCR using the primers SB40 and SB41 as described before [83] using Mesa Green qPCR MasterMix Plus for SYBR Assay (Eurogentec). Significances were calculated by t-test with the SISA online tool [84]. Significances indicate: *:p<0.05.

### Phenotypical analysis

Phenotypical analysis was conducted on SXM and CDM as described before [56] using 5x10^4^ freshly harvested spores of each strain. Plates were incubated for 10 to 14 days upside down and examined by binocular and light microscopy for microsclerotia formation. Plates were incubated either in constant light or constant darkness at 25°C or during long day conditions (16 h, 25°C: 8 h, 22°C, light: dark) in a growth chamber.

### Quantification of microsclerotia formation

The melanization of fungal colonies was quantified as described before [83]. 5x10^4^ freshly harvested spores were inoculated in triplicates on 30 ml CDM plates and incubated at 25°C during constant illumination. After 10 days, aerial mycelium was removed, and pictures were taken from the top view. Pictures were converted to 8-bit grey scale. The brightness factor of the colony centres was measured using the ROI Manager of the ImageJ software [85]. The brightness factor of the medium was used for background normalization. Mean values of three colonies were considered as one biological replicate (n = 1). Statistical significances were calculated by t-test with the SISA online tool [84]. Significances indicate: *:p<0.05; ***:p<0.001.

### Quantification of conidiospores

For quantification of conidia production, 2x10^5^ freshly harvested spores were inoculated in 50 ml SXM and incubated at 25°C under constant agitation for seven days. Each strain was inoculated in triplicates. After seven days, the spores were filtered through Miracloth filters (Calbiochem Merck) and centrifuged. Spore sediments were dissolved in equal amounts of sterile water. By using the Coulter Z2 Particle Counter (Beckman Coulter), the conidiospore concentration was determined. The conidia formation was quantified relative to the wild type. Each experiment was performed with three technical replicates (n = 1). Bars represent the mean values of all experiments and error bars correspond to standard deviations. Statistical significances were calculated by t-test with the SISA online tool [84]. Significances indicate: **:p<0.01; ***:p<0.001; ****:p<0.0001; ns, not significant.

### Fluorescence microscopy and localization studies

The velvet proteins were tested for their localization by gene overexpression and C-terminal fusion with *GFP*. To visualize the nucleus, the constructs were additionally transformed with a Histone H2B-RFP overexpressing strain. 1x10^4^ freshly harvested fungal spores were inoculated in 300 μl PDM in 15 μ-Slide 8 Well microscopy chambers (ibidi) and incubated at 25°C for 16h either in light or darkness. Subcellular localization of the proteins was examined by fluorescence microscopy (100x/1.4 oil objective). Exposure time for GFP was 500 ms and for RFP 800 ms.

### Arabidopsis root infection

*Arabidopsis thaliana* Col-0 was used for root infection studies with the indicated *V*. *dahliae* strains as described previously [56,58]. Seeds were surface sterilized and grown on Murashige and Skoog medium ([86], modified as described in [56]) overnight at 4°C and afterwards for 21 days under long day conditions (16 hours light at 25°C, 8 hours darkness at 22°C) in a climate chamber. One day prior to infection, the plants were transferred to 1% water agarose plates. Infection was conducted by root dipping in a spore solution with 1x10^5^ spores/ml for 35 min. The plants were further incubated on water agarose for five days in the climate chamber. For preparation of fluorescent microscopy, the roots were stained with 0.0025% (v/v) propidium iodide and 0.005% (v/v) silwet for 5 min in darkness. Experiments were conducted with two independent transformants of Δ*VEL1*, expressing free GFP (VGB443 and VGB444).

### Tomato plant infection

*Solanum lycopersicum* seedlings (“Money maker”, 10 days old) were used for infection experiments. Roots were slightly wounded by rubbing and infected by root dipping into a spore suspension of 1x10^7^ spores/ml for 40 min under constant agitation at ~30 rpm. Per strain 15 plants were used. In addition, 3 ml of the spore suspension (concentration 1x10^7^ spores/ml) was added to the soil of each pot. Plants were incubated for 21 days under long day conditions (16 hours light at 25°C, 8 hours darkness at 22°C). Further details on plant infection and evaluation of disease symptoms can be found in previous publications [56,57].

Presence of fungal material of the wild type or the *VEL1* deletion strain was verified by PCR using the primers AO24 and AO25, which bind in the 5’ or 3’ flanking region of the gene, respectively.

### Metabolite extraction and LC/MS analysis

Per strain, two plates of 30 ml CDM with glucose as carbon source were inoculated with 1x10^6^ freshly harvested spores. The plates were incubated at 25°C in light or darkness for two weeks. Secondary metabolite extraction was modified [87]. The homogenized fungal material was mixed with 5 ml H_2_O (LC/MS grade, Merck) and 5 ml ethyl acetate (LC/MS grade, Carl Roth) and incubated overnight during constant agitation. The samples were centrifuged for 10 min at 2500 rpm at 4°C. The upper phase was transferred into a vial and vaporized. The metabolites were dissolved in acetonitrile and H_2_O (LC/MS grade, Merck) (1:1), centrifuged at 8°C, 13000 rpm, 15 min and transferred into LC/MS vials. The samples were analysed with a Q Exactive Focus Orbitrap mass spectrometer coupled with an UltiMate 3000 high-performance liquid chromatography (HPLC) and a CAD-3000 detector (Thermo Fisher Scientific) as descried before [87]. The measurements were conducted in a mass range of m/z 70 to 1,050 in positive and negative mode. Data was evaluated by using FreeStyle 1.6 (Thermo Fisher Scientific). Peaks were detected with the CAD detector and the relative absorbance of deletion, overexpression and complementation strains were compared to wildtype. The experiment was at least repeated twice. Reproducible main peaks were analysed in detail by comparing their sum formula (calculated from exact mass) and MS2 spectra to literature. Peaks, which could not be assigned to a mass were not further investigated. Chromatograms in the results section display an example of metabolites extracted from the mentioned strains. Examples of single ion monitoring of selected compounds and strains are shown in the supplementary data.

For the quantification of scytalone the corresponding peaks of the extracted ion chromatograms (EICs) at m/z = 193.0506 ± 5 ppm were detected and peak areas were determined with the PPD algorithm with a signal to noise ratio of 5 in FreeStyle 1.6.75.20 (Thermo Fisher Scientific). Each experiment was performed with one replicate (n = 1). Bars represent the mean values of all replicates and error bars correspond to standard deviations. Statistical significances were calculated by t-test with the SISA online tool [84]. Significances indicate: **:p<0.01; ***:p<0.001.

### Protein extraction

Proteins were extracted as described [88] using ground fungal mycelium, harvested from cultures, grown under the indicated conditions. Protein concentrations were determined in a modified Bradford assay using Roti Quant solution (Carl Roth).

### *In vitro* protein pull-down

5x10^7^ freshly harvested spores were inoculated in 500 ml PDM and grown for 72 h during constant agitation in light. Proteins of VGB39, VGB45, VGB447, VGB450, VGB451 and VGB453 were extracted. The buffer was supplemented with 2 mM dithiothreitol, 1 mM phenylmethylsulfonylfluorid and 10 μl/ml cOmplete Protease Inhibitor Cocktail (Roche, stock: one tablet dissolved in 500 μl dH_2_O). VGB45 and VGB39 were mixed before protein extraction (1/3 VGB45, 2/3 VGB39) due to the higher amount of free GFP in VGB45 in comparison to the above mentioned velvet strains, which was observed with Western experiments using a GFP-specific antibody. Prior to protein loading, the beads were washed with buffer in the column. For each sample, 12 mg of protein were applied to a GFP-trap pull-down with 10 μl GFP-TrapA beads (Chromotek) in a polyprep column (Bio-Rad). The final volume was adjusted to the same amount with extraction buffer. Beads were incubated for 2 h at 4°C rotating. Afterwards, the beads were washed twice with W300 buffer (300 mM NaCl, 10 mM Tris pH 7.5). To elute proteins, 150 μl glycine pH 2.5 were pipetted into the column and mixed with 15 μl 1 M Tris pH 10.4. The elution step was repeated twice leading to elution fractions E1-E3. Eluted protein was applied to chloroform-methanol extraction [89] and digested with trypsin. Peptides were purified with StageTips [90,91]. Further details can be found in the supporting information (S1 Text and S12 Table). The Service Unit conducted LC/MS analysis. Mass spectrometry proteomics data have been deposited to the ProteomeXchange Consortium via the PRIDE [92] partner repository with the dataset identifier PXD02861.

### Western hybridization

Fungal strains were grown in liquid PDM, liquid SXM or on SXM plates covered with a nylon membrane (GE Healthcare). Mycelium was harvested and ground in liquid nitrogen at the indicated time points. After extraction of proteins, western analysis was conducted as previously described [56,88]. For the visualization of GFP fusion proteins, the α-GFP antibody (sc-9996, Santa Cruz Biotechnology) was applied. A horseradish peroxidase-coupled secondary antibody was used (mouse 115-035-003 antibody, Jackson ImmunoResearch).

## Supporting information

S1 Fig Comparison of Vel1-like proteins of *V. dahliae* and different fungi. Deduced proteins of *Aspergillus fumigatus* Af293, *Aspergillus nidulans* FGSC A4, *Botrytis cinerea* BcDW1, *Colletotrichum graminicola* M1.001, *Magnaporthe oryzae* M68 and *Neurospora crassa* OR74A similar to Vel1 of *V*. *dahliae* JR2 were aligned by MegAlignPro (DNASTAR) using ClustalW multiple sequence alignment. The velvet domain predicted by InterProScan for the Vel1 protein (VDAG_JR2_Chr7g04890a) is depicted in dark blue according to IPR037525. The predicted NLS is shown in grey and the PEST motif in purple.
(TIF)

S2 Fig Comparison of Vel2-like proteins of *V. dahliae* and different fungi. Deduced proteins of *A*. *fumigatus* Af293, *A*. *nidulans* FGSC A4, *B*. *cinerea* BcDW1, *C*. *graminicola* M1.001, *M*. *oryzae* M68 and *N*. *crassa* OR74A similar to Vel2 of *V*. *dahliae* JR2 were aligned by MegAlignPro (DNASTAR) using ClustalW multiple sequence alignment. The velvet domain predicted by InterProScan according to IPR037525 for the Vel2 protein (VDAG_JR2_Chr3g06150a) is depicted in dark blue. Homologies to other ascomycetes indicate that all Vel2-like velvet domains are interrupted by an intrinsically disordered domain (IDD), which is indicated by an orange color. The predicted NLS is shown in grey.
(TIF)

S3 Fig Comparison of Vel3-like proteins of *V. dahliae* and different fungi. Deduced proteins of *A*. *fumigatus* Af293, *A*. *nidulans* FGSC A4, *B*. *cinerea* BcDW1, *C*. *graminicola* M1.001, *M*. *oryzae* M68 and *N*. *crassa* OR74A similar to Vel3 of *V*. *dahliae* JR2 were aligned by MegAlignPro (DNASTAR) using ClustalW multiple sequence alignment. The velvet domain predicted by InterProScan for the Vel3 protein (VDAG_JR2_Chr6g00630a) is depicted in dark blue. The domain is shown according to IPR037525. In grey, the predicted NLS is shown. The PEST motif is indicated in purple.
(TIF)

S4 Fig Comparison of Vos1-like proteins of *V. dahliae* and different fungi. Deduced proteins of *A*. *fumigatus* Af293, *A*. *nidulans* FGSC A4, *B*. *cinerea* BcDW1, *C*. *graminicola* M1.001, *M*. *oryzae* M68 and *N*. *crassa* OR74A similar to Vos1 of *V*. *dahliae* JR2 were aligned by MegAlignPro (DNASTAR) using ClustalW multiple sequence alignment. The sequence used for the alignment is an improved version of the VDAG_JR2_Chr3g12090a prediction, which derived from cDNA sequencing and mass spectrometry verification of the protein. The Vos1 velvet domain predicted by InterProScan is depicted in dark blue according to IPR037525.
(TIF)

S5 Fig Velvet proteins are conserved in different fungi. Deduced proteins of *A*. *fumigatus* Af293, *A*. *nidulans* FGSC A4, *B*. *cinera* BcDW1, *C*. *graminicola* M1.001, *M*. *oryzae* M68 and *N*. *crassa* OR74A similar to *V*. *dahliae* Vel1, Vel2, Vel3 or Vos1, respectively, were aligned by MegAlignPro (DNASTAR) using ClustalW multiple sequence alignment. Similarities of *V*. *dahliae* velvet proteins in different fungi are shown in sequence identity matrices and phylogenetic trees **(A)** Vel1, **(B)** Vel2, **(C)** Vel3 and **(D)** Vos1. **(E)** Amino acid sequence predicted by Ensembl Fungi is within the box, amino acids deduced from cDNA sequencing and missing in the prediction are outside of the box. The sequence highlighted in green was covered with peptides identified with LC/MS and MaxQuant 1.6.0.16 analysis. Black parts of the sequence were not covered.
(TIF)

S6 Fig Southern hybridization of the *V. dahliae VEL1* and *VEL2* deletion, double deletion and complementation strains. Genomic DNA (gDNA) was extracted from the wild type (WT), two independent transformants of the *VEL1* deletion strain (Δ*VEL1*) with hygromycin resistance cassette (*HYG*), two independent transformants of the *VEL2* deletion strain (Δ*VEL2*) with nourseothricin resistance cassette (*NAT*), the complementation strains of *VEL1* and *VEL2* (Comp. *VEL1*, Comp. *VEL2*) with nourseothricin resistance cassette (*NAT*) or hygromycin resistance cassette (*HYG*), respectively and two independent transformants of *VEL1* and *VEL2* (Δ*VEL1*/Δ*VEL2*) or *VEL3* (Δ*VEL3*/Δ*VEL1*) double deletion strains. gDNA was restricted with *Xho*I and used for Southern hybridization with the 3’ region labeled as a probe for verification of the *VEL1* deletion or complementation strains. gDNA was restricted with *Bgl*II for verification of the *VEL2* deletion and complementation strains and the 3’ region was used as probe. **(A)** Restriction sites of *Xho*I in the wild type, deletion and complementation strains and binding site of the 3’ probe (marked in blue). For each strain the expected fragment size is depicted. **(B)** Southern hybridization of the *VEL1* deletion and complementation strains with wild type control. For the wild type, a fragment of 3.5 kb was obtained. Both tested *VEL1* deletion strains resulted in a fragment with a size of 5 kb. The complementation strain shows a fragment with a size of 5.7 kb. **(C)** Southern hybridization of the *VEL1* single, *VEL1*/*VEL2* as well as *VEL3*/*VEL1* double deletion strains and wild type control. The wild type exhibits a fragment with a size of 3.5 kb. All strains containing the deletion cassette of *VEL1* result in a fragment with a size of 5 kb. **(D)** Restriction sites of *Bgl*II in the wild type, deletion and complementation strains. The binding site of the 3’region, which was used as probe, is marked in blue. The expected fragment size for each strain is shown. **(E)** Southern hybridization of the *VEL2* deletion and the complementation strains with wild type control. The wild type exhibits a fragment with the size of 3.3 kb. The tested deletion strains show fragments with a size of 3 kb. The complementation strain results in a fragment with a size of approximately 6.6 kb. **(F)** Southern hybridization of the wild type, *VEL2* deletion and *VEL1* and *VEL2* double deletion strains restricted with *Bgl*II and incubated with the 3’ probe. The wild type exhibits a fragment size of 3.3 kb. Strains containing the *VEL2* deletion cassette show a fragment with a size of 3 kb.
(TIF)

S7 Fig Southern hybridization of the *V. dahliae VEL3* and *VOS1* deletion, double deletion and complementation strains. Genomic DNA (gDNA) of the wild type (WT), two independent transformants of the *VEL3* deletion strain (Δ*VEL3*) with nourseothricin resistance cassette (*NAT*), two independent transformants of the *VOS1* deletion strain (Δ*VOS1*) with hygromycin or nourseothricin resistance cassette (*HYG, NAT*), the *VEL3* complementation strain (Comp. *VEL3*) with hygromycin resistance cassette (*HYG*) and two independent transformants of the *VEL3* and *VEL1* (Δ*VEL3*/Δ*VEL1*) as well as a single transformant of *VOS1* and *VEL3* (Δ*VOS1*/Δ*VEL3*) double deletion strains was extracted and used for Southern hybridization. For verification of the *VEL3* deletion and complementation strains, the restriction enzyme *Xho*I was used and the 5’ region as a probe. For restriction of gDNA from the *VOS1* deletion strains, *Bgl*I was used and the 3’region severed as probe. **(A)** Restriction sites of *Xho*I in the wild type, deletion and complementation strains. The binding site of the 5’ region which was used as probe is marked in blue. **(B)** Southern hybridization of the *VEL3* deletion and the complementation strains with wild type control. The wild type results in a fragment with a size of approximately 2.7 kb. The two tested deletion transformants exhibit fragments with a size of 6.4 kb. The complementation shows a fragment with a size of 2.7 kb. **(C)** Southern hybridization of the *VEL3* deletion and *VEL3* and *VEL1* as well as *VOS1* and *VEL3* double deletion strains with wild type control. The wild type results in a fragment of 2.7 kb. Strains containing the *VEL3* deletion cassette exhibit a fragment with a size of 6.4 kb. **(D)** Restriction sites of *Bgl*I in the wild type and deletion strains. The 3’ flanking region was used as probe (marked by a blue line). **(E)** Southern hybridization of *VOS1* deletion strains with hygromycin resistance cassette (*HYG*) with wild type control. The wild type exhibits a fragment with a size of 4.8 kb. The two tested *VOS1* deletion transformants show fragments with a size of 1.8 kb. **(F)** Southern hybridization of the *VOS1* deletion with hygromycin resistance cassette (*HYG*), double deletion strains with *VEL3* and wild type control. The wild type results in a fragment with a size of 4.8 kb. *VOS1* deletion strains show fragments with a size of 1.8 kb. **(G)** Southern hybridization of the *VOS1* deletion strains with nourseothricin resistance cassette with wild type control. The labeled fragment of the wild type has a size of 4.8 kb. The two tested deletion transformants exhibit a fragment with a size of 1.8 kb.
(TIF)

S8 Fig *V. dahliae VOS1* function is independently of microsclerotia development. Images give an overview of fungal colonies after spotting 5x10^4^ spores of indicated *V*. *dahliae* strains on either simulated xylem medium (SXM) or Czapek-Dox-Medium (CDM) and subsequent incubation for 10 days at 25°C in constant light or darkness. Single colonies on SXM are shown from the back and colonies on CDM are shown from the top of the plate. Cross sections were made through the middle of the colony. **(A)** Growth and development of the *VOS1* deletion (Δ*VOS1*) and the wild type (WT) strains are similar. Single colonies (left column) and colony cross sections (right column) are shown. **(B)** A *VEL3* and *VOS1* double deletion strain (Δ*VEL3*/Δ*VOS1*) resembles the phenotype of the *VEL3* (Δ*VEL3*) single deletion strain.
(TIF)

S9 Fig Chromatograms of *V. dahliae* metabolites in darkness or light and gene expression of *CMR1*. **(A)** LC/MS combined with photodiode array detection (PDA) analysis of secondary metabolites extracted from the *V*. *dahliae* wild type and deletion strains of *VEL1* and *VEL2* in darkness and light. Secondary metabolites were extracted from two-week-old fungal mycelium grown on Czapek-Dox-Medium (CDM) supplemented with glucose. Chromatograms of the wild type and both deletion strains exhibit the same presence and absence of secondary metabolites in darkness or light. **(B)** Quantification of *CMR1* gene expression in the wild type and the *VEL1* deletion strain. Mean values of three independent experiments with standard deviations relative to the wild type are depicted. Normalization was conducted to the reference genes *H2A* and *EIF2B*. Significant differences were calculated by t-test and indicate: *:p<0.05.
(TIF)

S10 Fig Single ion monitoring and MS2 spectrum for substance I from *V. dahliae* metabolite extracts. **(A)** and **(B)** Single ion monitoring (SIM) for substance I with m/z 193.0493 and a mass tolerance of 5.00 ppm in negative ion mode. Depicted are the wild type (WT), *VEL1* deletion strain (Δ*VEL1*) and complementation (Comp. *VEL1*) (A) as well as the wild type (WT), *VEL2* deletion strain (Δ*VEL2*) and complementation (Comp. *VEL2*) (B). **(C)** MS2 spectrum of substance I.
(TIF)

S11 Fig Single ion monitoring and MS2 spectrum for substance II from *V. dahliae* metabolite extracts. **(A)** and **(B)** Single ion monitoring (SIM) for substance II with m/z 235.0603 and a mass tolerance of 5.00 ppm in negative ion mode. Depicted are the wild type (WT), *VEL1* deletion strain (Δ*VEL1*) and complementation (Comp. *VEL1*) (A) as well as the wild type (WT), *VEL2* deletion strain (Δ*VEL2*) and complementation (Comp. *VEL2*) (B). **(C)** MS2 spectrum of substance II.
(TIF)

S12 Fig Single ion monitoring and MS2 spectrum for substance III from *V. dahliae* metabolite extracts. **(A)** and **(B)** Single ion monitoring (SIM) for substance III with m/z 193.0499 and a mass tolerance of 5.00 ppm in positive ion mode. Depicted are the wild type (WT), *VEL1* deletion strain (Δ*VEL1*) and complementation (Comp. *VEL1*) (A) as well as the wild type (WT), *VEL2* deletion strain (Δ*VEL2*) and complementation (Comp. *VEL2*) (B). **(C)** MS2 spectrum of substance III.
(TIF)

S13 Fig Single ion monitoring and MS2 spectrum for substance IV from *V. dahliae* metabolite extracts. **(A)** and **(B)** Single ion monitoring (SIM) for substance IV with m/z 577.3732 and a mass tolerance of 5.00 ppm in positive ion mode. Depicted are the wild type (WT), *VEL1* deletion strain (Δ*VEL1*) and complementation (Comp. *VEL1*) (A) as well as the wild type (WT), *VEL2* deletion strain (Δ*VEL2*) and complementation (Comp. *VEL2*) (B). **(C)** MS2 spectrum of substance IV.
(TIF)

S14 Fig Single ion monitoring and MS2 spectrum for substance V from *V. dahliae* metabolite extracts. **(A)** and **(B)** Single ion monitoring (SIM) for substance V with m/z 591.3887 and a mass tolerance of 5.00 ppm in positive ion mode. Depicted are the wild type (WT), *VEL1* deletion strain (Δ*VEL1*) and complementation (Comp. *VEL1*) (A) as well as the wild type (WT), *VEL2* deletion strain (Δ*VEL2*) and complementation (Comp. *VEL2*) (B). **(C)** MS2 spectrum of substance V.
(TIF)

S15 Fig Single ion monitoring and MS2 spectrum for *V. dahliae* substances X and XI. **(A)** Single ion monitoring (SIM) for substance X with m/z 331.0606 and a mass tolerance of 5.00 ppm in negative ion mode. Depicted are the wild type (WT) and the overexpression of *VEL1* (*OE-VEL1*). **(B)** MS2 spectrum of substance X. **(C)** Single ion monitoring (SIM) for substance XI with m/z 317.0811 and a mass tolerance of 5.00 ppm in negative ion mode. Depicted are the wild type (WT) and the overexpression of *VEL1* (*OE-VEL1*). **(D)** MS2 spectrum of substance XI.
(TIF)

S16 Fig Chromatograms of *V. dahliae* metabolites with selected single ion monitoring and scytalone quantification. **(A)** LC/MS combined with photodiode array detection (PDA) analysis of secondary metabolites. Secondary metabolites were extracted from two-week-old fungal mycelium grown on Czapek-Dox-Medium (CDM) supplemented with glucose. Depicted are the wild type (WT), *VEL3* deletion strain (Δ*VEL3*) and a *VEL3* complementation strain (Comp. *VEL3*). All three chromatograms look similar, but increased relative absorbance was observed for the substances I-V in the deletion strain. **(B)** Selected single ion monitoring (SIM) for the seven substances with increased relative abundance in the *VEL3* deletion strain in comparison to wild type and complementation (black: wild type, red: Δ*VEL3*, green: Comp. *VEL3*). Substance I: m/z 193.0493, mass tolerance 5.00 ppm; substance II: m/z 235.0603, mass tolerance 5.00 ppm; substance III: m/z 193.0499, mass tolerance 5.00 ppm; substance IV: m/z 577.3722, mass tolerance 5.00 ppm; substance V: m/z 591.3887, mass tolerance 5.00 ppm; substance X: m/z 331.0606, mass tolerance 5.00 ppm; substance XI: m/z 317.0811, mass tolerance 5.00 ppm. **(C)** Quantification of scytalone production relative to the wild type in the *VEL3* deletion (Δ*VEL3*) strain and the overexpression strain of VEL1 (*OE*-*VEL1*). In the left part an example of the scytalone peak is depicted for the wild type, the *VEL3* deletion and the *VEL1* overexpression strain. In the right part the size of the scytalone peak of at least two independent replicates was quantified relative to the wild type. Significant differences were calculated by t-test and indicate: **:p<0.01; ***:p<0.001.
(TIF)

S17 Fig Phenotypical characterization of *V. dahliae* strains used in this study. From the indicated strains 5x10^4^ spores were spotted on plates (SXM: simulated xylem medium; CDM: minimal Czapek-Dox-Medium) and incubated for 10 days at 25°C. Single colonies on SXM are shown from the back, colonies on CDM are shown from the top of the plate. Cross sections were made through the middle of the colony. **(A)** Lae1 is dispensable for microsclerotia formation. The *LAE1* deletion strain has a similar phenotype as the wild type when grown on SXM or CDM. **(B)** Velvet proteins fused C-terminally to GFP under the control of their native promotor show no phenotypic alterations compared to the wild type. **(C)** Overexpression strains of velvet proteins fused to GFP and transformed with a strain with histone H2B-RFP labeled nuclei have a wild type-like appearance. **(D)** Overexpression strains of *VEL1* and *VEL2* fused to *GFP* and transformed into the deletion strain of *VEL2* or *VEL1*, respectively, have a phenotype similar to the deletion strain. **(E)** Phenotype of a wild type ectopically expressing high amounts of free GFP and *VEL1* deletion strain expressing the same construct.
(TIF)

S18 Fig Chromatograms of metabolites extracted from *VOS1* and *LAE1* deletion strains. LC/MS combined with photodiode array detection (PDA) analysis of secondary metabolites. Secondary metabolites were extracted from two-week-old fungal mycelium grown on Czapek-Dox-Medium (CDM) supplemented with glucose. Chromatograms of *VOS1* and *LAE1* deletion strains resemble the wild type.
(TIF)

S19 Fig Single ion monitoring and MS2 spectrum for *V. dahliae* substance VI and VII. **(A)** Single ion monitoring (SIM) for substance VI with m/z 263.1284 and a mass tolerance of 5.00 ppm in negative ion mode. Depicted are the wild type (WT), *VEL1* deletion strain (Δ*VEL1*) and complementation (Comp. *VEL1*). **(B)** MS2 spectrum of substance VI. **(C)** Single ion monitoring (SIM) for substance VII with m/z 351.1264 and a mass tolerance of 5.00 ppm in negative ion mode. Depicted are the wild type (WT), *VEL1* deletion strain (Δ*VEL1*) and complementation (Comp. *VEL1*). **(D)** MS2 spectrum of substance VII.
(TIF)

S20 Fig Single ion monitoring and MS2 spectrum for *V. dahliae* substance VIII an IX. **(A)** Single ion monitoring (SIM) for substance VIII with m/z 233.1544 and a mass tolerance of 5.00 ppm in positive ion mode. Depicted are the wild type (WT), *VEL1* deletion strain (Δ*VEL1*) and complementation (Comp. *VEL1*). **(B)** MS2 spectrum of substance VIII. **(C)** Single ion monitoring (SIM) for substance IX with m/z 249.1483 and a mass tolerance of 5.00 ppm in positive ion mode. Depicted are the wild type (WT), *VEL1* deletion strain (Δ*VEL1*) and complementation (Comp. *VEL1*). **(D)** MS2 spectrum of substance IX.
(TIF)

S21 Fig Light-independent nuclear localization of V. *dahliae* velvet domain proteins. Subcellular localization analysis of fusions of Vel1, Vel2, Vel3 and Vos1 with GFP by fluorescence microscopy. 1x10^4^ freshly harvested spores were incubated in PDM for 16h at 25°C in light or darkness. Differential interference contrast (DIC), green fluorescent filter view (GFP), red fluorescent filter view (RFP) and a merge of GFP and RFP channels are shown. Scale bar = 10 μm. **(A)** Velvet protein subpopulations are primarily localized to the nucleus in light as well as in darkness with additional small Vel2 subpopulations in the cytoplasm. Nuclei are visualized by histone H2B-RFP. **(B)** High levels of Vel1 or Vel2 can enter the nucleus independently of each other in light or darkness in Δ*VEL1* and Δ*VEL2* strains, respectively. **(C)** Controls for subcellular localization studies in *V*. *dahliae*. The wild type (WT) was used as negative control, a strain with overexpressed *GFP* (WT/*OE-GFP*) as positive control and a strain in which histone *H2B-RFP* is overexpressed (WT/*HISTONE-RFP*) as positive control.
(TIF)

S22 Fig V. *dahliae* Vel2 abundance depends on illumination. 1x10^6^ freshly harvested spores were inoculated for analysis of protein abundances in liquid potato dextrose medium (PDM) for filamentous growth, in liquid pectin-rich simulated xylem medium (SXM) for conidia formation and on SXM plates covered with a nylon membrane for microsclerotia development. The fungus was grown for three and six days at 25°C in light or darkness. Western hybridization with a GFP antibody was performed with crude extracts of the indicated strains (free GFP: 27 kDa; Vel2-GFP: 78 kDa). Presence (+) or absence (-) of full-length protein during growth in light or darkness during different developmental conditions (hyphal growth in PDM, spore production in liquid SXM, microsclerotia formation on SXM plates) after three and six days. Vel2 is stable during filamentous growth, destabilized during conidiation in light and initially more stable in light than in darkness during microsclerotia formation.
(TIF)

S1 Text Supporting Information Methods. (PDF)

S1 Data Supplementary data file. (XLSX)

S1 Table Proteins significantly enriched with Vel1-GFP and their predicted domains and functions. (PDF)

S2 Table Proteins significantly enriched with Vel2-GFP and their predicted domains and functions. (PDF)

S3 Table Proteins significantly enriched with Vel3-GFP and their predicted domains and functions. (PDF)

S4 Table Proteins significantly enriched with Vos1-GFP and their predicted domains and functions. (PDF)

S5 Table Significantly enriched proteins with LFQ intensities, MS/MS count, sequence coverage and unique peptides in all three replicates of Vel1-GFP in comparison to the wild type. (PDF)

S6 Table Significantly enriched proteins with LFQ intensities, MS/MS count, sequence coverage and unique peptides in all three replicates of Vel2-GFP in comparison to the wild type. (PDF)

S7 Table Significantly enriched proteins with LFQ intensities, MS/MS count, sequence coverage and unique peptides in all three replicates of Vel3-GFP in comparison to the wild type. (PDF)

S8 Table Significantly enriched proteins with LFQ intensities, MS/MS count, sequence coverage and unique peptides in all three replicates of Vos1-GFP in comparison to the wild type. (PDF)

S9 Table *A. tumefaciens*, *E. coli* and *V. dahliae* strains used in this study. (PDF)

S10 Table Primers used in this study. (PDF)

S11 Table Plasmids used in this study. (PDF)

S12 Table Perseus workflow for evaluation of the MaxQuant result files for the *in vitro* protein pull-downs. (PDF)
