# Supplementary figures and images for: The velvet protein Vel1 controls initial plant root colonization and conidia formation for xylem distribution in Verticillium wilt
Source: PLoS Genet. 2021 Mar 15;17(3):e1009434. doi: 10.1371/journal.pgen.1009434 (PMC7993770; doi:10.1371/journal.pgen.1009434)

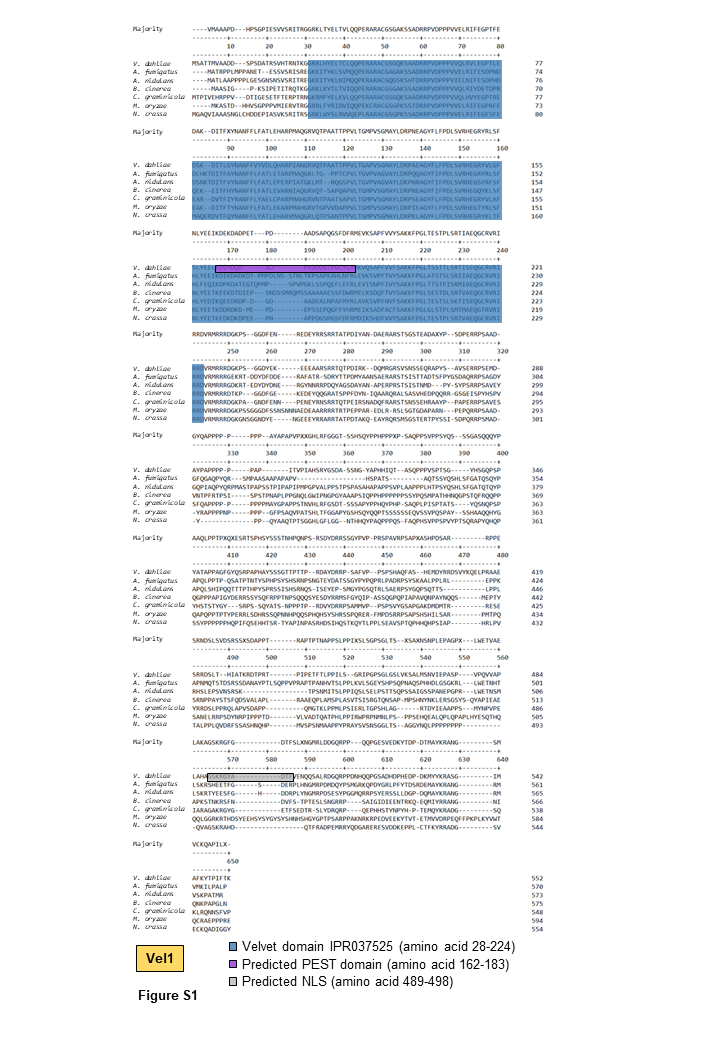

Supplement: S1 Fig — Deduced proteins of Aspergillus fumigatus Af293, Aspergillus nidulans FGSC A4, Botrytis cinerea BcDW1, Colletotrichum graminicola M1.001, Magnaporthe oryzae M68 and Neurospora crassa OR74A similar to Vel1 of V. dahliae JR2 were aligned by MegAlignPro (DNASTAR) using ClustalW multiple sequence alignment. The velvet domain predicted by InterProScan for the Vel1 protein (VDAG_JR2_Chr7g04890a) is depicted in dark blue according to IPR037525. The predicted NLS is shown in grey and the PEST motif in purple. (TIF) [file pgen.1009434.s001.TIF]

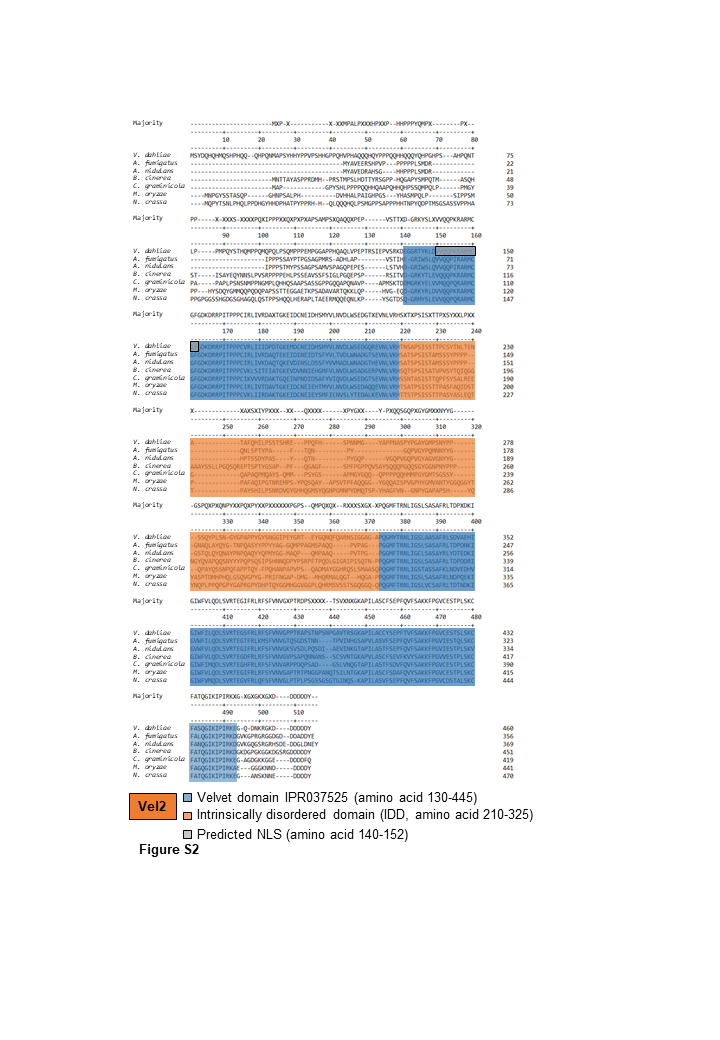

Supplement: S2 Fig — Deduced proteins of A. fumigatus Af293, A. nidulans FGSC A4, B. cinerea BcDW1, C. graminicola M1.001, M. oryzae M68 and N. crassa OR74A similar to Vel2 of V. dahliae JR2 were aligned by MegAlignPro (DNASTAR) using ClustalW multiple sequence alignment. The velvet domain predicted by InterProScan according to IPR037525 for the Vel2 protein (VDAG_JR2_Chr3g06150a) is depicted in dark blue. Homologies to other ascomycetes indicate that all Vel2-like velvet domains are interrupted by an intrinsically disordered domain (IDD), which is indicated by an orange color. The predicted NLS is shown in grey. (TIF) [file pgen.1009434.s002.TIF]

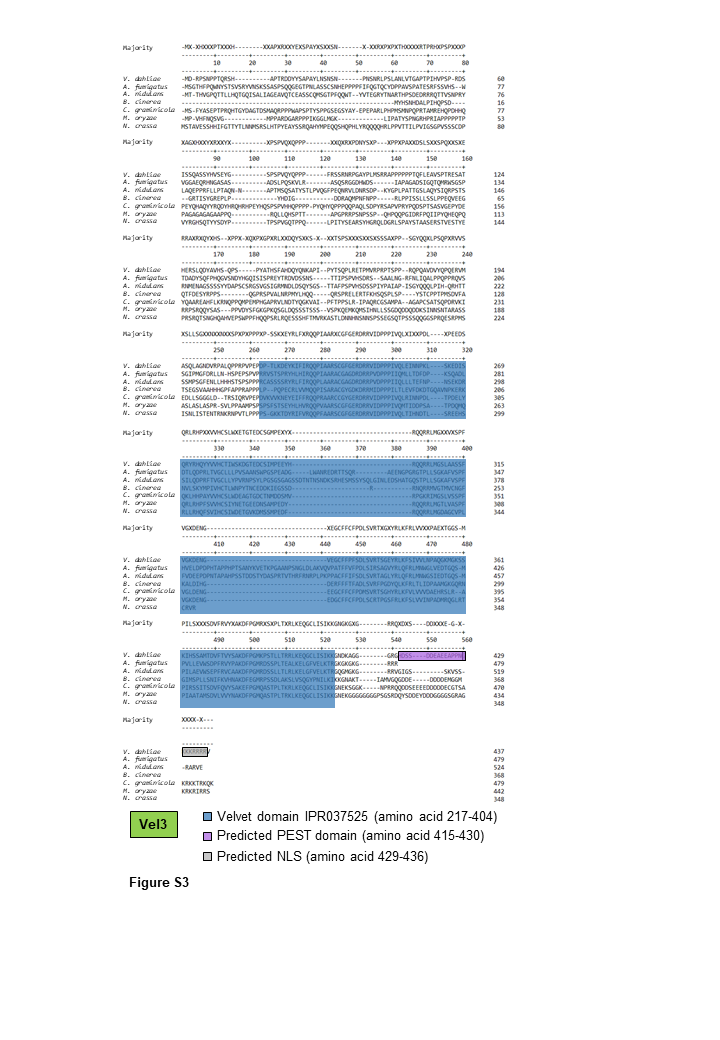

Supplement: S3 Fig — Deduced proteins of A. fumigatus Af293, A. nidulans FGSC A4, B. cinerea BcDW1, C. graminicola M1.001, M. oryzae M68 and N. crassa OR74A similar to Vel3 of V. dahliae JR2 were aligned by MegAlignPro (DNASTAR) using ClustalW multiple sequence alignment. The velvet domain predicted by InterProScan for the Vel3 protein (VDAG_JR2_Chr6g00630a) is depicted in dark blue. The domain is shown according to IPR037525. In grey, the predicted NLS is shown. The PEST motif is indicated in purple. (TIF) [file pgen.1009434.s003.TIF]

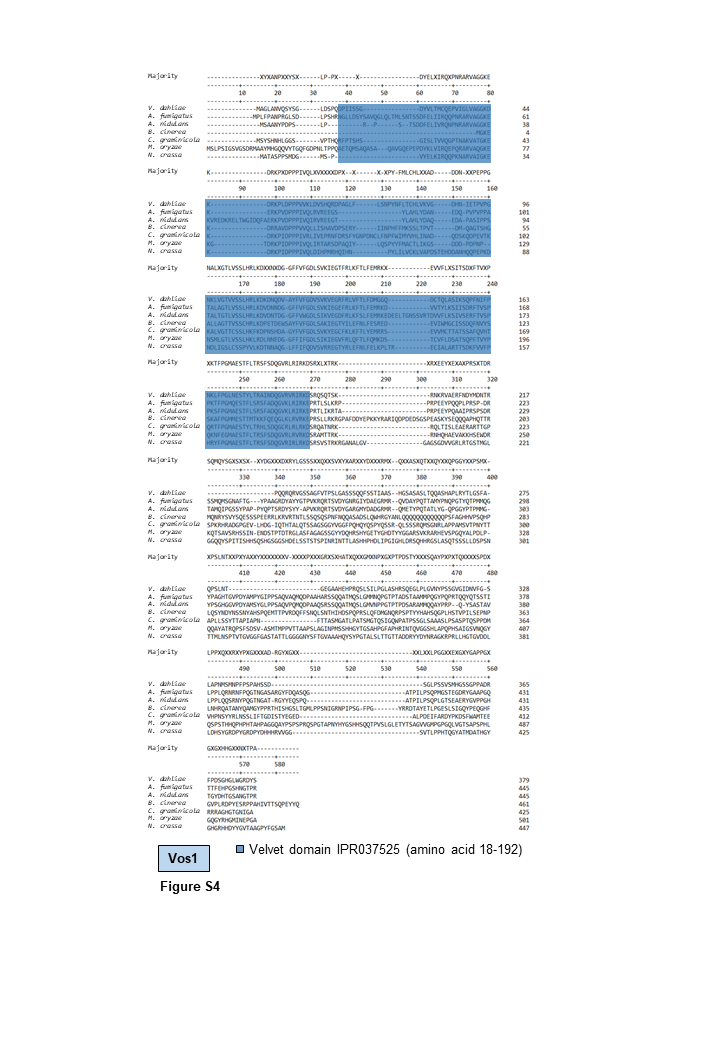

Supplement: S4 Fig — Deduced proteins of A. fumigatus Af293, A. nidulans FGSC A4, B. cinerea BcDW1, C. graminicola M1.001, M. oryzae M68 and N. crassa OR74A similar to Vos1 of V. dahliae JR2 were aligned by MegAlignPro (DNASTAR) using ClustalW multiple sequence alignment. The sequence used for the alignment is an improved version of the VDAG_JR2_Chr3g12090a prediction, which derived from cDNA sequencing and mass spectrometry verification of the protein. The Vos1 velvet domain predicted by InterProScan is depicted in dark blue according to IPR037525. (TIF) [file pgen.1009434.s004.TIF]

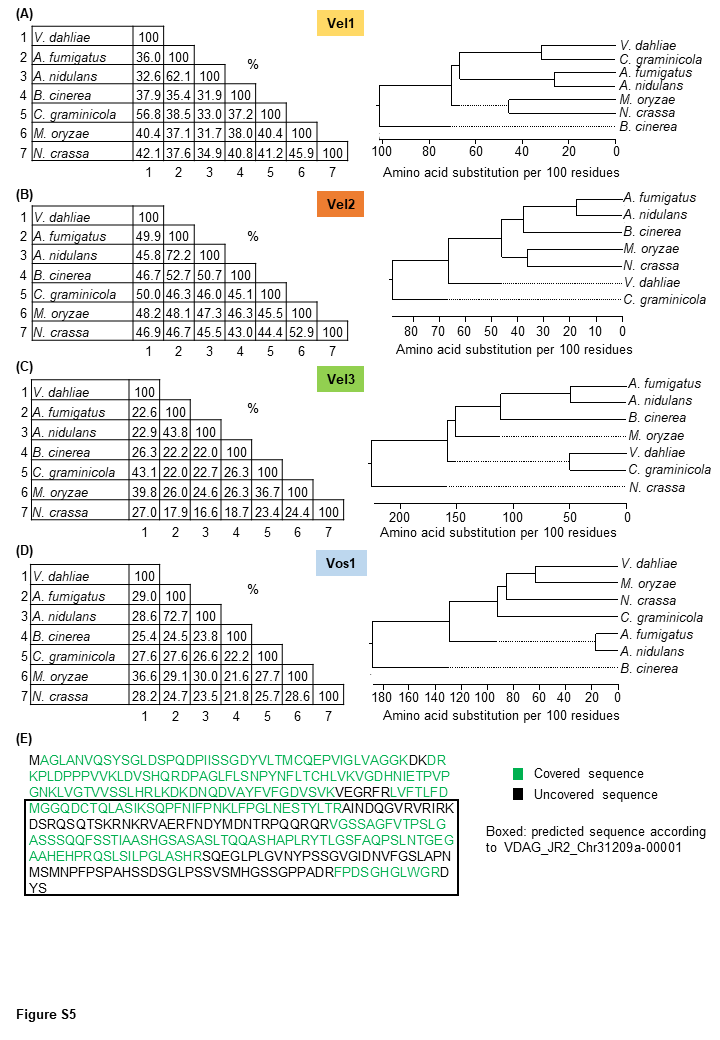

Supplement: S5 Fig — Deduced proteins of A. fumigatus Af293, A. nidulans FGSC A4, B. cinera BcDW1, C. graminicola M1.001, M. oryzae M68 and N. crassa OR74A similar to V. dahliae Vel1, Vel2, Vel3 or Vos1, respectively, were aligned by MegAlignPro (DNASTAR) using ClustalW multiple sequence alignment. Similarities of V. dahliae velvet proteins in different fungi are shown in sequence identity matrices and phylogenetic trees (A) Vel1, (B) Vel2, (C) Vel3 and (D) Vos1. (E) Amino acid sequence predicted by Ensembl Fungi is within the box, amino acids deduced from cDNA sequencing and missing in the prediction are outside of the box. The sequence highlighted in green was covered with peptides identified with LC/MS and MaxQuant 1.6.0.16 analysis. Black parts of the sequence were not covered. (TIF) [file pgen.1009434.s005.TIF]

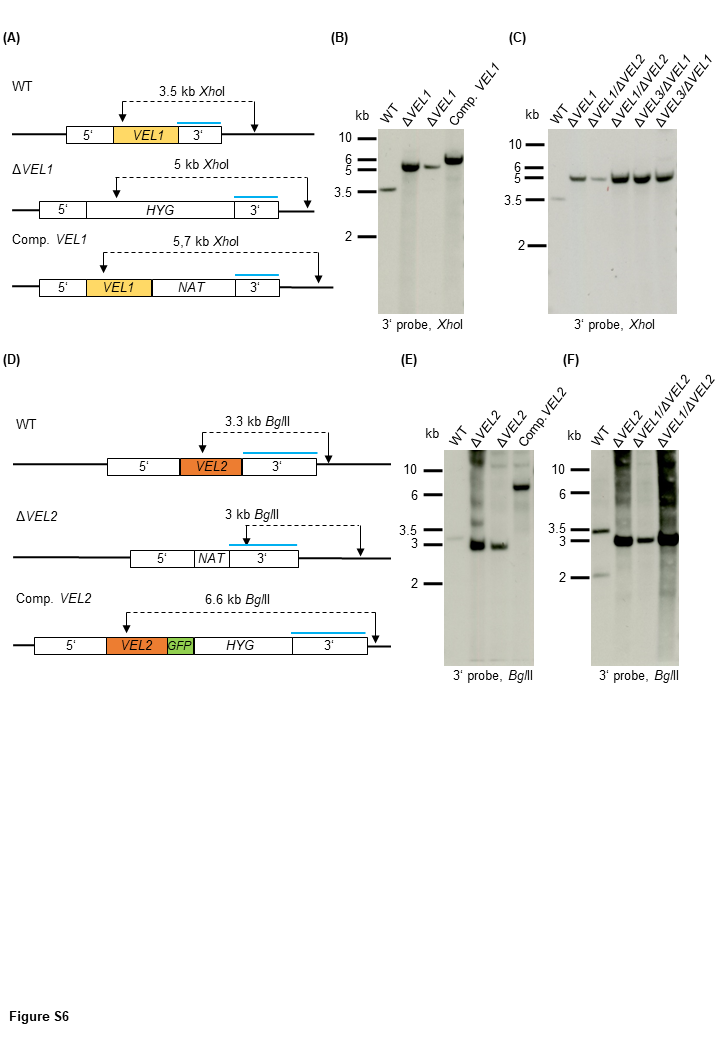

Supplement: S6 Fig — Genomic DNA (gDNA) was extracted from the wild type (WT), two independent transformants of the VEL1 deletion strain (ΔVEL1) with hygromycin resistance cassette (HYG), two independent transformants of the VEL2 deletion strain (ΔVEL2) with nourseothricin resistance cassette (NAT), the complementation strains of VEL1 and VEL2 (Comp. VEL1, Comp. VEL2) with nourseothricin resistance cassette (NAT) or hygromycin resistance cassette (HYG), respectively and two independent transformants of VEL1 and VEL2 (ΔVEL1/ΔVEL2) or VEL3 (ΔVEL3/ΔVEL1) double deletion strains. gDNA was restricted with XhoI and used for Southern hybridization with the 3’ region labeled as a probe for verification of the VEL1 deletion or complementation strains. gDNA was restricted with BglII for verification of the VEL2 deletion and complementation strains and the 3’ region was used as probe. (A) Restriction sites of XhoI in the wild type, deletion and complementation strains and binding site of the 3’ probe (marked in blue). For each strain the expected fragment size is depicted. (B) Southern hybridization of the VEL1 deletion and complementation strains with wild type control. For the wild type, a fragment of 3.5 kb was obtained. Both tested VEL1 deletion strains resulted in a fragment with a size of 5 kb. The complementation strain shows a fragment with a size of 5.7 kb. (C) Southern hybridization of the VEL1 single, VEL1/VEL2 as well as VEL3/VEL1 double deletion strains and wild type control. The wild type exhibits a fragment with a size of 3.5 kb. All strains containing the deletion cassette of VEL1 result in a fragment with a size of 5 kb. (D) Restriction sites of BglII in the wild type, deletion and complementation strains. The binding site of the 3’region, which was used as probe, is marked in blue. The expected fragment size for each strain is shown. (E) Southern hybridization of the VEL2 deletion and the complementation strains with wild type control. The wild type exhibits a fragment with the s [file pgen.1009434.s006.TIF]

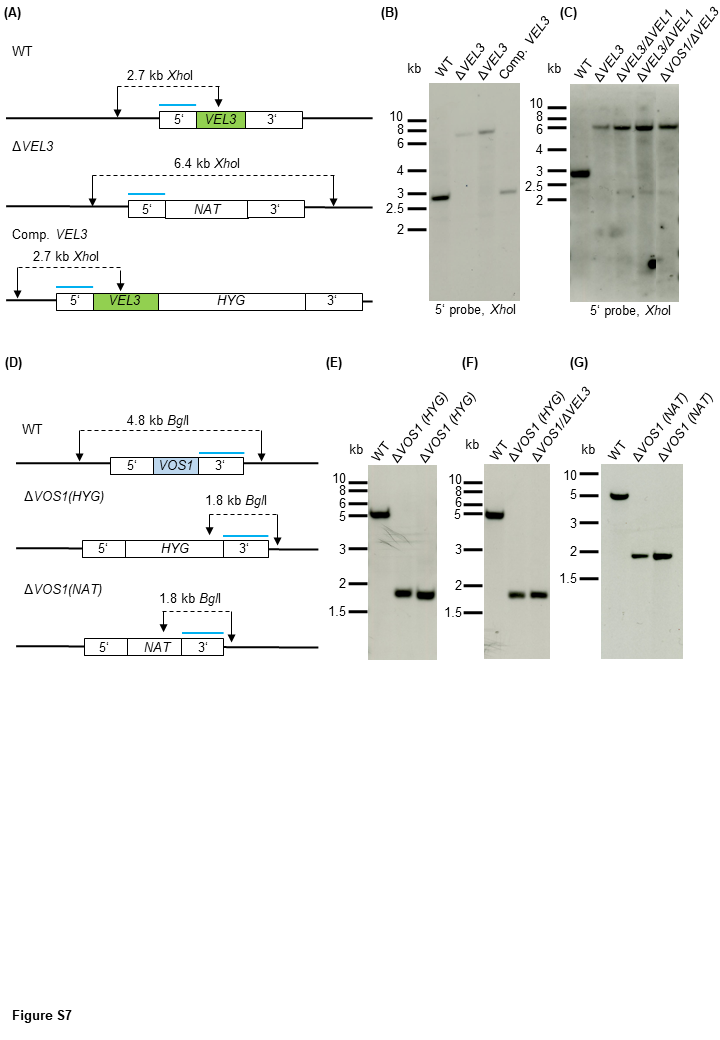

Supplement: S7 Fig — Genomic DNA (gDNA) of the wild type (WT), two independent transformants of the VEL3 deletion strain (ΔVEL3) with nourseothricin resistance cassette (NAT), two independent transformants of the VOS1 deletion strain (ΔVOS1) with hygromycin or nourseothricin resistance cassette (HYG, NAT), the VEL3 complementation strain (Comp. VEL3) with hygromycin resistance cassette (HYG) and two independent transformants of the VEL3 and VEL1 (ΔVEL3/ΔVEL1) as well as a single transformant of VOS1 and VEL3 (ΔVOS1/ΔVEL3) double deletion strains was extracted and used for Southern hybridization. For verification of the VEL3 deletion and complementation strains, the restriction enzyme XhoI was used and the 5’ region as a probe. For restriction of gDNA from the VOS1 deletion strains, BglI was used and the 3’region severed as probe. (A) Restriction sites of XhoI in the wild type, deletion and complementation strains. The binding site of the 5’ region which was used as probe is marked in blue. (B) Southern hybridization of the VEL3 deletion and the complementation strains with wild type control. The wild type results in a fragment with a size of approximately 2.7 kb. The two tested deletion transformants exhibit fragments with a size of 6.4 kb. The complementation shows a fragment with a size of 2.7 kb. (C) Southern hybridization of the VEL3 deletion and VEL3 and VEL1 as well as VOS1 and VEL3 double deletion strains with wild type control. The wild type results in a fragment of 2.7 kb. Strains containing the VEL3 deletion cassette exhibit a fragment with a size of 6.4 kb. (D) Restriction sites of BglI in the wild type and deletion strains. The 3’ flanking region was used as probe (marked by a blue line). (E) Southern hybridization of VOS1 deletion strains with hygromycin resistance cassette (HYG) with wild type control. The wild type exhibits a fragment with a size of 4.8 kb. The two tested VOS1 deletion transformants show fragments with a size of 1.8 kb. (F) Southern hybridization of the V [file pgen.1009434.s007.TIF]

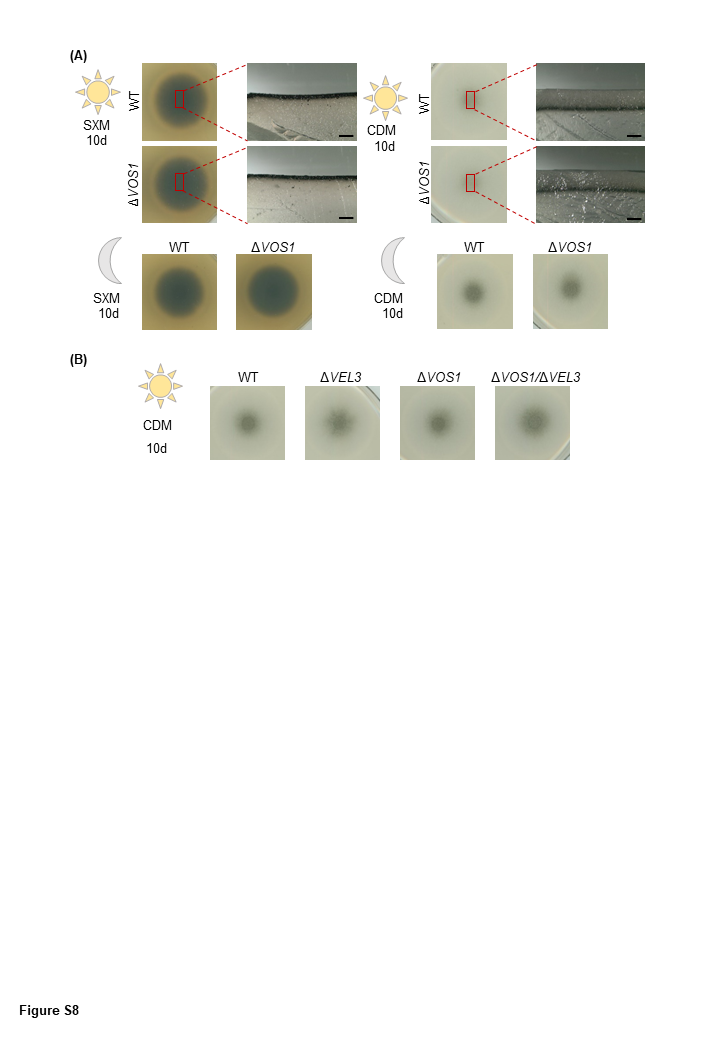

Supplement: S8 Fig — Images give an overview of fungal colonies after spotting 5x10^4^ spores of indicated V. dahliae strains on either simulated xylem medium (SXM) or Czapek-Dox-Medium (CDM) and subsequent incubation for 10 days at 25°C in constant light or darkness. Single colonies on SXM are shown from the back and colonies on CDM are shown from the top of the plate. Cross sections were made through the middle of the colony. (A) Growth and development of the VOS1 deletion (ΔVOS1) and the wild type (WT) strains are similar. Single colonies (left column) and colony cross sections (right column) are shown. (B) A VEL3 and VOS1 double deletion strain (ΔVEL3/ΔVOS1) resembles the phenotype of the VEL3 (ΔVEL3) single deletion strain. (TIF) [file pgen.1009434.s008.TIF]

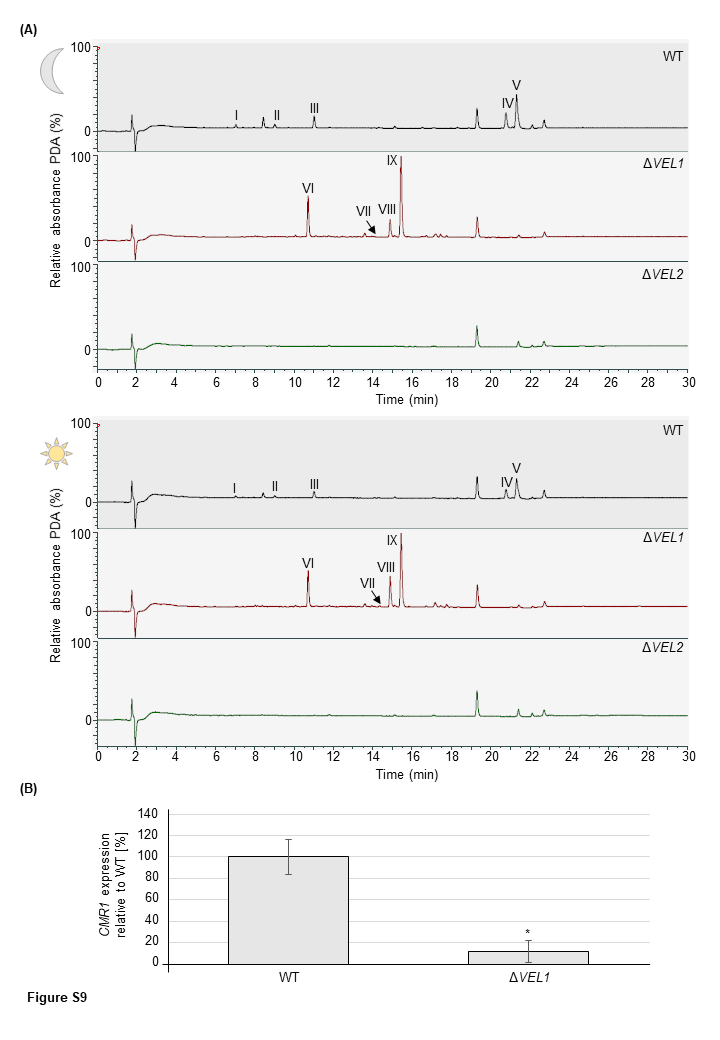

Supplement: S9 Fig — (A) LC/MS combined with photodiode array detection (PDA) analysis of secondary metabolites extracted from the V. dahliae wild type and deletion strains of VEL1 and VEL2 in darkness and light. Secondary metabolites were extracted from two-week-old fungal mycelium grown on Czapek-Dox-Medium (CDM) supplemented with glucose. Chromatograms of the wild type and both deletion strains exhibit the same presence and absence of secondary metabolites in darkness or light. (B) Quantification of CMR1 gene expression in the wild type and the VEL1 deletion strain. Mean values of three independent experiments with standard deviations relative to the wild type are depicted. Normalization was conducted to the reference genes H2A and EIF2B. Significant differences were calculated by t-test and indicate: *:p<0.05. (TIF) [file pgen.1009434.s009.TIF]

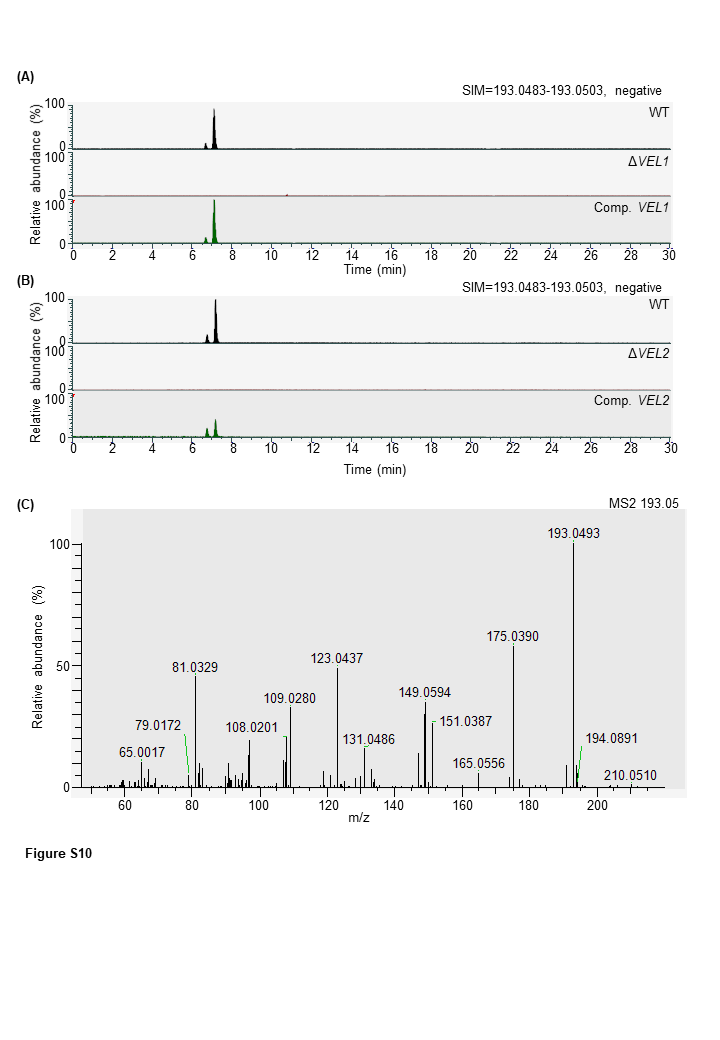

Supplement: S10 Fig — (A) and (B) Single ion monitoring (SIM) for substance I with m/z 193.0493 and a mass tolerance of 5.00 ppm in negative ion mode. Depicted are the wild type (WT), VEL1 deletion strain (ΔVEL1) and complementation (Comp. VEL1) (A) as well as the wild type (WT), VEL2 deletion strain (ΔVEL2) and complementation (Comp. VEL2) (B). (C) MS2 spectrum of substance I. (TIF) [file pgen.1009434.s010.TIF]

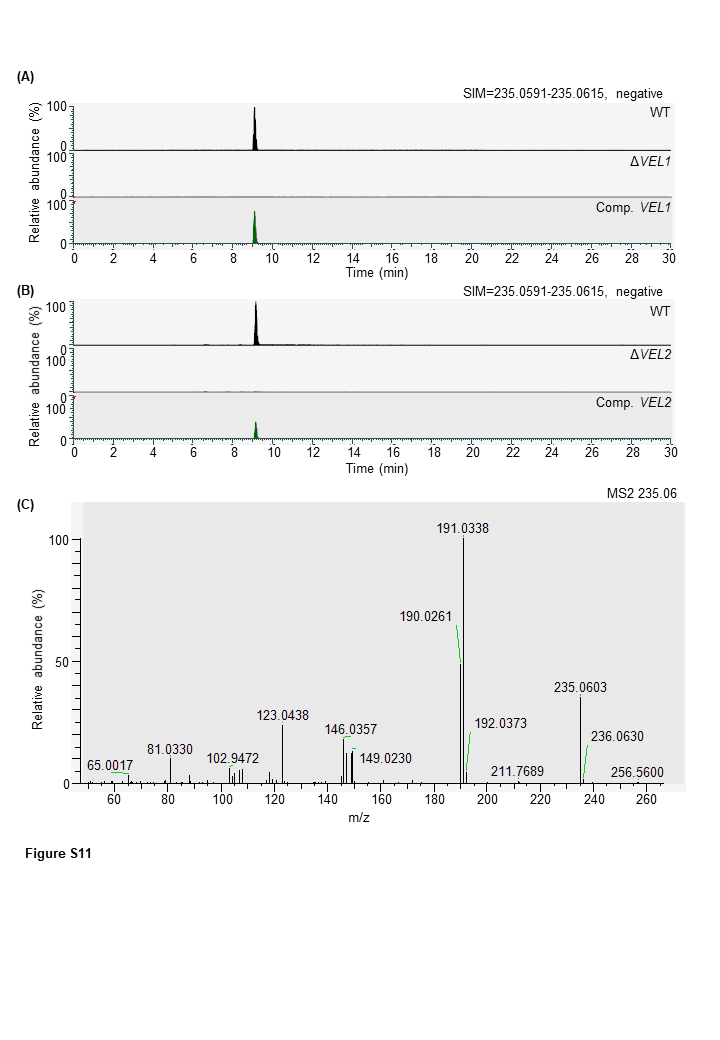

Supplement: S11 Fig — (A) and (B) Single ion monitoring (SIM) for substance II with m/z 235.0603 and a mass tolerance of 5.00 ppm in negative ion mode. Depicted are the wild type (WT), VEL1 deletion strain (ΔVEL1) and complementation (Comp. VEL1) (A) as well as the wild type (WT), VEL2 deletion strain (ΔVEL2) and complementation (Comp. VEL2) (B). (C) MS2 spectrum of substance II. (TIF) [file pgen.1009434.s011.TIF]

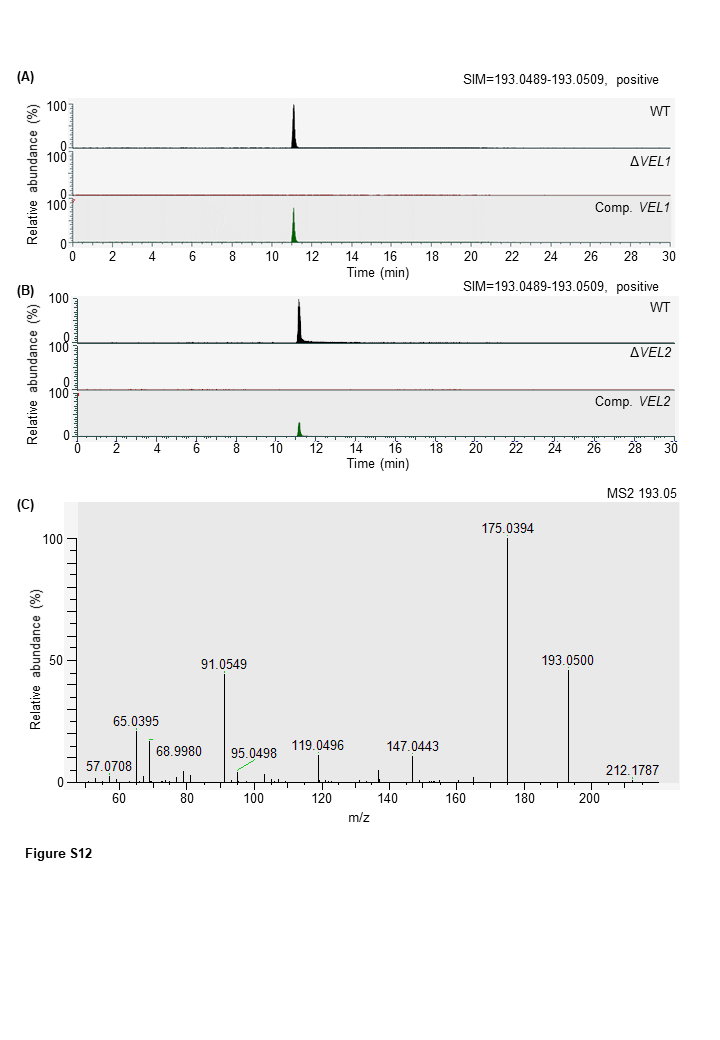

Supplement: S12 Fig — (A) and (B) Single ion monitoring (SIM) for substance III with m/z 193.0499 and a mass tolerance of 5.00 ppm in positive ion mode. Depicted are the wild type (WT), VEL1 deletion strain (ΔVEL1) and complementation (Comp. VEL1) (A) as well as the wild type (WT), VEL2 deletion strain (ΔVEL2) and complementation (Comp. VEL2) (B). (C) MS2 spectrum of substance III. (TIF) [file pgen.1009434.s012.TIF]

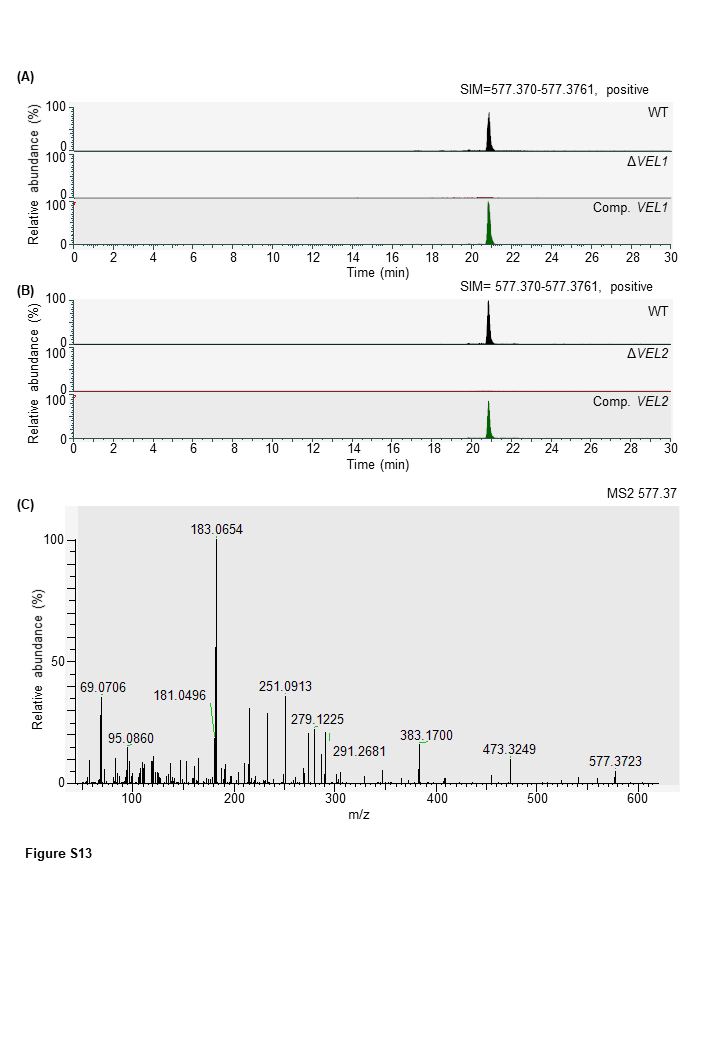

Supplement: S13 Fig — (A) and (B) Single ion monitoring (SIM) for substance IV with m/z 577.3732 and a mass tolerance of 5.00 ppm in positive ion mode. Depicted are the wild type (WT), VEL1 deletion strain (ΔVEL1) and complementation (Comp. VEL1) (A) as well as the wild type (WT), VEL2 deletion strain (ΔVEL2) and complementation (Comp. VEL2) (B). (C) MS2 spectrum of substance IV. (TIF) [file pgen.1009434.s013.TIF]

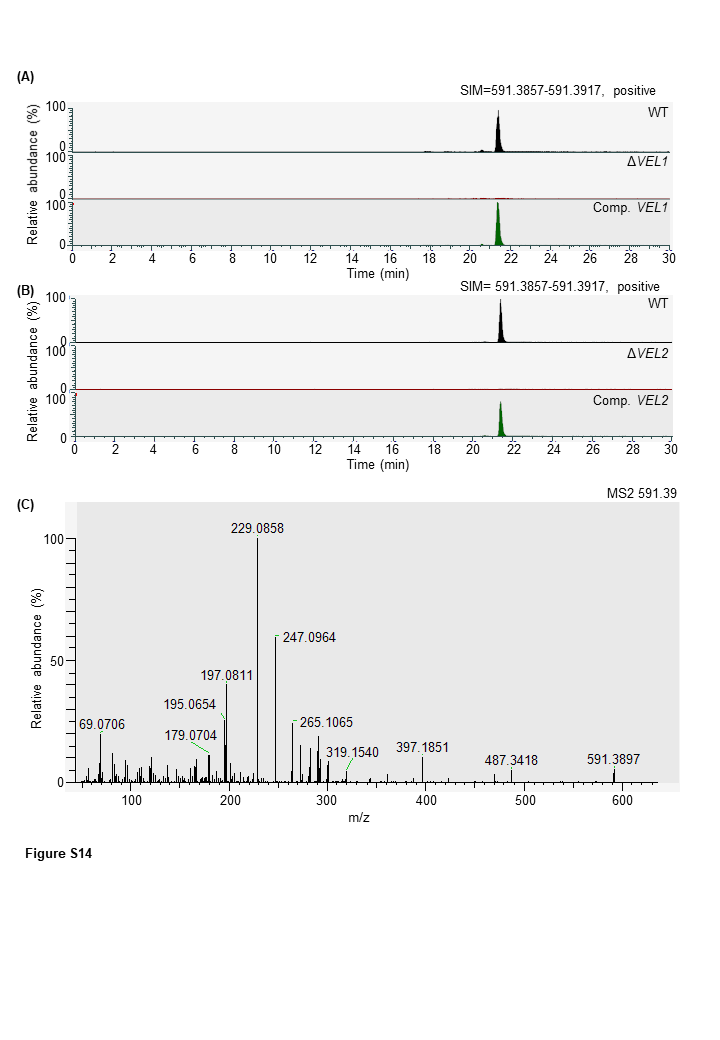

Supplement: S14 Fig — (A) and (B) Single ion monitoring (SIM) for substance V with m/z 591.3887 and a mass tolerance of 5.00 ppm in positive ion mode. Depicted are the wild type (WT), VEL1 deletion strain (ΔVEL1) and complementation (Comp. VEL1) (A) as well as the wild type (WT), VEL2 deletion strain (ΔVEL2) and complementation (Comp. VEL2) (B). (C) MS2 spectrum of substance V. (TIF) [file pgen.1009434.s014.TIF]

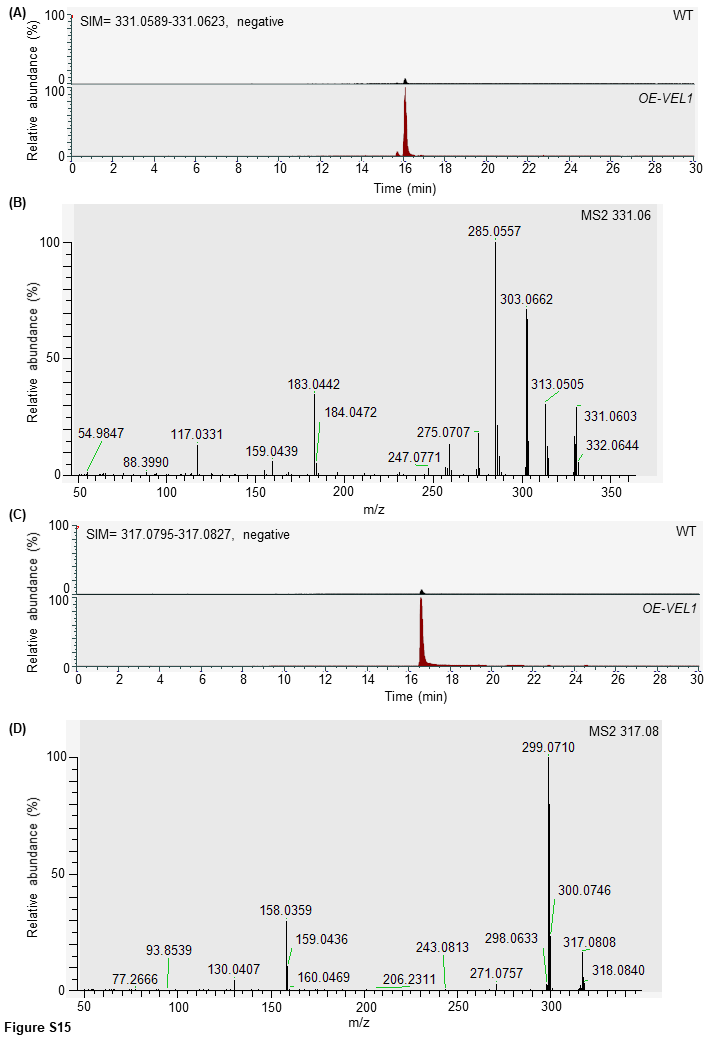

Supplement: S15 Fig — (A) Single ion monitoring (SIM) for substance X with m/z 331.0606 and a mass tolerance of 5.00 ppm in negative ion mode. Depicted are the wild type (WT) and the overexpression of VEL1 (OE-VEL1). (B) MS2 spectrum of substance X. (C) Single ion monitoring (SIM) for substance XI with m/z 317.0811 and a mass tolerance of 5.00 ppm in negative ion mode. Depicted are the wild type (WT) and the overexpression of VEL1 (OE-VEL1). (D) MS2 spectrum of substance XI. (TIF) [file pgen.1009434.s015.TIF]

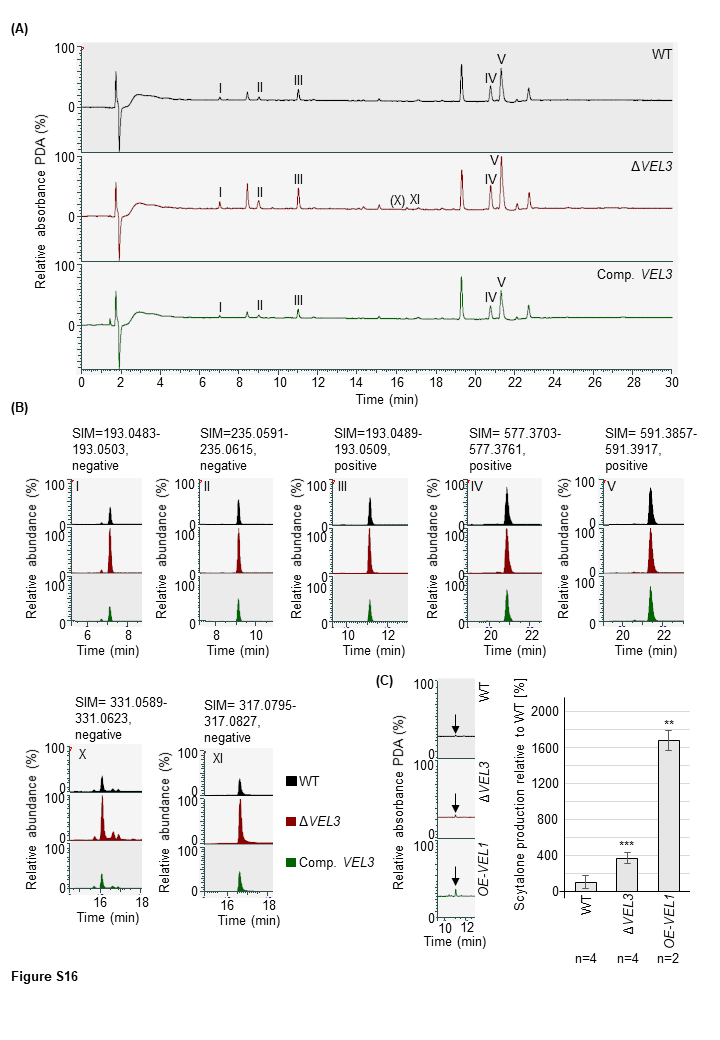

Supplement: S16 Fig — (A) LC/MS combined with photodiode array detection (PDA) analysis of secondary metabolites. Secondary metabolites were extracted from two-week-old fungal mycelium grown on Czapek-Dox-Medium (CDM) supplemented with glucose. Depicted are the wild type (WT), VEL3 deletion strain (ΔVEL3) and a VEL3 complementation strain (Comp. VEL3). All three chromatograms look similar, but increased relative absorbance was observed for the substances I-V in the deletion strain. (B) Selected single ion monitoring (SIM) for the seven substances with increased relative abundance in the VEL3 deletion strain in comparison to wild type and complementation (black: wild type, red: ΔVEL3, green: Comp. VEL3). Substance I: m/z 193.0493, mass tolerance 5.00 ppm; substance II: m/z 235.0603, mass tolerance 5.00 ppm; substance III: m/z 193.0499, mass tolerance 5.00 ppm; substance IV: m/z 577.3722, mass tolerance 5.00 ppm; substance V: m/z 591.3887, mass tolerance 5.00 ppm; substance X: m/z 331.0606, mass tolerance 5.00 ppm; substance XI: m/z 317.0811, mass tolerance 5.00 ppm. (C) Quantification of scytalone production relative to the wild type in the VEL3 deletion (ΔVEL3) strain and the overexpression strain of VEL1 (OE-VEL1). In the left part an example of the scytalone peak is depicted for the wild type, the VEL3 deletion and the VEL1 overexpression strain. In the right part the size of the scytalone peak of at least two independent replicates was quantified relative to the wild type. Significant differences were calculated by t-test and indicate: **:p<0.01; ***:p<0.001. (TIF) [file pgen.1009434.s016.TIF]

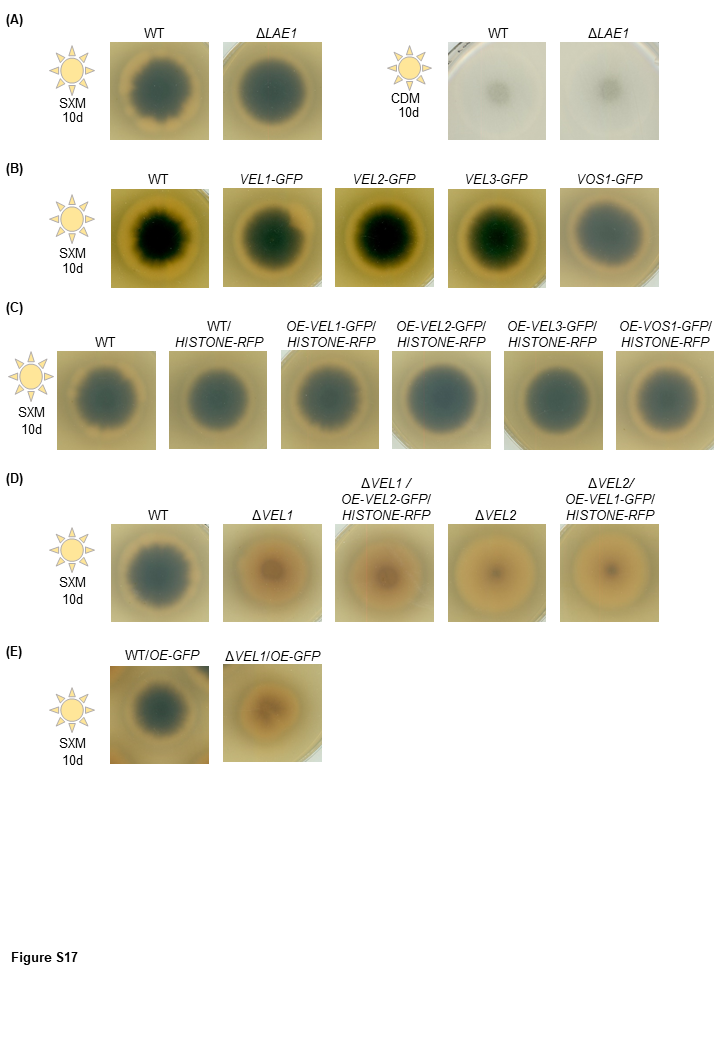

Supplement: S17 Fig — From the indicated strains 5x10^4^ spores were spotted on plates (SXM: simulated xylem medium; CDM: minimal Czapek-Dox-Medium) and incubated for 10 days at 25°C. Single colonies on SXM are shown from the back, colonies on CDM are shown from the top of the plate. Cross sections were made through the middle of the colony. (A) Lae1 is dispensable for microsclerotia formation. The LAE1 deletion strain has a similar phenotype as the wild type when grown on SXM or CDM. (B) Velvet proteins fused C-terminally to GFP under the control of their native promotor show no phenotypic alterations compared to the wild type. (C) Overexpression strains of velvet proteins fused to GFP and transformed with a strain with histone H2B-RFP labeled nuclei have a wild type-like appearance. (D) Overexpression strains of VEL1 and VEL2 fused to GFP and transformed into the deletion strain of VEL2 or VEL1, respectively, have a phenotype similar to the deletion strain. (E) Phenotype of a wild type ectopically expressing high amounts of free GFP and VEL1 deletion strain expressing the same construct. (TIF) [file pgen.1009434.s017.TIF]

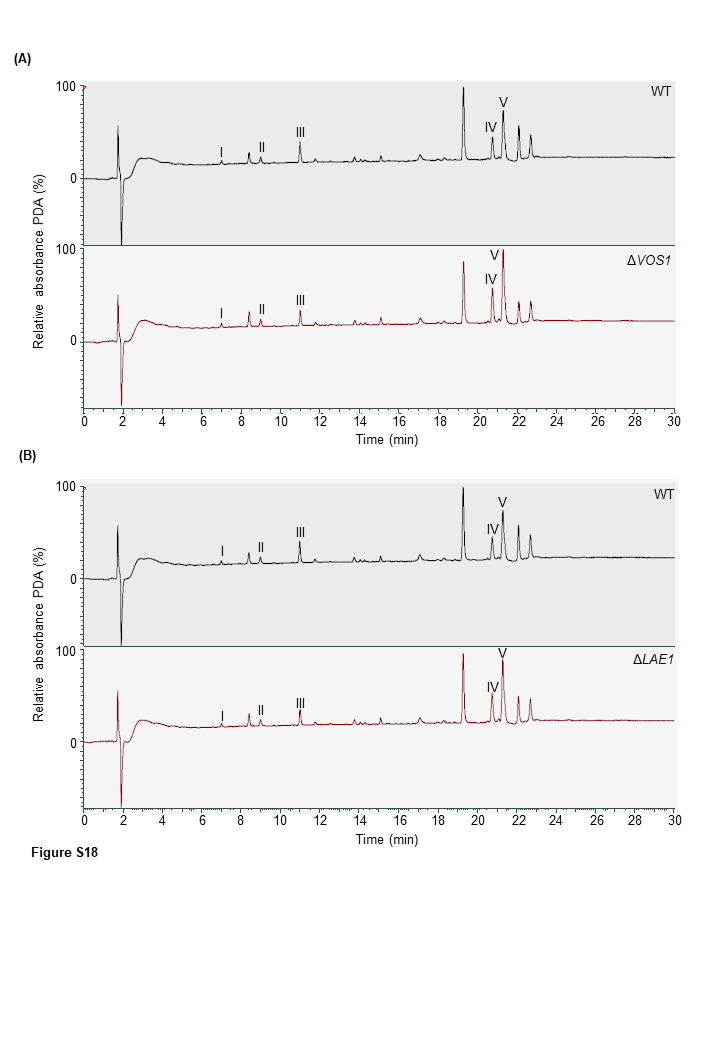

Supplement: S18 Fig — LC/MS combined with photodiode array detection (PDA) analysis of secondary metabolites. Secondary metabolites were extracted from two-week-old fungal mycelium grown on Czapek-Dox-Medium (CDM) supplemented with glucose. Chromatograms of VOS1 and LAE1 deletion strains resemble the wild type. (TIF) [file pgen.1009434.s018.TIF]

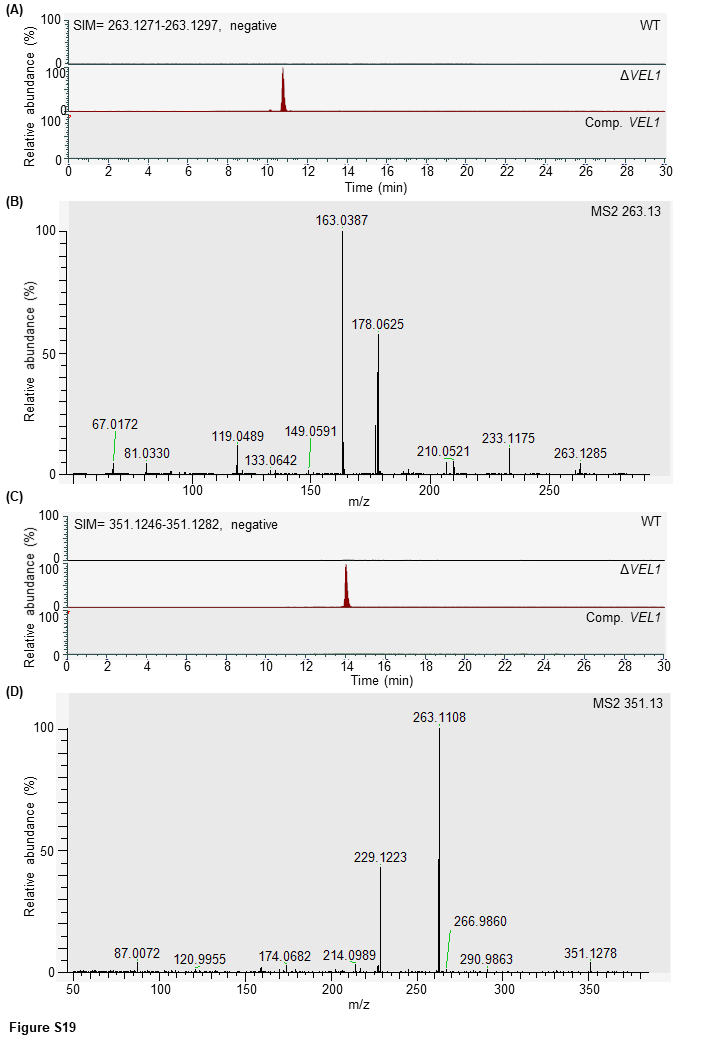

Supplement: S19 Fig — (A) Single ion monitoring (SIM) for substance VI with m/z 263.1284 and a mass tolerance of 5.00 ppm in negative ion mode. Depicted are the wild type (WT), VEL1 deletion strain (ΔVEL1) and complementation (Comp. VEL1). (B) MS2 spectrum of substance VI. (C) Single ion monitoring (SIM) for substance VII with m/z 351.1264 and a mass tolerance of 5.00 ppm in negative ion mode. Depicted are the wild type (WT), VEL1 deletion strain (ΔVEL1) and complementation (Comp. VEL1). (D) MS2 spectrum of substance VII. (TIF) [file pgen.1009434.s019.TIF]

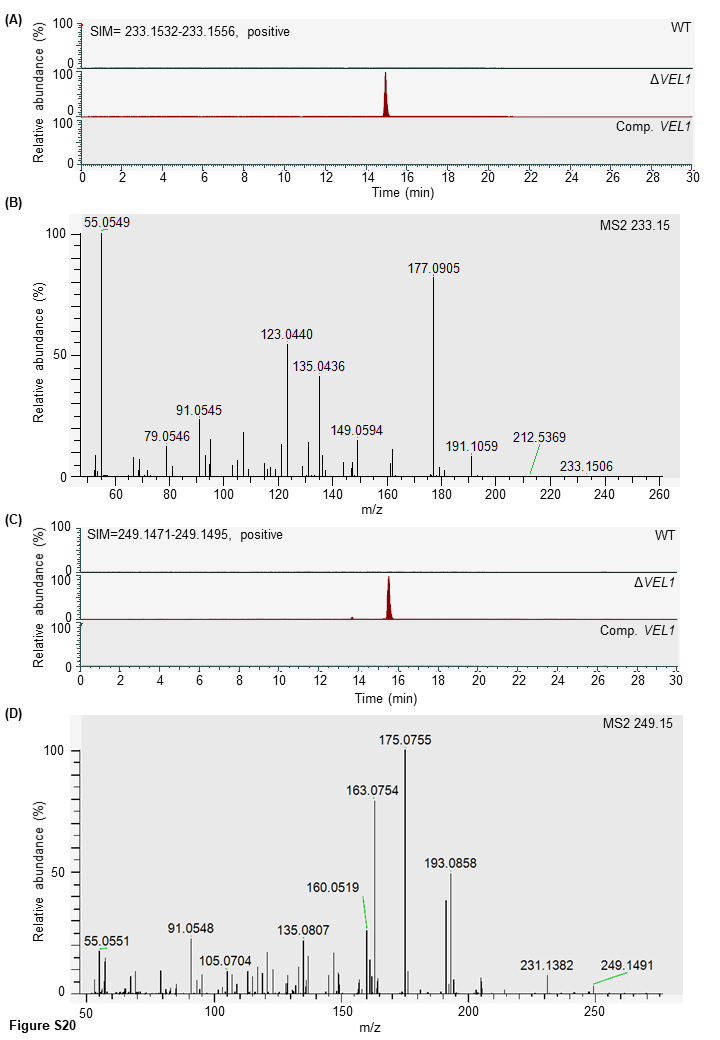

Supplement: S20 Fig — (A) Single ion monitoring (SIM) for substance VIII with m/z 233.1544 and a mass tolerance of 5.00 ppm in positive ion mode. Depicted are the wild type (WT), VEL1 deletion strain (ΔVEL1) and complementation (Comp. VEL1). (B) MS2 spectrum of substance VIII. (C) Single ion monitoring (SIM) for substance IX with m/z 249.1483 and a mass tolerance of 5.00 ppm in positive ion mode. Depicted are the wild type (WT), VEL1 deletion strain (ΔVEL1) and complementation (Comp. VEL1). (D) MS2 spectrum of substance IX. (TIF) [file pgen.1009434.s020.TIF]

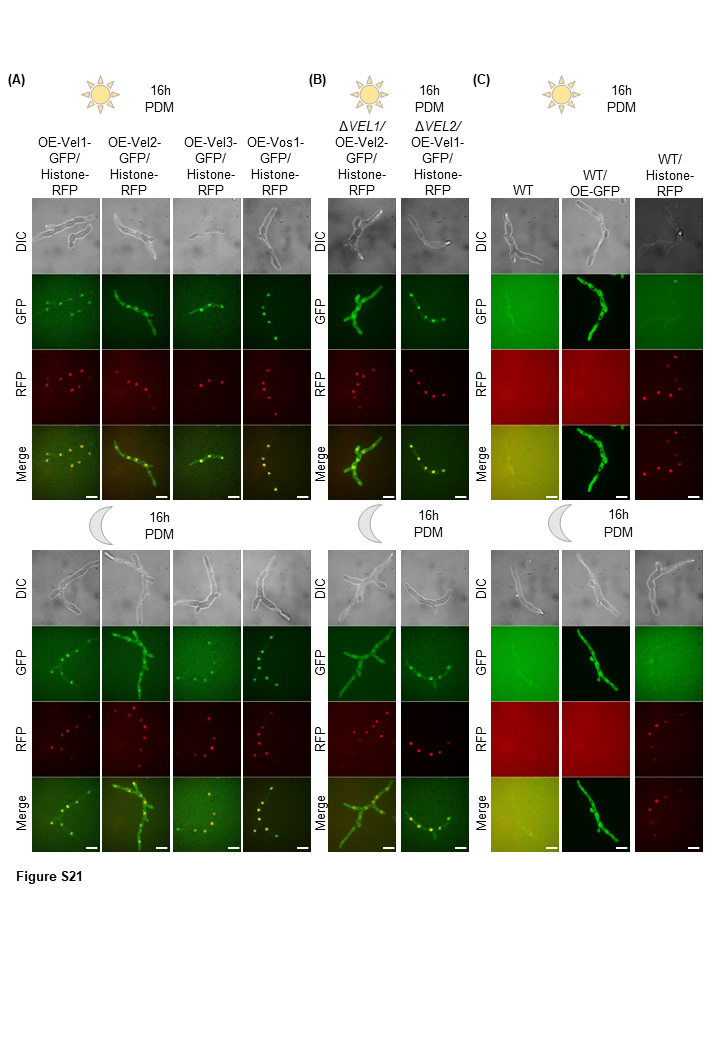

Supplement: S21 Fig — Subcellular localization analysis of fusions of Vel1, Vel2, Vel3 and Vos1 with GFP by fluorescence microscopy. 1x10^4^ freshly harvested spores were incubated in PDM for 16h at 25°C in light or darkness. Differential interference contrast (DIC), green fluorescent filter view (GFP), red fluorescent filter view (RFP) and a merge of GFP and RFP channels are shown. Scale bar = 10 μm. (A) Velvet protein subpopulations are primarily localized to the nucleus in light as well as in darkness with additional small Vel2 subpopulations in the cytoplasm. Nuclei are visualized by histone H2B-RFP. (B) High levels of Vel1 or Vel2 can enter the nucleus independently of each other in light or darkness in ΔVEL1 and ΔVEL2 strains, respectively. (C) Controls for subcellular localization studies in V. dahliae. The wild type (WT) was used as negative control, a strain with overexpressed GFP (WT/OE-GFP) as positive control and a strain in which histone H2B-RFP is overexpressed (WT/HISTONE-RFP) as positive control. (TIF) [file pgen.1009434.s021.TIF]

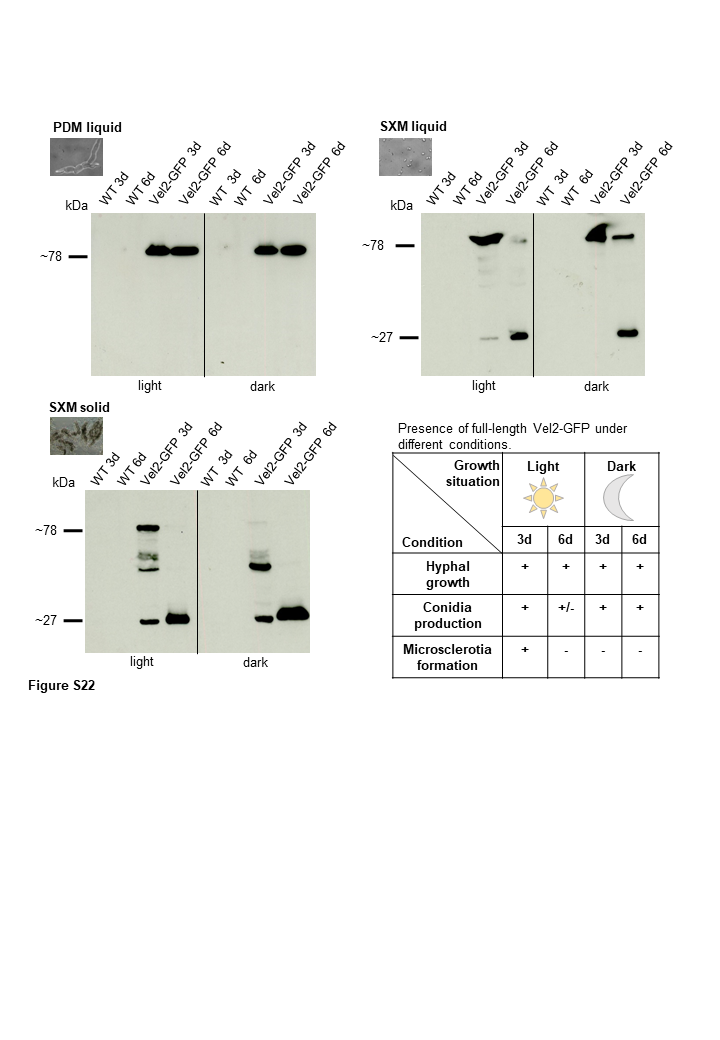

Supplement: S22 Fig — 1x10^6^ freshly harvested spores were inoculated for analysis of protein abundances in liquid potato dextrose medium (PDM) for filamentous growth, in liquid pectin-rich simulated xylem medium (SXM) for conidia formation and on SXM plates covered with a nylon membrane for microsclerotia development. The fungus was grown for three and six days at 25°C in light or darkness. Western hybridization with a GFP antibody was performed with crude extracts of the indicated strains (free GFP: 27 kDa; Vel2-GFP: 78 kDa). Presence (+) or absence (-) of full-length protein during growth in light or darkness during different developmental conditions (hyphal growth in PDM, spore production in liquid SXM, microsclerotia formation on SXM plates) after three and six days. Vel2 is stable during filamentous growth, destabilized during conidiation in light and initially more stable in light than in darkness during microsclerotia formation. (TIF) [file pgen.1009434.s022.TIF]
