# Supplementary material for: The velvet protein Vel1 controls initial plant root colonization and conidia formation for xylem distribution in Verticillium wilt
Source: PLoS Genet. 2021 Mar 15;17(3):e1009434. doi: 10.1371/journal.pgen.1009434 (PMC7993770; doi:10.1371/journal.pgen.1009434)
Supplement: S1 Text — (PDF) [file pgen.1009434.s023.pdf]

Supporting Information Methods

Plasmid and strain constructions

For plasmid construction, the GeneArt Seamless Cloning and Assembly Kit (Thermo Fisher Scientific) was used as indicated by the manufacturer. Alternatively, plasmids were constructed using appropriate restriction enzymes and T4 ligation reactions. Details are given below.

Construction of *VEL1* deletion (*HYG*^{R}) and corresponding complementation strain

For a *VEL1* (VDAG_JR2_Chr7g04890) deletion strain, the 5' and 3' flanking regions of the gene were amplified from wild type gDNA. The 5' region was amplified with the primers AO18 and AO19 (1280 bp) and the 3' region with the primer pair AO20 and AO21 (1185 bp). The hygromycin resistance cassette was obtained from pPK2 [1] using RO3 and RO4 (3942 bp). The backbone was amplified from pME4564 [2] with the primers ML1 and ML2 (6728 bp). All fragments were ligated and resulted in pME5063. This plasmid was used for transformation of the wild type (JR2, [3]). The received deletion transformants were tested by Southern hybridization. Cutting of the genomic DNA obtained from wild type and deletion transformants with *Xho*I and labelling with the 3' flanking region as probe revealed correct integration of the deletion cassette (S6A and B Fig). The two deletion transformants were named VGB246 and VGB247.

For construction of the complementation strain, the *VEL1* 5' flanking region and the gene were amplified by AO101 and AO136 (2998 bp) from gDNA of the wild type. The 3' flanking region was amplified with the primers AO137 and AO138 (1080 bp). The nourseothricin resistance cassette was amplified from pME4815 [4] using ML8 and ML9 (2194 bp). Plasmid pME4564 [2] was treated with *Eco*RV and *Stu*I (6804 bp) and served as backbone. The fragments were ligated and the resulting plasmid was named pME5064. The plasmid was used to transform VGB246. The received strain VGB474 was confirmed by Southern hybridization for correct integration at the locus by using the 3' flanking region as probe and digesting the gDNA with *Xho*I (S6A and B Fig).

Construction of *VEL2* deletion and corresponding complementation strain

For construction of a *VEL2* (VDAG_JR2_Chr3g06150a) deletion strain, the 5' region of *VEL2* was amplified with the primers VEL2-P1 and VEL2-P2 and ligated to *Pac*I/*Spe*I cut vector pME4548 [5]. Next, the vector was cut with *Xba*I/*Sbf*I and ligated to the 3' flanking region, which was amplified by VEL2-P3 and VEL2-P4. The resulting plasmid was named pME5066. Transformation of the wild type (JR2, [3]) with the plasmid resulted in deletion transformants VGB58 and VGB59. The gDNA of the transformants was cut with *Bgl*II and correct integration

of the deletion cassette was confirmed by Southern hybridization using the 3' flanking region as probe (S6D and E Fig).

To construct a corresponding complementation strain with GFP fused to the C-terminus, the primers AO167 and AO168 were used to amplify the 5' flanking region and the *VEL2* gene without stop codon (3550 bp) from wild type gDNA. The 3' flanking region was amplified with the primers AO169 and AO170 (2014 bp). The hygromycin marker was amplified from the plasmid pME4990 [2] with RH590 and RO4 (2641 bp). Also, *GFP* (lacking the start codon) together with a 15 bp flexible linker (protein sequence GGSGG) was amplified from pME4990 by using AO165 and RH514 (747 bp). These two fragments were fused by PCR using the primers AO165 and RO4 (3388 bp). The three fragments were ligated into pME4564 [2] cut with *Eco*RV and *Stu*I (6804 bp). The constructed plasmid was named pME5067. The plasmid was used to transform the deletion strain VGB58. The resulting strain VGB375 was tested by Southern hybridization for correct integration of the complementation cassette at the locus by using the same enzyme and probe as for the deletion (S6D and E Fig).

<u>Construction of *VEL3* deletion and corresponding complementation strain</u>

The *VEL3* (VDAG_JR2_Chr6g00630a) deletion strain was constructed by amplifying the 5' flanking region (AO30 and AO31, 1011 bp) and the 3' flanking region (AO32 and AO33, 1530 bp) from wild type gDNA. The nourseothricin resistance cassette was amplified from pME4815 [4] with the primer pair ML8 and ML9 (2194 bp). The plasmid pME4564 [2] was used to amplify the backbone with the primers ML1 and ML2 (6728 bp). Ligation of the fragments resulted in pME5068, which was used for wild type (JR2, [3]) transformation. The resulting transformants VGB234 and VGB235 were tested for correct integration by Southern hybridization with the 5' flanking region as probe and *Xho*I to treat the gDNA (S7A and B Fig).

To construct the corresponding complementation strain, the 5' flanking region and *VEL3* were amplified from gDNA with the primers AO157 and AO158 (2323 bp). The 3' flanking region was amplified from gDNA with the primers AO159 and AO160 (1548 bp). From the plasmid pPK2 [1] the hygromycin resistance cassette was amplified with the primers RO3 and RO4 (3942 bp). As backbone pME4564 [2] was cut with *Eco*RV and *Stu*I (6804 bp). The fragments were ligated and resulted in the plasmid pME5069. VGB234 was transformed with the construct. The obtained complementation transformants VGB445 and VGB446 were tested by Southern hybridization for correct integration at the locus by using the same enzyme and probe as for the deletion strains (S7A and B Fig).

Construction of *VOS1* (*NAT*^{R}) deletion strain

For construction of a *VOS1* deletion strain with a nourseothricin marker, the *VOS1* 3' flanking region was amplified with the primers VOS1-P3 and VOS1-P4 and inserted into the *Bam*HI/*Hin*dIII restriction site of pKO2 [2]. The 5' flanking region was amplified with the primers VOS1-P1 and VOS1-P2 and ligated into the *Pac*I/*Eco*RV restriction sites. The resulting plasmid was named pME5070. The wild type (JR2, [3]) was transformed with the generated plasmid resulting in deletion transformants VGB64 and VGB65. gDNA of the generated strains was cut with *Bgl*I to test the strains by Southern hybridization using the 3' flanking region as probe (S7D and G Fig).

Construction of *VOS1* (*HYG*^{R}) deletion strain

The *VOS1* deletion strain was also constructed with a hygromycin resistance cassette. Therefore, the 5' flanking region of the gene was amplified from gDNA using AO176 and AO191 (1444 bp). The 3' flanking region was amplified from gDNA with the primers AO178 and AO179 (1210 bp). The hygromycin resistance cassette was amplified from pME5074 with the primers RH590 and RO4 (2641 bp). All fragments were ligated to *Eco*RV and *Stu*I-treated plasmid pME4564 [2] (6804 bp). The resulting plasmid pME5071 was used for transformation of the wild type. The received transformants VGB241 and VGB242 were verified by Southern hybridization of gDNA cut with *Bgl*I and the 3' flanking region as probe (S7D and E Fig).

Construction of *VEL1*/*VEL2* double deletion strain

To obtain a double deletion strain of *VEL1* and *VEL2*, the single deletion strain of *VEL2* (VGB58) was used as parental strain. The plasmid pME5063 was used to transform VGB58. The resulting double deletion transformants VGB281 and VGB282 were confirmed by Southern hybridization in the same way as the single deletion strains (S6 A and C, D and F Fig).

Construction of *VEL3*/*VEL1* double deletion strain

For construction of a *VEL3* and *VEL1* double deletion strain, the *VEL1* single deletion strain (VGB246) was transformed with pME5068. The resulting *VEL1* and *VEL3* double deletion transformants VGB289 and VGB290 were tested by Southern hybridization with the same enzyme and probe as the single deletion strains (S6A and C, S7A and C Figs).

Construction of VOS1/VEL3 double deletion strain

A *VOS1* and *VEL3* double deletion strain was constructed by using the single deletion strain of *VEL3* (VGB234) as parental strain. The plasmid pME5071 was used for transformation of the *VEL3* deletion strain resulting in VGB373. Southern hybridization was conducted as for the

VOS1 and *VEL3* single deletion strains to confirm the double deletion strain (S7A and C, D and F Fig).

Construction of *VEL1* deletion strain overexpressing *GFP*

For examination of root colonization, the plasmid pME4819 [4] was introduced into the *VEL1* deletion strain (VGB246). Resulting transformants were named VGB443 and VGB444. A screening for green fluorescence was conducted in 15 µ-Slide 8 Well microscopy chambers (Ibidi). Additionally, the strains were tested by phenotypical analysis and Southern hybridization for correct integration of the deletion cassette.

Construction of a strain with *VEL1-GFP* at the endogenous locus

A strain coding for Vel1 C-terminally fused to GFP was constructed by amplifying the *VEL1* 5' flanking region and the gene without stop codon with the primer pair AO101 and AO166 (2995 bp) from gDNA. The 3' flanking region of *VEL1* was amplified from gDNA with AO140 and AO138 (1080 bp). The plasmid pME4990 [2] was used to amplify the hygromycin resistance marker with the primers RH590 and RO4 (2641 bp). Furthermore, *GFP* (without start codon) and a 15 bp flexible linker (protein sequence GGSGG) were amplified from the same plasmid with AO165 and RH514 (747 bp). In a fusion PCR the hygromycin resistance cassette and *GFP* were linked by using AO165 and RO4 (3388 bp). pME4564 [2] cut with *Eco*RV and *Stu*I was used as backbone (6804 bp). The constructed plasmid was named pME5072. The plasmid was used to transform the wild type (JR2, [3]) resulting in VGB447. gDNA of the strain was cut with *Bam*HI and Southern hybridization was performed to confirm correct integration at the locus with the 3' flanking region as probe. The presence of the fusion protein was tested by western analysis (Fig 5A). The strain was additionally examined phenotypically for wild type-like functionality.

Construction of a strain with *VEL2-GFP* at the endogenous locus

To construct a *VEL2* strain expressing *GFP* at the endogenous locus, the wild type (JR2, [3]) was transformed with plasmid pME5067. The resulting strain VGB450 was tested for correct integration at the locus by Southern hybridization. Therefore, gDNA was cut with *Hinc*II and the 5' flanking region was used as probe. The presence of the fusion protein was confirmed by western experiments (Fig 5A). The strain was also checked by phenotypical analysis for wild type-like functionality.

Construction of a strain with *VEL3-GFP* at the endogenous locus

For construction of a strain coding for Vel3 C-terminally fused to GFP, the primers AO157 and AO171 were used to amplify the *VEL1* 5'flanking region and the gene without stop codon (2323 bp) from gDNA. The 3' flanking region of *VEL3* was amplified with AO159 and AO160 (1548

bp) from gDNA. The hygromycin resistance cassette was amplified from pME4990 [2] with the primers RH590 and RO4 (2641 bp). *GFP* (lacking the start codon) and a 15 bp flexible linker (protein sequence GGSGG) were amplified from pME4990 [2] using AO165 and RH514 (747 bp). These two fragments were fused by PCR with AO165 and RO4 (3388 bp). The plasmid pME4564 [2] was cut with *Eco*RV and *Stu*I (6804 bp) and used as backbone. The fragments were ligated and the resulting plasmid was named pME5073. The wild type (JR2, [3]) was transformed with the plasmid resulting in VGB451. gDNA of the constructed strain was cut with *Pst*I and confirmed by Southern hybridization for integration at the locus with the 3' flanking region as probe. Furthermore, the strain was tested by western experiments for presence of the fusion protein (Fig 5A) and phenotypically analysed for wild type-like functionality.

Construction of a strain with *VOS1-GFP* at the endogenous locus

In order to construct a strain coding for Vos1 C-terminally fused to GFP, the *VOS1* 5' flanking region and *VOS1* without stop codon were amplified with the primer pair AO176 and AO177 (2683 bp) from gDNA. For amplification of the 3' flanking region of the gene from gDNA, AO178 and AO179 were used (1210 bp). To amplify *GFP* (without start codon) together with a 15 bp flexible linker (protein sequence GGSGG) from the plasmid pME4990 [2], the primers AO165 and RH514 were used (747 bp). From the same fragment also the hygromycin resistance cassette was amplified with RH590 and RO4 (2641 bp). Next, the two fragments were fused by PCR using AO165 and RO4 (3388 bp). The plasmid pME4564 [2] was cut with *Eco*RV and *Stu*I to obtain the backbone (6804 bp). The received plasmid was named pME5074. The plasmid was used to transform the wild type (JR2, [3]) resulting in VGB453. The strain was tested by Southern hybridization. Therefore, gDNA was cut with *Pst*I and *Xho*I and tested for correct integration using the 3' flanking region as probe. Furthermore, the gDNA was digested with *Aat*II and tested by Southern hybridization with the 5' flanking region as probe. The strain was also verified by western experiments (Fig 5A) and phenotypical analysis for wild type-like functionality.

Construction of a strain with Histone-*RFP* fluorescence

In order to construct a strain with red fluorescent nuclei, the histone H2B was tagged with *RFP* in a plasmid named pME4975 [4]. The wild type (JR2, [3]) was transformed with the plasmid and the resulting strain was named VGB22.

Construction of *VEL1-GFP* overexpression strain

For the construction of a strain overexpressing *VEL1* fused to *GFP*, the primers ML30 and ML31 were used to amplify the backbone of pGreen2 [6] including *GFP*. *VEL1* was amplified from cDNA with the primer pair AO15 and AO14 (1686 bp) without stop codon. The resulting plasmid was named pME5078. In the next step, pPK2 [1] was cut with *Eco*RV and the *GPDA*

promoter, *VEL1-GFP* and the *TRPC* terminator were amplified from pME5078 with PC4 and ML8 (4049 bp). The PCR fragment was ligated into pPK2 [1] resulting in pME5079. The wild type (JR2, [3]) was transformed with pME5079 resulting in VGB297. This strain was confirmed by western hybridization for the presence of Vel1-GFP. Furthermore, VGB22 was transformed with pME5079 for localization studies. The resulting transformants were named VGB364 and VGB365 and were checked for presence of the fusion protein by western analysis.

Construction of *VEL2-GFP* overexpression strain

To construct a strain overexpressing *VEL2* with GFP fused to its C-terminus, the plasmid pGreen2 [6] was used as backbone. *VEL2* was amplified from cDNA without stop codon using VEL2gfp-F and VEL2gfp-R (1380 bp) and inserted into the vector at the *Xho*I/*Kpn*I restriction site. The constructed plasmid was named pME5080. For localization studies, VGB22 was transformed with pME5080. The resulting strain was named VGB223 and was checked by western analysis for presence of the fusion protein.

Construction of *VEL3-GFP* overexpression strain

The construction of a *VEL3* overexpression strain C-terminally fused to GFP was conducted in several steps. In the first step, the primers AO28 and AO29 were used to amplify *VEL3* from cDNA (1341 bp) without stop codon. The primers ML30 and ML31 were used to amplify the backbone including *GFP* with a 15 bp flexible linker (protein sequence GGSGG) from pGreen2 [6]. The fragment was ligated into the backbone and the resulting fragment was named pME5081. The primers ZQY3 and ZQY7 were used to amplify the *GPDA* promoter, *VEL3-GFP* and the *TRPC* terminator from pME5081 (3734 bp). In the next step, pBlueScript II KS (Fermentas) was treated with *Eco*RV and ligated with the previously generated fragment. The resulting construct was named pME5082. Lastly, pPK2 [1] was linearized with *Eco*RV and the primers ZQY8 and ZQY9 were used to amplify the *GPDA* promoter, *VEL3-GFP* and the *TRPC* terminator from pME5082 (3704 bp). The fragments were ligated and the resulting plasmid was named pME5083. To examine the localization of *VEL3*, pME5083 was used to transform VGB22. The generated strains were named VGB501 and VGB502. Western analysis was conducted to check the presence of the fusion protein.

Construction of *VOS1-GFP* overexpression strain

In order to construct an overexpression strain of *VOS1* fused C-terminally with *GFP*, the primers Vos1orf-E1 and Vos1orf-E2 were used to amplify *VOS1* from cDNA (1137 bp) without stop codon. The plasmid pGreen2 [6] was treated with *Xho*I/*Kpn*I to serve as backbone and was ligated to *VOS1*. The resulting plasmid was named pME5084. VGB22 was transformed with pME5084 for localization studies. The resulting transformants were named VGB219 and VGB220. Western analysis was conducted to check the presence of the fusion protein.

Construction of a *VEL1-GFP* overexpression strain with *VEL2* deletion and Histone *RFP* expression

For the construction of a *VEL1-GFP* overexpression strain in the *VEL2* deletion background, the plasmid pME5079 was used to transform the deletion strain of *VEL2* (VGB58). The resulting strain was named VGB362. In the next step, VGB362 was transformed with pME4976 [4] to generate a strain with red fluorescent nuclei for localization studies. The resulting transformants VGB498 and VGB499 were verified by Southern hybridization. The presence of the Vel1-GFP fusion protein was confirmed via western analysis.

Construction of a *VEL2-GFP* overexpression strain with *VEL1* deletion and Histone *RFP* expression

For construction of a *VEL2-GFP* overexpression strain in the VEL1 deletion, the *VEL1* deletion was constructed with a nourseothricin resistance cassette. Therefore, the 5' flanking region of *VEL1* was amplified with AO101 and AO175 (1280 bp) and the 3' flanking region with the primers AO140 and AO174 (1185 bp) from wild type gDNA. The nourseothricin resistance marker was amplified from pME4815 [4] with ML8 and ML9 (2194 bp). As backbone pME4564 [2] was cut with *Eco*RV and *Stu*I (6804 bp). The fragments were ligated and the resulting plasmid was named pME5065. VGB477 [4] was transformed with the plasmid resulting in VGB495. Next, VGB495 was transformed with pME5080 leading to VGB496 and VGB497. The strains were tested by Southern hybridization for correct integration of the deletion cassette. The presence of the fusion protein was confirmed by western analysis.

Chloroform-Methanol extraction of proteins

The eluted proteins (E1-E3) were directly applied to chloroform-methanol extraction to remove detergents and salts. The protocol was modified according to Wessel & Flügge [7]. Elution fractions were adjusted to 100 µl and vigorously mixed with 400 µl methanol. After a short centrifugation step for 10 sec at 10000 rpm, 100 µl chloroform were added, mixed and the centrifugation step was repeated. Next, 300 µl H_2O (HPLC grade) were added and vigorously mixed with the solution. To achieve two phases, the samples were centrifuged for 3 min at 10000 rpm at 4 °C. The upper phase was discarded without destroying the interphase. After adding 300 µl methanol to the lower phase and vigorously mixing the solution, the samples were centrifuged for 10 min at 4 °C and 13000 rpm. The supernatant was removed, the protein sediments were dried in a SpeedVac Concentrator (Thermo Fisher Scientific Schwerte) at 50 °C and stored at -20 °C.

In solution digestion and peptide purification

Proteins were resuspended in *Rapi*GestSF Surfactant as indicated by the manufacturer (Waters) for enhanced protein digestion. At this step, all elution fractions were pooled. The proteins were digested with trypsin (Serva, final dilution 1:20) overnight. Trifluoroacetic acid (TFA) was added to the digested peptides as recommended by the manufacturer (Waters). Samples were dried in a SpeedVac Concentrator and purified by using StageTips [8,9]. Dried samples were stored at -20 °C.

Liquid chromatography/ mass spectrometry (LC/MS) analysis of peptides

Dried peptide samples were reconstituted in 20 µl LC/MS sample buffer (2 % acetonitrile, 0.1 % formic acid). From each sample 2 µl were applied to reverse phase liquid chromatography for peptide separation by an RSLCnano Ultimate 3000 system (Thermo Fisher Scientific). For this purpose, the peptides were loaded on an Acclaim PepMap 100 pre-column (100 µm x 2 cm, C18, 5 µm, 100 Å; Thermo Fisher Scientific) with 0.07 % trifluoroacetic acid at a flow rate of 20 µL/min for 3 min. On an Acclaim PepMap RSLC column (75 µm x 50 cm, C18, 2 µm, 100 Å; Thermo Fisher Scientific) analytical separation of peptides was done with a flow rate of 300 nL/min. Composition of the solvent was gradually changed within 94 min from 96 % solvent A (0.1 % formic acid) and 4 % solvent B (80 % acetonitrile, 0.1 % formic acid) to 10 % solvent B within 2 min, to 30 % solvent B within the next 58 min, to 45 % solvent B within the following 22 min, and to 90 % solvent B within the last 12 min of the gradient. Used solvents and acids had Optima grade for LC/MS (Thermo Fisher Scientific). By nano-electrospray (nESI) the eluting peptides were on-line ionized with the Nanospray Flex Ion Source (Thermo Fisher Scientific) at 1.5 kV (liquid junction) and transferred into a Q Exactive HF mass spectrometer (Thermo Fisher Scientific). In a mass range of 300 to 1650 m/z full scans were recorded at a resolution of 30,000 followed by data-dependent top 10 HCD fragmentation at a resolution of 15,000 (dynamic exclusion enabled). The XCalibur 4.0 software (Thermo Fisher Scientific) was used for LC/MS method programming and data acquisition.

LC/MS data was analyzed by MaxQuant 1.6.0.16 [10] and Perseus 1.6.0.7 [11]. MaxQuant was used with default parameters and label free quantification (LFQ) to analyse the raw data. The protein database from EnsemblFungi [12] was used in MaxQuant 1.6.0.16 but the entry of VDAG_JR2_Chr31209a was altered as indicated (S4 and S5E Figs). Obtained data was evaluated with Perseus 1.6.0.7 Data was processed as indicated in S12 Table.

Supporting Information References

1. Covert SF, Kapoor P, Lee M, Briley A, Nairn CJ. *Agrobacterium tumefaciens*-mediated transformation of *Fusarium circinatum*. Mycol Res. 2001;105:259–264.

2. Leonard M, Kühn A, Harting R, Maurus I, Nagel A, Starke J, et al. *Verticillium longisporum* elicits media-dependent secretome responses with capacity to distinguish between plant-related environments. Front Microbiol. 2020;11:1876.

3. Fradin EF, Zhang Z, Juarez Ayala JC, Castroverde CDM, Nazar RN, Robb J, et al. Genetic dissection of Verticillium wilt resistance mediated by tomato Ve1. Plant Physiol. 2009;150:320–332.

4. Starke J, Harting R, Maurus I, Bremenkamp R, James W. Unfolded protein response and scaffold independent pheromone MAP kinase signalling control *Verticillium dahliae* growth, development and plant pathogenesis. bioRxiv. 2020;doi: 10.1101/2020.02.10.941450.

5. Bui T, Harting R, Braus-Stromeyer SA, Tran V, Leonard M, Höfer A, et al. *Verticillium dahliae* transcription factors Som1 and Vta3 control microsclerotia formation and sequential steps of plant root penetration and colonisation to induce disease. New Phytol. 2019;221:2138–2159.

6. Tran V-TT, Braus-Stromeyer SA, Kusch H, Reusche M, Kaever A, Kühn A, et al. Verticillium transcription activator of adhesion Vta2 suppresses microsclerotia formation and is required for systemic infection of plant roots. New Phytol. 2014;202:565–581.

7. Wessel D, Flügge UI. A method for the quantitative recovery of protein in dilute solution in the presence of detergents and lipids. Anal Biochem. 1984;138:141–143.

8. Rappsilber J, Ishihama Y, Mann M. Stop And Go Extraction tips for matrix-assisted laser desorption/ionization, nanoelectrospray, and LC/MS sample pretreatment in proteomics. Anal Chem. 2003;75:663–670.

9. Rappsilber J, Mann M, Ishihama Y. Protocol for micro-purification, enrichment, pre-fractionation and storage of peptides for proteomics using StageTips. Nat Protoc. 2007;2:1896–1906.

10. Cox J, Mann M. MaxQuant enables high peptide identification rates, individualized p.p.b.-range mass accuracies and proteome-wide protein quantification. Nat Biotechnol. 2008;26:1367–1372.

11. Tyanova S, Temu T, Sinitcyn P, Carlson A, Hein MY, Geiger T, et al. The Perseus computational platform for comprehensive analysis of (prote)omics data. Nat Methods. 2016;13:731–740.

12. Kersey PJ, Allen JE, Allot A, Barba M, Boddu S, Bolt BJ, et al. Ensembl Genomes 2018: An integrated omics infrastructure for non-vertebrate species. Nucleic Acids Res. 2018;46:D802-D808.
