# Supplementary material for: The velvet protein Vel1 controls initial plant root colonization and conidia formation for xylem distribution in Verticillium wilt
Source: PLoS Genet. 2021 Mar 15;17(3):e1009434. doi: 10.1371/journal.pgen.1009434 (PMC7993770; doi:10.1371/journal.pgen.1009434)
Supplement: S1 Table — (PDF) [file pgen.1009434.s025.pdf]

S1 Table. Proteins significantly enriched with Vel1-GFP and their predicted domains and functions. During data analysis the command "Replace missing values from normal distribution" was repeated four times. Proteins enriched in all four repetitions are displayed as "Found in 4/4", proteins found in three repetitions are displayed as "Found in 3/4".

	Protein ID	Predicted domain	Potential function
Found in 4/4	VDAG_JR2_Chr7g00220a-00001	Cyclophilin-type peptidyl-prolyl cis-trans isomerase domain	Protein folding
	VDAG_JR2_Chr7g04890a-00001(Vel1)	Velvet domain	Development, protein binding
	VDAG_JR2_Chr7g05280a-00001	FAD/NAD(P)-binding domain	Redox metabolism
	VDAG_JR2_Chr3g06150a-00001(Vel2)	Velvet domain	Development, protein binding
	VDAG_JR2_Chr5g09190a-00001	Tetratricopeptide-like helical domain superfamily	Protein binding
	VDAG_JR2_Chr6g10140a-00001	Haem peroxidase	Redox metabolism, stress response
	VDAG_JR2_Chr3g09450a-00001	NAD-dependent epimerase/dehydratase	Epimerase/Dehydratase
Found in 3/4	VDAG_JR2_Chr8g02960a-00001	Aminotransferase	Amino acid metablosim
	VDAG_JR2_Chr5g05440a-00001	Glycoside hydrolase, family 13	Carbohydrate metabolism
	VDAG_JR2_Chr1g28705a-00001	RNA recognition motif domain	RNA binding
	VDAG_JR2_Chr3g02760a-00001	Peptidase family M49	Proteolysis
