# Supplementary material for: The velvet protein Vel1 controls initial plant root colonization and conidia formation for xylem distribution in Verticillium wilt
Source: PLoS Genet. 2021 Mar 15;17(3):e1009434. doi: 10.1371/journal.pgen.1009434 (PMC7993770; doi:10.1371/journal.pgen.1009434)
Supplement: S2 Table — (PDF) [file pgen.1009434.s026.pdf]

S2 Table. Proteins significantly enriched with Vel2-GFP and their predicted domains and functions. During data analysis the command "Replace missing values from normal distribution" was repeated four times. Proteins enriched in all four repetitions are displayed as "Found in 4/4", proteins found in three repetitions are displayed as "Found in 3/4".

	Protein ID	Predicted domain	Potential function
Found in 4/4	VDAG_JR2_Chr3g06150a-00001(Vel2)	Velvet domain	Development, protein binding
	VDAG_JR2_Chr5g03080a-00001	Protein of unknown function DUF3759	
	VDAG_JR2_Chr4g06160a-00001	Citrate synthase	Carbohydrate metabolism
	VDAG_JR2_Chr4g02640a-00001	Alcohol dehydrogenase	Redox metabolism
	VDAG_JR2_Chr4g06150a-00001	ATP-citrate synthase, citrate-binding domain	Carbohydrate metabolism
	VDAG_JR2_Chr7g00220a-00001	Cyclophilin-type peptidyl-prolyl cis-trans isomerase domain	Protein folding
	VDAG_JR2_Chr8g05200a-00001	Thioredoxin domain	Redox metabolism
	VDAG_JR2_Chr5g10680a-00001	NAD(P)-binding domain superfamily, NmrA-like domain	
	VDAG_JR2_Chr2g02650a-00001	Xylulose 5-phosphate/Fructose 6-phosphate phosphoketolase	Carbohydrate metabolism
	VDAG_JR2_Chr5g01680a-00001	Secretion signal	Extracellular protein
	VDAG_JR2_Chr1g20590a-00001	Acyl transferase	Fatty acid metabolism
	VDAG_JR2_Chr6g02720a-00001	Acetyl-CoA carboxylase	Fatty acid metabolism
	VDAG_JR2_Chr6g01710a-00001	NAD(P)-binding domain superfamily; Short-chain dehydrogenase/reductase SDR	Redox metabolism
	VDAG_JR2_Chr1g20610a-00001	Fatty acid synthase subunit alpha, acyl carrier domain	Fatty acid metabolism
	VDAG_JR2_Chr2g07360a-00001	Pyridoxal phosphate-dependent decarboxylase	Amino acid metabolism
	VDAG_JR2_Chr7g04890a-00001(Vel1)	Velvet domain	Development, protein binding
	VDAG_JR2_Chr1g13580a-00001	Glutathione-dependent formaldehyde-activating enzyme/centromere protein V	Detoxification
	VDAG_JR2_Chr4g10440a-00001	RNA recognition motif domain	Nucleic acid binding
	VDAG_JR2_Chr7g05280a-00001	FAD/NAD(P)-binding domain	Redox metabolism
	VDAG_JR2_Chr8g10760a-00001	NADP-dependent oxidoreductase domain	Redox metabolism
	VDAG_JR2_Chr8g08780a-00001	Alpha-D-phosphohexomutase	Carbohydrate metabolism
	VDAG_JR2_Chr8g02960a-00001	Aminotransferase	Amino acid metabolism
	VDAG_JR2_Chr8g04960a-00001	UTP-glucose-1-phosphate uridylyltransferase	Carbohydrate metabolism
	VDAG_JR2_Chr1g18400a-00001	Glutathione S-transferase	Redox metabolism, protein binding
	VDAG_JR2_Chr4g07720a-00001	Alcohol dehydrogenase	Redox metabolism
	VDAG_JR2_Chr1g11550a-00001	Haloacid dehalogenase-like hydrolase	Hydrolase activity
	VDAG_JR2_Chr6g06890a-00001	Melanoma-associated antigen	
	VDAG_JR2_Chr7g08750a-00001		
	VDAG_JR2_Chr4g08190a-00001	Fatty acid desaturase domain	Fatty acid metabolism
	VDAG_JR2_Chr6g10140a-00001	Haem peroxidase	Redox metabolism, stress response
	VDAG_JR2_Chr5g09190a-00001	Tetratricopeptide-like helical domain superfamily	Protein binding
	VDAG_JR2_Chr1g17980a-00001	Peptidase S8 propeptide/proteinase inhibitor I9	Protein folding
	VDAG_JR2_Chr4g02180a-00001	NAD(P)-binding domain	
	VDAG_JR2_Chr2g08140a-00001	Peptidase C1B, bleomycin hydrolase	Proteolysis
	VDAG_JR2_Chr3g03700a-00001	Sugar isomerase	Carbohydrate metabolism
	VDAG_JR2_Chr5g05440a-00001	Glycoside hydrolase, family 13	Carbohydrate metabolism
	VDAG_JR2_Chr7g00600a-00001	Thioredoxin domain	Redox metabolism
	VDAG_JR2_Chr4g07360a-00001	NADP-dependent oxidoreductase domain	Redox metabolism
	VDAG_JR2_Chr3g10860a-00001	K Homology domain, type 1	Nucleic acid binding
	VDAG_JR2_Chr3g01680a-00001	TspO/MBR-related protein	Transmembrane signalling
	VDAG_JR2_Chr3g12090a-00001(Vos1)	Velvet domain	Development, protein binding
	VDAG_JR2_Chr4g08010a-00001	Class I glutamine amidotransferase-like	Amino acid metabolism
	VDAG_JR2_Chr4g11880a-00001	SsuA/THI5-like	Thiamine metabolism
Found in 3/4	VDAG_JR2_Chr4g11470a-00001	Glutathione S-transferase	Redox metabolism, protein binding
	VDAG_JR2_Chr4g00470a-00001	Glycosyl hydrolase, family 13	Carbohydrate metabolism
	VDAG_JR2_Chr3g07650a-00001	Aliphatic acid kinase, short-chain	organic acid metabolism, phosphorylation
	VDAG_JR2_Chr1g02490a-00001	Thiamine pyrophosphate enzyme	Thiamine metabolism
	VDAG_JR2_Chr1g28705a-00001	RNA recognition motif domain	Nucleic acid binding
	VDAG_JR2_Chr8g07440a-00001	Aldehyde dehydrogenase domain	Redox metabolism
	VDAG_JR2_Chr4g09700a-00001	Homogentisate 1,2-dioxygenase	Amino acid metabolism
	VDAG_JR2_Chr1g18410a-00001	Ubiquitin-activating enzyme E1	Posttranslational protein modification
