# Supplementary material for: The velvet protein Vel1 controls initial plant root colonization and conidia formation for xylem distribution in Verticillium wilt
Source: PLoS Genet. 2021 Mar 15;17(3):e1009434. doi: 10.1371/journal.pgen.1009434 (PMC7993770; doi:10.1371/journal.pgen.1009434)
Supplement: S3 Table — (PDF) [file pgen.1009434.s027.pdf]

**S3 Table. Proteins significantly enriched with Vel3-GFP and their predicted domains and functions.** During data analysis the command “Replace missing values from normal distribution” was repeated four times. Proteins enriched in all four repetitions are displayed as “Found in 4/4”, proteins found in three repetitions are displayed as “Found in 3/4”.

| | Protein ID | Predicted domain | Potential function |
|---|---|---|---|
| Found in 4/4 | VDAG_JR2_Chr6g00630a-00001(Vel3) | Velvet domain | Development, protein binding |
| | VDAG_JR2_Chr3g12090a-00001(Vos1) | Velvet domain | Development, protein binding |
