# Supplementary material for: The velvet protein Vel1 controls initial plant root colonization and conidia formation for xylem distribution in Verticillium wilt
Source: PLoS Genet. 2021 Mar 15;17(3):e1009434. doi: 10.1371/journal.pgen.1009434 (PMC7993770; doi:10.1371/journal.pgen.1009434)
Supplement: S4 Table — (PDF) [file pgen.1009434.s028.pdf]

S4 Table. Proteins significantly enriched with Vos1-GFP and their predicted domains and functions. During data analysis the command "Replace missing values from normal distribution" was repeated four times. Proteins enriched in all four repetitions are displayed as "Found in 4/4", proteins found in three repetitions are displayed as "Found in 3/4".

	Protein ID	Predicted domain	Potential function
Found in 4/4	VDAG_JR2_Chr3g12090a-00001(Vos1)	Velvet domain	Development, protein binding
	VDAG_JR2_Chr3g00030a-00001	ATP synthase	Energy metabolism
	VDAG_JR2_Chr1g18720a-00001	Isopropylmalate dehydrogenase-like domain	Carbohydrate metabolism, redox metabolism
	VDAG_JR2_Chr6g07320a-00001	Aminotransferase class-III	Amino acid metabolism
	VDAG_JR2_Chr1g16720a-00001	Eukaryotic porin/Tom40	Transport
	VDAG_JR2_Chr4g00570a-00001	Mitochondrial substrate/solute carrier	Transport
	VDAG_JR2_Chr5g09480a-00001	Ribosomal protein L32e	Protein synthesis
	VDAG_JR2_Chr8g06840a-00001	FKBP-type peptidyl-prolyl cis-trans isomerase domain	Protein folding, protein modification
	VDAG_JR2_Chr6g06940a-00001	Peptidase M16	Proteolysis
	VDAG_JR2_Chr6g00630a-00001(Vel3)	Velvet domain	Development, protein binding
	VDAG_JR2_Chr6g01510a-00001	Small GTPase superfamily, ARF/SAR type	Intracellular trafficking
	VDAG_JR2_Chr4g02360a-00001	Mitochondrial substrate/solute carrier	Transport
	VDAG_JR2_Chr3g05360a-00001	Calreticulin/calnexin	Protein folding
	VDAG_JR2_Chr3g06150a-00001(Vel2)	Velvet domain	Development, Protein binding
	VDAG_JR2_Chr3g03520a-00001	Chaperone DnaJ	Protein folding, stress reponse
	VDAG_JR2_Chr7g04550a-00001	ATPase, OSCP/delta subunit	Energy metabolism
	VDAG_JR2_Chr3g01800a-00001	Cytochrome c1	Energy metabolism
	VDAG_JR2_Chr1g07570a-00001	Cytochrome c oxidase, subunit Va/VI	Energy metabolism
	VDAG_JR2_Chr7g01800a-00001	Pyruvate carboxyltransferase	Carbohydrate metabolism
	VDAG_JR2_Chr3g02140a-00001	Leucine-rich repeat	Protein binding
	VDAG_JR2_Chr3g11170a-00001		
	VDAG_JR2_Chr1g16160a-00001	Cytochrome b-c1 complex subunit 8	Energy metabolism
	VDAG_JR2_Chr8g08950a-00001	Aldehyde dehydrogenase domain	Redox metabolism
	VDAG_JR2_Chr8g08440a-00001	Pyruvate carboxyltransferase	Carbohydrate metabolism
Found in 3/4	VDAG_JR2_Chr1g24260a-00001	Cytochrome b-c1 complex subunit 10	Energy metabolism
	VDAG_JR2_Chr1g12360a-00001	NADP-dependent oxidoreductase domain	Redox metabolism
	VDAG_JR2_Chr8g04870a-00001	Band 7 domain	Membrane protein
	VDAG_JR2_Chr3g11230a-00001	Sulfite reductase flavoprotein alpha-component-like, FAD-binding	Redox metabolism
	VDAG_JR2_Chr1g14040a-00001	Anion-transporting ATPase-like domain	Transport
	VDAG_JR2_Chr1g24330a-00001	Importin-beta, N-terminal domain	Transport
