# Supplementary material for: The velvet protein Vel1 controls initial plant root colonization and conidia formation for xylem distribution in Verticillium wilt
Source: PLoS Genet. 2021 Mar 15;17(3):e1009434. doi: 10.1371/journal.pgen.1009434 (PMC7993770; doi:10.1371/journal.pgen.1009434)
Supplement: S5 Table — (PDF) [file pgen.1009434.s029.pdf]

S5 Table. Significantly enriched proteins with LFQ intensities, MS/MS count, sequence coverage and unique peptides in all three replicates of Vel1-GFP in comparison to the wild type.

	LFQ intensity						MS/MS count						Sequence coverage [%]						Unique peptides						
	wt			Vel1			wt			Vel1			wt			Vel1			wt			Vel1			
	1	2	3	1	2	3	1	2	3	1	2	3	1	2	3	1	2	3	1	2	3	1	2	3	Protein ID
Found in 4/4	28,55	28,49	28,55	29,12	29,11	29,17	16	10	12	20	16	11	47,1	26,7	32,9	32,9	32,9	29,2	8	5	6	7	7	5	VDAG_JR2_Chr7g00220a-00001
	NaN	NaN	NaN	27,63	28,62	29,02	0	0	0	27	27	24	0	0	0	47,6	52,2	38,4	0	0	0	20	21	19	VDAG_JR2_Chr7g04890a-00001(Vel1)
	27,03	27,04	26,89	27,81	27,84	27,88	19	9	11	25	24	19	36,6	19,2	22,8	43,8	45	39,6	16	8	10	18	20	17	VDAG_JR2_Chr7g05280a-00001
	NaN	NaN	NaN	26,27	27,33	27,36	0	0	0	12	17	8	0	0	0	20,2	35,7	17,6	0	0	0	10	13	8	VDAG_JR2_Chr3g06150a-00001(Vel2)
	23,83	23,39	24,06	25,19	25,87	25,92	4	2	5	15	14	13	10,5	6,3	14,6	27,5	25	25,6	4	2	5	13	13	12	VDAG_JR2_Chr6g10140a-00001
	24,84	24,24	24,57	25,86	26	25,69	7	3	5	16	15	12	13,5	5	9	26,6	21,2	19	7	3	5	15	14	12	VDAG_JR2_Chr5g09190a-00001
	NaN	NaN	NaN	24,09	24,24	24,01	1	1	1	7	3	2	6,9	6,9	6	37,1	21,1	12,9	1	1	1	6	3	2	VDAG_JR2_Chr3g09450a-00001
Found in 3/4	25,75	25,55	25,93	26,66	26,79	26,53	11	5	7	12	14	10	29,3	16	20,4	40,4	40,4	32,2	9	5	6	11	13	9	VDAG_JR2_Chr8g02960a-00001
	NaN	NaN	NaN	24,42	24,65	24,77	1	2	2	9	7	6	1,4	4,5	4,9	17,2	14,7	11,4	1	2	2	8	7	6	VDAG_JR2_Chr5g05440a-00001
	NaN	22,4	NaN	23,92	23,91	23,96	2	2	0	4	3	2	5,6	6,3	0	11,8	9,4	6,3	2	2	0	4	3	2	VDAG_JR2_Chr1g28705a-00001
	23	NaN	NaN	23,97	23,67	23,8	2	1	0	8	3	4	3,5	1,9	0	13	3,3	6,5	2	1	0	7	2	4	VDAG_JR2_Chr3g02760a-00001

23 30

Log2(x)LFQ intensity
