# Supplementary material for: The velvet protein Vel1 controls initial plant root colonization and conidia formation for xylem distribution in Verticillium wilt
Source: PLoS Genet. 2021 Mar 15;17(3):e1009434. doi: 10.1371/journal.pgen.1009434 (PMC7993770; doi:10.1371/journal.pgen.1009434)
Supplement: S6 Table — (PDF) [file pgen.1009434.s030.pdf]

S6 Table. Significantly enriched proteins with LFQ intensities, MS/MS count, sequence coverage and unique peptides in all three replicates of Vel2-GFP in comparison to the wild type.

	LFQ intensity						MS/MS count						Sequence coverage [%]						Unique peptides						
	wt			Vel2			wt			Vel2			wt			Vel2			wt			Vel2			
	1	2	3	1	2	3	1	2	3	1	2	3	1	2	3	1	2	3	1	2	3	1	2	3	Protein ID
Found in 4/4	NaN	NaN	NaN	30,33	31,10	30,95	0	0	0	34	39	33	0	0	0	40,4	41,5	36,1	0	0	0	18	19	16	VDAG_JR2_Chr3g06150a-00001(Vel2)
	28,00	27,27	27,81	29,72	30,35	29,13	15	8	9	23	23	19	59,3	40,2	40,7	64,7	64,7	59,3	11	7	8	14	13	12	VDAG_JR2_Chr5g03080a-00001
	27,38	27,52	28,50	29,34	29,62	29,68	25	18	22	41	41	38	44,3	31,5	36,6	50,5	55,9	54,8	22	15	18	28	27	26	VDAG_JR2_Chr4g06160a-00001
	29,05	28,91	28,89	29,52	29,46	29,56	29	20	20	33	27	27	72,2	54,5	55,1	75,9	65,6	63,1	19	14	14	20	18	16	VDAG_JR2_Chr4g02640a-00001
	27,64	27,39	27,96	29,52	29,42	29,40	18	8	11	23	23	16	47	31,6	32,5	47,5	49,6	45,2	14	8	8	14	15	12	VDAG_JR2_Chr4g06150a-00001
	28,55	28,49	28,55	29,42	29,38	29,15	16	10	12	21	19	17	47,1	26,7	32,9	32,9	32,9	32,9	8	5	6	7	7	7	VDAG_JR2_Chr7g00220a-00001
	28,46	28,62	28,41	29,02	29,17	28,93	23	18	21	31	27	29	59,8	42,8	43	64	55,6	54,9	19	15	17	25	23	24	VDAG_JR2_Chr8g05200a-00001
	27,72	28,29	28,42	29,30	29,17	29,39	11	9	9	23	23	20	43,1	33,9	39,1	63,2	52,3	53,6	8	7	7	12	10	11	VDAG_JR2_Chr5g10680a-00001
	27,54	28,13	28,10	28,73	29,02	29,05	17	10	15	22	29	28	24,7	12,3	16,1	19,1	24,1	25,9	16	9	13	15	20	21	VDAG_JR2_Chr2g02650a-00001
	27,12	25,89	27,77	28,96	29,01	28,57	6	3	4	12	10	7	45,2	34,2	34,2	51,4	45,2	45,2	5	3	3	6	5	5	VDAG_JR2_Chr5g01680a-00001
	28,05	28,35	28,43	29,01	28,98	29,33	49	36	43	72	68	81	34,3	25,2	29,6	40	36,1	39,3	48	37	39	60	56	64	VDAG_JR2_Chr1g20590a-00001
	27,77	28,11	27,82	28,74	28,90	28,64	45	27	24	61	60	56	23,9	16,1	14,9	30,7	28,1	28,9	42	27	25	54	51	49	VDAG_JR2_Chr6g02720a-00001
	27,73	27,84	27,73	28,49	28,66	28,35	15	11	9	16	17	14	64,9	45,7	33,2	60	52,5	56,6	11	9	7	14	11	11	VDAG_JR2_Chr6g01710a-00001
	27,20	27,33	27,57	28,22	28,46	28,29	31	23	26	43	38	45	24,9	16,3	15,2	24,8	22,5	26,3	31	22	22	37	32	38	VDAG_JR2_Chr1g20610a-00001
	27,12	27,24	27,27	27,87	28,41	27,89	16	9	12	18	18	15	41,7	22,5	27,5	35,2	34,9	34,5	13	7	10	13	13	12	VDAG_JR2_Chr2g07360a-00001
	NaN	NaN	NaN	27,85	28,37	28,75	0	0	0	20	26	25	0	0	0	29,2	46	42,6	0	0	0	15	19	18	VDAG_JR2_Chr7g04890a-00001(Vel1)
	26,82	26,37	26,43	28,33	28,22	27,34	8	4	7	12	9	8	72,1	47,1	72,1	78,7	71,3	72,1	6	4	6	7	6	6	VDAG_JR2_Chr1g13580a-00001
	27,21	26,59	26,78	27,98	27,99	27,52	17	8	13	21	20	18	66,3	46,7	60,9	71	73,6	69,2	12	8	11	14	15	13	VDAG_JR2_Chr4g10440a-00001
	27,03	27,04	26,89	28,14	27,88	28,16	19	9	11	23	21	23	36,6	19,2	22,8	44	40,4	45	16	8	10	19	17	19	VDAG_JR2_Chr7g05280a-00001
	26,73	26,56	27,25	28,07	27,85	27,95	14	7	10	21	16	21	40,1	25,6	31,9	54,2	45,5	52,1	12	7	8	16	14	17	VDAG_JR2_Chr8g10760a-00001
	26,82	26,85	26,92	27,79	27,60	27,52	11	7	7	17	12	14	29,7	19	24,2	37,4	29,5	33,1	10	6	7	15	11	13	VDAG_JR2_Chr8g08780a-00001
	25,75	25,55	25,93	27,10	27,51	26,80	11	5	7	16	15	10	29,3	16	20,4	47,2	45,9	32,2	9	5	6	14	14	9	VDAG_JR2_Chr8g02960a-00001
	26,67	26,89	26,85	27,37	27,47	27,45	22	12	14	21	15	12	44,8	24,5	27,5	40,7	27,3	25	20	11	13	17	13	10	VDAG_JR2_Chr8g04960a-00001
	25,60	25,45	25,82	26,61	27,16	26,81	11	8	7	18	15	19	36,2	30,7	25,7	45,4	41,6	48,6	9	8	7	15	13	17	VDAG_JR2_Chr1g18400a-00001
	25,18	24,99	26,34	27,88	27,12	27,31	5	4	8	20	12	15	25,7	16,9	36,9	59,5	48,6	54,7	5	4	7	15	11	13	VDAG_JR2_Chr4g07720a-00001
	25,86	25,62	25,28	27,01	26,82	26,89	6	3	3	11	10	10	27,1	18,8	17,5	41,2	36,7	37,1	5	3	3	10	8	9	VDAG_JR2_Chr1g11550a-00001
	24,07	NaN	NaN	26,04	26,71	26,29	2	1	1	5	5	7	9,2	4,9	4,3	21,2	23,9	33,7	2	1	1	4	4	6	VDAG_JR2_Chr6g06890a-00001
	25,29	24,52	25,09	26,39	26,63	26,04	3	2	3	6	5	3	58,4	37,3	58,4	58,4	58,4	58,4	3	2	3	3	3	3	VDAG_JR2_Chr7g08750a-00001
	NaN	NaN	NaN	25,39	26,15	25,86	0	1	1	6	10	9	0	2,5	2,5	19,7	16,9	19,5	0	1	1	7	8	7	VDAG_JR2_Chr4g08190a-00001
	23,83	23,39	24,06	26,47	26,08	26,29	4	2	5	17	10	14	10,5	6,3	14,6	30,8	20,2	27,3	4	2	5	15	10	14	VDAG_JR2_Chr6g10140a-00001
	24,84	24,24	24,57	25,72	26,06	25,96	7	3	5	9	11	13	13,5	5	9	15,9	15,3	21,1	7	3	5	9	11	14	VDAG_JR2_Chr5g09190a-00001
	NaN	NaN	NaN	24,70	26,05	25,45	3	1	1	2	2	2	30,2	10,9	10,9	10,9	10,9	10,9	2	1	1	1	1	1	VDAG_JR2_Chr1g17980a-00001
	24,45	23,16	23,34	26,24	25,70	26,11	5	2	2	12	8	16	33	13,6	11,4	50,4	44,7	69,7	4	2	2	9	7	14	VDAG_JR2_Chr4g02180a-00001
	24,28	24,41	24,63	25,51	25,61	25,28	4	4	2	6	4	5	11,2	11	4,9	13,8	8,8	13,8	4	4	2	5	3	5	VDAG_JR2_Chr2g08140a-00001
	24,20	24,71	24,76	25,61	25,44	25,34	3	4	6	12	7	6	6,4	8,3	9,9	20,4	13,1	11,9	3	4	5	11	7	6	VDAG_JR2_Chr3g03700a-00001
	NaN	NaN	NaN	25,06	25,33	25,14	1	2	2	11	11	9	1,4	4,5	4,9	22,7	20,5	15,7	1	2	2	11	10	9	VDAG_JR2_Chr5g05440a-00001
	24,40	23,65	23,71	25,48	25,28	25,23	8	2	2	12	7	9	18,8	4,7	3,7	29,4	21,3	23,9	8	2	2	12	7	9	VDAG_JR2_Chr7g00600a-00001
	23,55	23,55	24,39	25,31	25,16	24,77	2	2	4	5	7	6	9	9,3	20,2	22,1	24,4	29,5	2	2	4	6	6	6	VDAG_JR2_Chr4g07360a-00001
	NaN	NaN	23,26	24,32	24,89	24,58	0	0	3	9	10	8	0	0	3,1	10,4	9,5	7,8	0	0	3	9	10	8	VDAG_JR2_Chr3g10860a-00001
	23,82	NaN	23,27	24,92	24,69	25,07	2	2	2	3	3	3	17	7,2	7,2	17	17	17	2	1	1	2	2	2	VDAG_JR2_Chr3g01680a-00001
	NaN	NaN	NaN	24,61	24,50	24,80	0	0	0	4	3	5	0	0	0	18,2	14,8	24	0	0	0	4	3	6	VDAG_JR2_Chr3g12090a-00001(Vos1)
	NaN	NaN	NaN	24,43	24,08	24,07	1	0	0	4	5	3	4,5	0	4,5	21,1	16,3	19,2	1	0	1	4	4	4	VDAG_JR2_Chr4g08010a-00001
	NaN	NaN	NaN	25,34	24,08	26,68	0	0	2	10	6	12	0	0	10,1	30,4	19,4	31,3	0	0	2	7	5	6	VDAG_JR2_Chr4g11880a-00001
Found in 3/4	24,99	NaN	25,26	27,34	27,17	26,55	6	2	4	14	13	11	25,6	14	26,4	52,1	55,4	51,2	5	2	4	11	12	10	VDAG_JR2_Chr4g11470a-00001
	NaN	NaN	NaN	24,60	24,85	24,04	0	0	0	4	6	3	0	0	0	13,1	16,3	9,8	0	0	0	4	5	3	VDAG_JR2_Chr4g00470a-00001
	23,68	NaN	NaN	24,09	24,74	24,76	4	0	1	5	7	6	14,9	0	3,1	18,6	26	17,1	4	0	1	5	7	5	VDAG_JR2_Chr3g07650a-00001
	NaN	NaN	NaN	23,79	24,05	23,63	1	0	1	6	5	4	2,3	0	1,9	21	13,2	11,3	1	0	1	6	5	4	VDAG_JR2_Chr1g02490a-00001
	NaN	22,4	NaN	23,42	24,02	23,85	2	2	0	3	4	3	5,6	6,3	0	8,9	11,8	8,9	2	2	0	3	4	3	VDAG_JR2_Chr1g28705a-00001
	NaN	NaN	NaN	23,91	24,01	24,13	0	0	0	5	5	6	0	0	2,4	14,6	14,2	18,8	0	0	1	5	5	7	VDAG_JR2_Chr8g07440a-00001
	22,69	NaN	NaN	24,02	23,85	24,1	1	1	0	5	4	5	11,9	5,8	0	16	14,3	16	3	1	0	5	4	5	VDAG_JR2_Chr4g09700a-00001
	23,13	22,31	NaN	24,63	23,74	23,91	3	1	2	9	5	7	6,8	4,3	2,3	10,7	6,5	8,2	4	2	2	7	3	7	VDAG_JR2_Chr1g18410a-00001

23 32

Log2(x)LFQ intensity
