# Supplementary material for: The velvet protein Vel1 controls initial plant root colonization and conidia formation for xylem distribution in Verticillium wilt
Source: PLoS Genet. 2021 Mar 15;17(3):e1009434. doi: 10.1371/journal.pgen.1009434 (PMC7993770; doi:10.1371/journal.pgen.1009434)
Supplement: S7 Table — (PDF) [file pgen.1009434.s031.pdf]

S7 Table. Significantly enriched proteins with LFQ intensities, MS/MS count, sequence coverage and unique peptides in all three replicates of Vel3-GFP in comparison to the wild type.

	LFQ intensity						MS/MS count						Sequence coverage [%]						Unique peptides						
	wt			Vel3			wt			Vel3			wt			Vel3			wt			Vel3			
	1	2	3	1	2	3	1	2	3	1	2	3	1	2	3	1	2	3	1	2	3	1	2	3	Protein ID
Found in 4/4	NaN	NaN	NaN	30,86	31,04	31,37	0	0	0	46	40	34	0	0	0	54,7	52,2	49,4	0	0	0	20	21	17	VDAG_JR2_Chr6g00630a-00001(Vel3)
	NaN	NaN	NaN	27,87	25,94	27,75	0	0	0	8	4	10	0	0	0	26,4	13,5	24	0	0	0	8	4	7	VDAG_JR2_Chr3g12090a-00001(Vos1)

25 32

Log2(x)LFQ intensity
