# Supplementary material for: The velvet protein Vel1 controls initial plant root colonization and conidia formation for xylem distribution in Verticillium wilt
Source: PLoS Genet. 2021 Mar 15;17(3):e1009434. doi: 10.1371/journal.pgen.1009434 (PMC7993770; doi:10.1371/journal.pgen.1009434)
Supplement: S8 Table — (PDF) [file pgen.1009434.s032.pdf]

S8 Table. Significantly enriched proteins with LFQ intensities, MS/MS count, sequence coverage and unique peptides in all three replicates of Vos1-GFP in comparison to the wild type.

	LFQ intensity						MS/MS count						Sequence coverage [%]						Unique peptides						
	wt			Vos1			wt			Vos1			wt			Vos1			wt			Vos1			
	1	2	3	1	2	3	1	2	3	1	2	3	1	2	3	1	2	3	1	2	3	1	2	3	Protein ID
Found in 4/4	NaN	NaN	NaN	27,42	27,53	26,83	0	0	0	29	27	24	0	0	0	67	66	60,7	0	0	0	15	15	14	VDAG_JR2_Chr3g12090a-00001(Vos1)
	25,55	25,60	25,23	26,20	26,11	26,05	12	15	14	23	20	18	24,9	31,8	28,5	33,9	41,6	33,2	10	11	10	15	16	14	VDAG_JR2_Chr3g00030a-00001
	25,32	25,30	25,12	25,64	25,72	25,68	10	19	18	21	21	21	34,3	47,6	47,6	44,8	51,3	50,3	10	14	15	15	16	17	VDAG_JR2_Chr1g18720a-00001
	24,02	24,13	22,58	25,76	25,55	25,40	11	10	5	29	21	21	28,3	28,9	22,8	65,4	52,2	46,5	10	8	5	21	18	16	VDAG_JR2_Chr6g07320a-00001
	24,71	24,07	23,95	25,34	25,20	25,08	10	9	8	18	15	15	38,2	33,9	33,9	45,6	42,4	45,6	8	7	7	12	12	12	VDAG_JR2_Chr1g16720a-00001
	24,38	24,17	23,82	25,02	25,10	24,86	6	6	5	12	10	11	25,9	25,6	23,5	49,1	32,7	38	5	4	4	8	6	7	VDAG_JR2_Chr4g00570a-00001
	23,40	22,97	23,37	24,57	24,75	23,87	3	5	3	4	6	2	23,7	36,6	23,7	36,6	36,6	23,7	2	3	2	3	3	2	VDAG_JR2_Chr5g09480a-00001
	23,38	23,60	23,38	24,54	24,29	24,29	4	8	6	11	9	6	12,6	16,5	16,5	16,5	16,5	13,7	4	7	6	7	7	5	VDAG_JR2_Chr8g06840a-00001
	21,64	22,70	22,53	24,14	24,21	23,26	2	10	5	10	11	8	8,3	35,6	19,7	31,2	27,7	36	2	8	5	8	8	8	VDAG_JR2_Chr6g06940a-00001
	NaN	NaN	NaN	24,39	24,10	24,11	0	0	0	12	9	8	0	0	0	31,4	26,5	24,7	0	0	0	8	7	7	VDAG_JR2_Chr6g00630a-00001(Vel3)
	23,52	23,47	23,69	24,04	23,97	24,00	5	7	3	6	5	4	37	28,6	22,8	35,4	37	28,6	5	5	3	5	5	4	VDAG_JR2_Chr6g01510a-00001
	23,01	22,56	22,57	24,65	23,94	23,73	3	5	4	16	10	8	7,4	11	14,5	41	25,2	18,4	2	4	4	12	8	7	VDAG_JR2_Chr4g02360a-00001
	23,34	23,30	23,36	23,63	23,71	23,72	4	4	4	11	8	8	11,5	11,8	12,2	26,3	21,7	21,7	4	4	5	9	8	8	VDAG_JR2_Chr3g05360a-00001
	NaN	NaN	NaN	23,81	23,58	23,19	0	0	0	9	9	8	0	0	0	20	17,8	16,7	0	0	0	8	7	7	VDAG_JR2_Chr3g06150a-00001(Vel2)
	22,03	22,35	22,00	22,65	22,80	22,73	2	4	4	3	4	4	7,9	16,5	21,8	16,5	16,5	16,5	2	4	5	4	4	4	VDAG_JR2_Chr3g03520a-00001
	21,55	21,98	21,69	22,94	22,72	22,73	4	5	2	5	4	6	24	24	13,3	28,9	24	28,9	4	4	2	4	4	5	VDAG_JR2_Chr7g04550a-00001
	NaN	NaN	NaN	22,23	22,71	22,20	1	2	1	5	2	4	4,1	4,1	4,1	16,7	9,1	9,1	1	1	1	4	2	3	VDAG_JR2_Chr3g01800a-00001
	21,79	21,42	21,59	22,67	22,45	22,30	2	2	2	4	3	3	18,8	23,8	23,8	32,5	32,5	32,5	2	2	2	4	3	3	VDAG_JR2_Chr1g07570a-00001
	NaN	NaN	NaN	21,63	21,92	21,95	1	1	1	3	4	3	7,6	6,8	3,7	17,4	21,7	11,6	1	1	1	3	4	3	VDAG_JR2_Chr7g01800a-00001
	NaN	NaN	NaN	21,69	21,88	21,66	0	3	1	2	4	2	0	10,2	3,6	14,8	19	8,8	0	2	1	4	5	3	VDAG_JR2_Chr3g02140a-00001
	21,14	NaN	NaN	22,68	21,86	21,74	2	1	1	10	3	5	2,5	1,4	1,9	11,8	5,2	8,2	2	1	1	9	3	5	VDAG_JR2_Chr3g11170a-00001
	NaN	NaN	NaN	21,88	21,82	21,63	1	0	0	3	2	3	8,4	0	0	28,2	16,8	29,8	1	0	0	3	2	3	VDAG_JR2_Chr1g16160a-00001
	NaN	NaN	NaN	21,56	21,37	21,89	1	1	1	3	2	2	2,4	2,8	2,4	9	5,2	5,2	1	1	1	3	2	2	VDAG_JR2_Chr8g08950a-00001
	21,07	20,70	20,93	21,56	21,33	21,53	3	3	3	4	2	3	6	8	8	12,5	4,4	9,1	3	3	3	4	2	3	VDAG_JR2_Chr8g08440a-00001
Found in 3/4	NaN	21,13	NaN	22,00	21,99	21,95	1	3	1	3	5	3	15,4	61,5	15,4	34,1	62,6	62,6	1	3	1	2	4	3	VDAG_JR2_Chr1g24260a-00001
	NaN	NaN	NaN	22,07	21,88	21,92	2	2	1	5	2	3	13,3	4,6	3,2	15	7,8	10,7	3	1	1	5	2	3	VDAG_JR2_Chr1g12360a-00001
	NaN	NaN	NaN	21,67	21,71	21,55	0	1	1	3	3	2	0	8,4	6,2	19,5	19,5	11	0	1	1	3	3	2	VDAG_JR2_Chr8g04870a-00001
	NaN	20,57	NaN	21,42	21,58	21,51	0	2	0	5	5	3	0	2,9	0	6,7	7,8	5,1	0	2	0	5	5	4	VDAG_JR2_Chr3g11230a-00001
	NaN	20,87	NaN	21,66	21,49	21,62	1	3	1	2	3	3	8,6	18,3	2,7	8,6	12,4	11,2	2	4	1	2	3	3	VDAG_JR2_Chr1g14040a-00001
	NaN	NaN	NaN	21,43	21,46	21,37	1	2	1	3	4	4	1,4	2,5	1,1	4,2	5,7	5,8	1	2	1	3	4	4	VDAG_JR2_Chr1g24330a-00001

21 28
Log2(x)LFQ intensity
