# Supplementary material for: The velvet protein Vel1 controls initial plant root colonization and conidia formation for xylem distribution in Verticillium wilt
Source: PLoS Genet. 2021 Mar 15;17(3):e1009434. doi: 10.1371/journal.pgen.1009434 (PMC7993770; doi:10.1371/journal.pgen.1009434)
Supplement: S9 Table — (PDF) [file pgen.1009434.s033.pdf]

S9 Table. *A. tumefaciens*, *E. coli* and *V. dahliae* strains used in this study.

Strain name	Description	Reference
Agrobacterium tumefaciens		
AGL1	Used for A*. tumefaciens*-mediated transformation of *V. dahliae*	[1]
Escherichia coli		
DH5α	Used for cloning and extraction of plasmid DNA	Invitrogen
Verticillium dahliae		
JR2<br>WT	Wild type isolate from *Solanum lycopersicum*	[2]
VGB18<br>(Δ*LAE1*)	Δ*LAE1::NAT*^{R}	[3]
VGB22<br>(WT/*HISTONE-RFP*)	^{P}*GPDA:H2B:RFP:TRPC*^{T}*:*^{P}*GPDA:NAT*^{R}	This study
VGB45<br>(WT/*OE-GFP*)	^{P}*GPDA:GFP:TRPC*^{T}*:*^{P}*GPDA:HYG*^{R}*:TRPC*^{T}	[4]
VGB58/VGB59<br>(Δ*VEL2*)	Δ*VEL2::*^{P}*GPDA:NAT*^{R}	This study
VGB64/VGB65<br>(Δ*VOS1*)	Δ*VOS1::*^{P}*GPDA:NAT*^{R}	This study
VGB219/VGB220<br>(*OE-VOS1-GFP*/*HISTONE-RFP*)	^{P}*GPDA:H2B:RFP:TRPC*^{T}*:*^{P}*GPDA:NAT*^{R}*;*<br>^{P}*GPDA:VOS1:GFP:TRPC*^{T}*:*^{P}*GPDA:HYG*^{R}*:TRPC*^{T}	This study
VGB223<br>(*OE-VEL2-GFP*/*HISTONE-RFP*)	^{P}*GPDA:H2B:RFP:TRPC*^{T}*:*^{P}*GPDA:NAT*^{R}*;*<br>^{P}*GPDA:VEL2:GFP:TRPC*^{T}*:*^{P}*GPDA:HYG*^{R}*:TRPC*^{T}	This study
VGB234/VGB235<br>(Δ*VEL3*)	Δ*VEL3::*^{P}*GPDA:NAT*^{R}*:TRPC*^{T}	This study
VGB241/VGB242<br>(Δ*VOS1*)	Δ*VOS1::*^{P}*GPDA:HYG*^{R}*:TRPC*^{T}	This study
VGB246/VGB247<br>(Δ*VEL1*)	Δ*VEL1::*^{P}*GPDA:HYG*^{R}*:TRPC*^{T}	This study
VGB281/VGB282<br>(Δ*VEL1*/Δ*VEL2*)	Δ*VEL2::*^{P}*GPDA:NAT*^{R}*;* Δ*VEL1::*^{P}*GPDA:HYG*^{R}*:TRPC*^{T}	This study
VGB289/VGB290<br>(Δ*VEL3*/Δ*VEL1*)	Δ*VEL1::*^{P}*GPDA:HYG*^{R}*:TRPC*^{T}*;*<br>Δ*VEL3::*^{P}*GPDA:NAT*^{R}*:TRPC*^{T}	This study
VGB297<br>(*OE-VEL1*)	^{P}*GPDA:VEL1:GFP:TRPC*^{T}*:*^{P}*GPDA:HYG*^{R}*:TRPC*^{T}	This study
VGB362<br>(Δ*VEL2/OE-VEL1-GFP)*	Δ*VEL2::*^{P}*GPDA:NAT*^{R}*;*<br>^{P}*GPDA:VEL1:GFP:TRPC*^{T}*:*^{P}*GPDA:HYG*^{R}*:TRPC*^{T}	This study
VGB364/VGB365<br>(*OE-VEL1-GFP*/*HISTONE-RFP*)	^{P}*GPDA:H2B:RFP:TRPC*^{T}*:*^{P}*GPDA:NAT*^{R}*;*<br>^{P}*GPDA:VEL1:GFP:TRPC*^{T}*:*^{P}*GPDA:HYG*^{R}*:TRPC*^{T}	This study
VGB373<br>(Δ*VOS1*/Δ*VEL3*)	Δ*VEL3::*^{P}*GPDA:NAT*^{R}*:TRPC*^{T}*;*<br>Δ*VOS1::*^{P}*GPDA:HYG*^{R}*:TRPC*^{T}*;*	This study

S9 Table. *A. tumefaciens*, *E. coli* and *V. dahliae* strains used in this study, continued.

Strain name	Description	Reference
VGB375 (Comp. *VEL2*)	ΔVEL2::^{P}VEL2:VEL2:GFP:^{P}GPDA:HYG^{R}:TRPC^{T}:VEL2^{T}	This study
VGB392 (WT/*OE-GFP*)	^{P}GPDA:GFP:TRPC^{T}:^{P}GPDA:NAT^{R}:TRPC^{T}	[5]
VGB443/VGB444 (Δ*VEL1*/*OE-GFP*)	ΔVEL1::^{P}GPDA:HYG^{R}:TRPC^{T}; ^{P}GPDA:GFP:TRPC^{T}:^{P}GPDA:NAT^{R}:TRPC^{T}	This study
VGB445/VGB446 (Comp. *VEL3*)	ΔVEL3::^{P}VEL3:VEL3:^{P}GPDA:HYG^{R}:TRPC^{T}:VEL3^{T}	This study
VGB447 (*VEL1-GFP*)	^{P}VEL1:VEL1:GFP:^{P}GPDA:HYG^{R}:TRPC^{T}:VEL1^{T}	This study
VGB450 (*VEL2-GFP*)	^{P}VEL2:VEL2:GFP:^{P}GPDA:HYG^{R}:TRPC^{T}:VEL2^{T}	This study
VGB451 (*VEL3-GFP*)	^{P}VEL3:VEL3:GFP:^{P}GPDA:HYG^{R}:TRPC^{T}:VEL3^{T}	This study
VGB453 (*VOS1-GFP*)	^{P}VOS1:VOS1:GFP:^{P}GPDA:HYG^{R}:TRPC^{T}:VOS1^{T}	This study
VGB474 (Comp. *VEL1*)	ΔVEL1::^{P}VEL1:VEL1^{P}GPDA:NAT^{R}:TRPC^{T}:VEL1^{T}	This study
VGB477 (WT/*HISTONE-RFP*)	^{P}GPDA:H2B:RFP:TRPC^{T}:^{P}GPDA:GEN^{R}:TRPC^{T}	[5]
VGB495 (Δ*VEL1*/*HISTONE-RFP*)	^{P}GPDA:H2B:RFP:TRPC^{T}:^{P}GPDA:GEN^{R}:TRPC^{T}; ΔVEL1::^{P}GPDA:NAT^{R}:TRPC^{T}	This study
VGB496/VGB497 (Δ*VEL1*/*OE-VEL2-GFP*/*HISTONE-RFP*)	^{P}GPDA:H2B:RFP:TRPC^{T}:^{P}GPDA:GEN^{R}:TRPC^{T}; ΔVEL1::^{P}GPDA:NAT^{R}:TRPC^{T} ^{P}GPDA:VEL2:GFP:TRPC^{T}:^{P}GPDA:HYG^{R}:TRPC^{T}	This study
VGB498/VGB499 (Δ*VEL2*/*OE-VEL1-GFP*/*HISTONE-RFP*)	ΔVEL2::^{P}GPDA:NAT^{R}; ^{P}GPDA:VEL1:GFP:TRPC^{T}:^{P}GPDA:HYG^{R}:TRPC^{T}; ^{P}GPDA:H2B:RFP:TRPC^{T}:^{P}GPDA:GEN^{R}:TRPC^{T}	This study
VGB501/VGB502 (*OE-VEL3-GFP*/*HISTONE-RFP*)	^{P}GPDA:H2B:RFP:TRPC^{T}:^{P}GPDA:NAT^{R}; ^{P}GPDA:VEL3:GFP:TRPC^{T}:^{P}GPDA:HYG^{R}:TRPC^{T}	This study

^{P}=promoter, ^{T}=terminator, NAT^{R}=nourseothricin resistance marker, HYG^{R}=hygromycin B resistance marker, GEN^{R}= geneticin resistance marker

References

1. Lazo GR, Stein PA, Ludwig RA. A DNA transformation-competent Arabidopsis genomic library in Agrobacterium. Biotechnology (N Y). 1991;9:963–967.

2. Fradin EF, Zhang Z, Juarez Ayala JC, Castroverde CDM, Nazar RN, Robb J, et al. Genetic dissection of Verticillium wilt resistance mediated by tomato Ve1. Plant Physiol. 2009;150:320–332.

3. Nesemann K, Braus-Stromeyer SA, Harting R, Höfer A, Kusch H, Ambrosio AB, et al. Fluorescent pseudomonads pursue media-dependent strategies to inhibit growth of pathogenic Verticillium fungi. Appl Microbiol Biotechnol. 2018;102.

4. Tran V-TT, Braus-Stromeyer SA, Kusch H, Reusche M, Kaever A, Kühn A, et al. Verticillium transcription activator of adhesion Vta2 suppresses microsclerotia formation and is required for systemic infection of plant roots. New Phytol. 2014;202:565–581.

5. Starke J, Harting R, Maurus I, Bremenkamp R, James W. Unfolded protein response and scaffold independent pheromone MAP kinase signalling control *Verticillium dahliae* growth, development and plant pathogenesis. bioRxiv. 2020;doi: 10.1101/2020.02.10.941450.
