# Supplementary material for: The velvet protein Vel1 controls initial plant root colonization and conidia formation for xylem distribution in Verticillium wilt
Source: PLoS Genet. 2021 Mar 15;17(3):e1009434. doi: 10.1371/journal.pgen.1009434 (PMC7993770; doi:10.1371/journal.pgen.1009434)
Supplement: S10 Table — (PDF) [file pgen.1009434.s034.pdf]

S10 Table. Primers used in this study.

Primer name	Sequence 5' to 3'	Size	Overhang to/ restriction site
AO3	ATG GAC CGA CCC TCG AAT C	19 mer	-
AO11	ATG TCC GCC ACC ACC AT	17 mer	-
AO12	TCA TTT TGT GAA AAT AGG CGT GT	23 mer	-
AO13	AAC CCT TCT TCT GCG CT	17 mer	-
AO14	ACC ACC GCT ACC ACC TTT TGT GAA AAT AGG CGT GTA CT	38 mer	Linker
AO15	TGA GCA GAC ATC ACC ATG TCC GCC ACC ACC AT	32 mer	*GPDA* promoter
AO18	GTA TGT TGT GTG GAA CCT TAC CGC AAG CAT CTC GG	35 mer	pME4564
AO19	AGA TCC CCG GGT ACC GGT GTC TGG CGT CAG AAT GTG	36 mer	*GPDA* promoter
AO20	AGG TAA TCC TTC TTT TGC AGC ACC TTG TGA TGC G	34 mer	*TRPC* terminator
AO21	CAC AGT ACA CGA GGA TGG AGT TTG TTG CTT CTC CT	35 mer	pME4564
AO24	GAG CGA CGC CTC TTG CTT G	19 mer	-
AO25	GCT GGA TAT CCC TCT CGG G	19 mer	-
AO28	TGA GCA GAC ATC ACC ATG GAC CGA CCC TCG AAT CC	35 mer	*GPDA* promoter
AO29	ACC ACC GCT ACC ACC AAC CCT TCT TCT GCG CTT CT	35 mer	Linker
AO30	GTA TGT TGT GTG GAA AAC TAG CCC CCA TCG AC	32 mer	pME4564
AO31	ACC GGT CAC TGT ACA GGT GGA GGG GAC ACC AAG	33 mer	*GPDA* promoter
AO32	AGG TAA TCC TTC TTT TTG GGA TAT ACA GGA CTA TTT G	37 mer	*TRPC* terminator
AO33	CAC AGT ACA CGA GGA ATC GGC CAG AGT ACG TC	32 mer	pME4564
AO101	ATT CTT AAT TAA GAT CCT TAC CGC AAG CAT CTC G	34 mer	pME4564
AO136	ACC GGT CAC TGT ACA TCA TTT TGT GAA AAT AGG CGT	36 mer	*GPDA* promoter
AO137	AGG TAA TCC TTC TTT TGC AGC ACC TTG TGA TG	32 mer	*TRPC* terminator
AO138	AGG ACT TCT AGA AGG CTT GAC AAG CCA AGT CGT TG	35 mer	pME4564
AO140	AGG TAA TCC TTC TTT TGC AGC ACC TTG TGA TG	32 mer	*TRPC* terminator
AO157	ATT CTT AAT TAA GAT AAC TAG CCC CCA TCG AC	32 mer	pME4564
AO158	AAG ATC CCC GGG TAC TCA AAC CCT TCT TCT GCG	33 mer	*GPDA* promoter

S10 Table. Primers used in this study, continued.

**Primer name**	**Sequence 5’ to 3’**	**Size**	**Overhang to/ restriction site**
AO159	AGG TAA TCC TTC TTT TTG GGA TAT ACA GGA CTA TTT	36 mer	*TRPC* terminator
AO160	AGG ACT TCT AGA AGG ATC GGC CAG AGT ACG TC	32 mer	pME4564
AO165	GGT GGT AGC GGT GGT GT	17 mer	-
AO166	ACC ACC GCT ACC ACC TTT TGT GAA AAT AGG CGT GTA CT	38 mer	Linker
AO167	ATT CTT AAT TAA GAT TGC TTG CCA TCT TGC TAC ACC	36 mer	pME4564
AO168	ACC ACC GCT ACC ACC ATA ATC GTC ATC GTC GTC A	34 mer	Linker
AO169	AGG TAA TCC TTC TTT CTA GAA TGA GGC GCT GGT	33 mer	*TRPC* terminator
AO170	AGG ACT TCT AGA AGG TGC ATC TTC AGA CAC GCA	33 mer	pME4564
AO171	ACC ACC GCT ACC ACC AAC CCT TCT TCT GCG CT	32 mer	Linker
AO174	AGG ACT TCT AGA AGG TGG AGT TTG TTG CTT CTC	33 mer	pME4564
AO175	ACC GGT CAC TGT ACA GGT GTC TGG CGT CAG AA	32 mer	*GPDA* promoter
AO176	ATT CTT AAT TAA GAT CCT GGA GTA CTC TGC GCA	33 mer	pME4564
AO177	ACC ACC GCT ACC ACC GGA GTA GTC CCG ACC CCA	33 mer	Linker
AO178	AGG TAA TCC TTC TTT TGA TGT TAC TTG CGA GAC TCG	36 mer	*TRPC* terminator
AO179	AGG ACT TCT AGA AGG AAG CTT CCC GTC CGT TGA	33 mer	pME4564
AO191	ACC GGT CAC TGT ACA CTT GTC AAG CAA CGG CCT	33 mer	*GPDA* promoter
ML1	TTC CAC ACA ACA TAC GAG CC	20 mer	-
ML2	TCC TCG TGT ACT GTG TAA GC	20 mer	-
ML8	AAA GAA GGA TTA CCT CTA AAC AA	23 mer	-
ML9	TGT ACA GTG ACC GGT GAC	18 mer	-
ML30	GGT GGT AGC GGT GGT ATG GTG AGC AAG GGC GAG	33 mer	Linker
ML31	GGT GAT GTC TGC TCA AGC GG	20 mer	-
PC4	TGT ACA GTG ACC GGT GAC TC	20 mer	-
RH514	ACC GGT CAC TGT ACA TTA CTT GTA CAG CTC GTC CAT	36 mer	*GPDA* promoter
RH590	TGT ACA GTG ACC GGT GA	17 mer	-

S10 Table. Primers used in this study, continued.

Primer name	Sequence 5' to 3'	Size	Overhang to/ restriction site
RO3	GGT ACC CGG GGA TCT TTC G	19 mer	-
RO4	AAA GAA GGA TTA CCT CTA AAC AA	23 mer	-
SB40	ACC ATA GTT TGG TGG ATG CAA G	22 mer	-
SB41	CGA CGG GTC ACC ATG ATG	18 mer	-
SZ9	*AAC ACC CAG AAC AAG ATG CGC*	21 mer	-
SZ10	GCT TGA CCT TGA GAT CCT TG	20 mer	-
SZ11	TGC ATT CTT GGC AAG AGA TGT GTG	24 mer	-
SZ12	AGC TTG TTA TCC TTG TCC TCG GT	23 mer	-
SZ19	ACC TCT GGA GGC AAG GCT T	19 mer	-
SZ20	GCT TGG CCT TCT TCT TCT GC	20 mer	-
VEL2gfp-F	GGG CTC GAG ATG AGC TAC GAC CAG CAC CA	29 mer	*Xho*I restriction site
VEL2gfp-R	GGG GGT ACC ATA ATC GTC ATC GTC GTC ATC C	31 mer	*Kpn*I restriction site
VEL2-P1	GGG TTA ATT AAT GCT TGC CAT CTT GCT ACA C	31 mer	*Pac*I restriction site
VEL2-P2	GGG ACT AGT TAC TTT GGC CGA CTC TTG CT	29 mer	*Spe*I restriction site
VEL2-P3	GGG TCT AGA GCG AGG GAG GTA GAA AAG GT	29 mer	*Xba*I restriction site
VEL2-P4	GGG CCT GCA GGG CAT CTT CAG ACA CGC AAA A	31 mer	*Sbf*I restriction site
VELB-F1	ATG AGC TAC GAC CAG CAC C	19 mer	-
VELB-R1	ATA ATC GTC ATC GTC GTC AT	20 mer	-
VOS1-P1	GGG TTA ATT AAT GTC AGG CTC CTC TCG ATT T	31 mer	*Pac*I restriction site
VOS1-P2	GGG GAT ATC GAT GTT GAA GTT CCG CTG GT	29 mer	*Eco*RV restriction site
VOS1-P3	GGG GGA TCC GCG AGA CTC GAA GTT GGA CA	29 mer	*Bam*HI restriction site
VOS1-P4	GGG GGG CCC GCC AGA GAT ACA GCG TGT GA	29 mer	*Apa*I restriction site
Vos1orf-E1	GGG CTC GAG ATG GCA GGT CTT GCC AAT	27 mer	*Xho*I restriction site
Vos1orf-E2	GGG GGT ACC GGA GTA GTC CCG ACC CCA	27 mer	*Kpn*I restriction site
ZQY3	CTG CAG GAA TTC GAT GTG ACC GGT GAC TCT TTC TG	35 mer	pBlueScript II KS
ZQY7	ATC GAT AAG CTT GAT CGA GTG GAG ATG TGG AGT GG	35 mer	pBlueScript II KS

S10 Table. Primers used in this study, continued.

Primer name	Sequence 5’ to 3’	Size	Overhang to/ restriction site
ZQY8	GTG ACC GGT GAC TCT TTC TG	20 mer	-
ZQY9	CGA GTG GAG ATG TGG AGT GG	20 mer	-
