# Supplementary material for: The velvet protein Vel1 controls initial plant root colonization and conidia formation for xylem distribution in Verticillium wilt
Source: PLoS Genet. 2021 Mar 15;17(3):e1009434. doi: 10.1371/journal.pgen.1009434 (PMC7993770; doi:10.1371/journal.pgen.1009434)
Supplement: S11 Table — (PDF) [file pgen.1009434.s035.pdf]

S11 Table. Plasmids used in this study.

Plasmid name	Description	Reference
pBlueScript II KS	Cloning vector, *AMP*^{R}	Fermentas
pGreen2	^{P}*GPDA:GFP:TRPC*^{T}:^{P}*GPDA:HYG*^{R}*:TRPC*^{T}*; KAN*^{R}<br>Cloning vector with left and right border for ATMT,<br>*Eco*RV restriction site	[1]
pKO2	^{P}*GPDA:NAT*^{R}*; KAN*^{R}<br>Cloning vector with left and right border for ATMT<br>*Eco*RV restriction site	[2]
pPK2	^{P}*GPDA:HYG*^{R}*:TRPC*^{T}*; KAN*^{R}<br>Cloning vector with left and right border for ATMT,<br>*Eco*RV restriction site	[3]
pME4548	^{P}*TRPC:NAT*^{R}*; KAN*^{R}<br>Cloning vector with left and right border for ATMT,	[4]
pME4564	^{P}*TRPC:HYG*^{R}*; KAN*^{R}<br>Cloning vector with left and right border for ATMT,	[2]
pME4815	^{P}*GPDA:NAT*^{R}*:TRPC*^{T} in pME4564	[5]
pME4819	^{P}*GPDA:GFP:TRPC*^{T} in pME4815	[5]
pME4975	^{P}*GPDA:RFP:H2B:TRPC*^{T}<br>in pPK2, left and right border for ATMT	[5]
pME4976	^{P}*GPDA:RFP:H2B:TRPC*^{T},<br>left and right border for ATMT	[5]
pME4990	^{P}*GPDA:NLP3:GFP:TRPC*^{T}:^{P}*GPDA:HYG*^{R}*:TRPC*^{T}<br>in pPK2, left and right border for ATMT	[2]
pME5063	^{P}*VEL1:*^{P}*GPDA:HYG*^{R}*:TRPC*^{T}*:VEL1*^{T} in pME4564,<br>left and right border for ATMT	This study
pME5064	^{P}*VEL1:VEL1:*^{P}*GPDA:NAT*^{R}*:TRPC*^{T}*:VEL1*^{T} in pME4564,<br>left and right border for ATMT	This study
pME5065	^{P}*VEL1:*^{P}*GPDA:NAT*^{R}*:TRPC*^{T}*:VEL1*^{T} in pME4564,<br>left and right border for ATMT	This study
pME5066	^{P}*VEL2:*^{P}*TRPC:NAT*^{R}*:VEL2*^{T} in pME4548,<br>left and right border for ATMT	This study
pME5067	^{P}*VEL2:VEL2:GFP:*^{P}*GPDA:HYG*^{R}*:TRPC*^{T}*:VEL2*^{T} in pME4564,<br>left and right border for ATMT	This study
pME5068	^{P}*VEL3:*^{P}*GPDA:NAT*^{R}*:TRPC*^{T}*:VEL3*^{T} in pME4564,<br>left and right border for ATMT	This study
pME5069	^{P}*VEL3:VEL3:*^{P}*GPDA:HYG*^{R}*:TRPC*^{T}*:VEL3*^{T} in pME4564,<br>left and right border for ATMT	This study
pME5070	^{P}*VOS1:*^{P}*GPDA:NAT*^{R}*:VOS1*^{T} in pNAT1,<br>left and right border for ATMT	This study
pME5071	^{P}*VOS1:*^{P}*GPDA:HYG*^{R}*:TRPC*^{T}*:VOS1*^{T} in pME4564,<br>left and right border for ATMT	This study
pME5072	^{P}*VEL1:VEL1:GFP:*^{P}*GPDA:HYG*^{R}*:TRPC*^{T}*:VEL1*^{T} in pME4564,<br>left and right border for ATMT	This study
pME5073	^{P}*VEL3:VEL3:GFP:*^{P}*GPDA:HYG*^{R}*:TRPC*^{T}*:VEL3*^{T} in pME4564,<br>left and right border for ATMT	This study
pME5074	^{P}*VOS1:VOS1:GFP:*^{P}*GPDA:HYG*^{R}*:TRPC*^{T}*:VOS1*^{T}<br>in pME4564, left and right border for ATMT	This study
pME5078	^{P}*GPDA:VEL1:GFP:TRPC*^{T} in pGreen2,<br>left and right border for ATMT	This study

S11 Table. Plasmids used in this study, continued.

Plasmid name	Description	Reference
pME5079	^{P}*GPDA:VEL1:GFP:TRPC*^{T}*:*^{T}*TRPC:HYG*^{R}*:GPDA*^{P} in pPK2, left and right border for ATMT	This study
pME5080	^{P}*GPDA:HYG*^{R}*:TRPC*^{T}*:*^{P}*GPDA:VEL2:GFP:TRPC*^{T} in pGreen2, left and right border for ATMT	This study
pME5081	^{P}*GPDA:VEL3:GFP:TRPC*^{T} in pGreen2, left and right border for ATMT	This study
pME5082	^{P}*GPDA:VEL3:GFP:TRPC*^{T} in pBlueScript II KS	This study
pME5083	^{P}*GPDA:VEL3:GFP:TRPC*^{T}*:*^{P}*GPDA:HYG*^{R}*:TRPC*^{T} in pPK2, left and right border for ATMT	This study
pME5084	^{P}*GPDA:HYG*^{R}*:TRPC*^{T}*:*^{P}*GPDA:VOS1:GFP:TRPC*^{T} in pGreen2, left and right border for ATMT	This study

ATMT=*Agrobacterium tumefaciens*-mediated transformation, ^{P}=promoter, ^{T}=terminator, *NAT*^{R}=nourseothricin resistance marker, *HYG*^{R}=hygromycin B resistance marker, *AMP*^{R}=ampicillin resistance marker, *KAN*^{R}=kanamycin resistance marker, *GEN*^{R}=geneticin resistance

References

1. Tran V-TT, Braus-Stromeyer SA, Kusch H, Reusche M, Kaever A, Kühn A, et al. Verticillium transcription activator of adhesion Vta2 suppresses microsclerotia formation and is required for systemic infection of plant roots. New Phytol. 2014;202:565–581.

2. Leonard M, Kühn A, Harting R, Maurus I, Nagel A, Starke J, et al. *Verticillium longisporum* elicits media-dependent secretome responses with capacity to distinguish between plant-related environments. Front Microbiol. 2020;11:1876.

3. Covert SF, Kapoor P, Lee M, Briley A, Nairn CJ. *Agrobacterium tumefaciens*-mediated transformation of *Fusarium circinatum*. Mycol Res. 2001;105:259–264.

4. Bui T, Harting R, Braus-Stromeyer SA, Tran V, Leonard M, Höfer A, et al. *Verticillium dahliae* transcription factors Som1 and Vta3 control microsclerotia formation and sequential steps of plant root penetration and colonisation to induce disease. New Phytol. 2019;221:2138–2159.

5. Starke J, Harting R, Maurus I, Bremenkamp R, James W. Unfolded protein response and scaffold independent pheromone MAP kinase signalling control *Verticillium dahliae* growth, development and plant pathogenesis. bioRxiv. 2020;doi: 10.1101/2020.02.10.941450.
