# Supplementary material for: The velvet protein Vel1 controls initial plant root colonization and conidia formation for xylem distribution in Verticillium wilt
Source: PLoS Genet. 2021 Mar 15;17(3):e1009434. doi: 10.1371/journal.pgen.1009434 (PMC7993770; doi:10.1371/journal.pgen.1009434)
Supplement: S12 Table — (PDF) [file pgen.1009434.s036.pdf]

S12 Table. Perseus workflow for evaluation of the MaxQuant result files for the *in vitro* protein pull-downs.

Step	Command	Description
1	Generic matrix upload	Upload proteingroups.txt<br>Main: LFQ intensities<br>Numerical: MS/MS counts, sequence coverage, unique peptides, razor + unique peptides
2	Filter rows based on categorical column	Remove rows with + for Only identified by site<br>Remove rows with + for Reverse<br>Remove rows with + for Potential contaminant
3	Rearrange	Remove empty columns
4	Transform	LFQ intensities = $\log_2(x)$
5	Categorical annotation rows	Groups: wild type control and examined velvet strain(s)
6	Analysis	Multi scatter plot<br>Numeric Venn diagram
7	Filter rows based on valid values	Min. valids 3<br>in at least one group: examined velvet strain<br>Values: valid<br>Reduce matrix
8	Replace missing values from normal distribution	Mode: Total matrix
9	Analysis	Volcano Plot<br>First group: Velvet strain<br>Second group: wild type
10	Repeat step 8 and 9 at least 3 times	
11	Load significant interaction partners in Venny 2.1 [1] and generate a list of proteins found in all four repetitions of the statistical analysis as significant (step 8 and 9)	
12	Select rows with significant hits found in all four repetitions as co-enriched with the bait based on the result of step 11	Export selection (reduce matrix)
13	Select rows with significant hits found in all three repetitions as co-enriched with the bait	Export selection (reduce matrix)
14	Replace imputed values by NaN	Export Matrix, see S5-8 Tables
15	Select representative Volcano Blot	Label significant partners (found in all four repetitions)

References

1. Oliveros JC. Venny. An interactive tool for comparing lists with Venn`s diagrams; 2015 [cited 2020 July] [Internet]. Available from: https://bioinfogp.cnb.csic.es/t.
